# Ion-Responsive Microneedles Delivering Subtype-Specific Mitochondrial Extracellular Vesicles from HEY1⁺ Cardiomyocytes for Cardiac Repair in Bama Minipigs with Myocardial Ischemia–Reperfusion Injury

**DOI:** 10.7150/thno.123209

**Published:** 2026-06-10

**Authors:** Peng Qu, Jiao Shi, Xue Li, Yao Gu, Jun Liu, Hongyan Zhang, MingZhi Zhou, Cui Ma, Xinghui Li, Wenjie Tian, Qi Liang, Gang Li, Panke Cheng

**Affiliations:** 1 Department of Clinical Laboratory, Affiliated Hospital of North Sichuan Medical College, Nanchong 637000, China.; 2 School of Laboratory Medicine, North Sichuan Medical College, Nanchong 637007, China.; 3 Translational Medicine Research Center, North Sichuan Medical College, Nanchong 637007, China.; 4 Institute of Cardiovascular Diseases & Department of Cardiology, Sichuan Provincial People's Hospital, School of Medicine, University of Electronic Science and Technology of China, Chengdu 610072, China; 5 Department of Anesthesiology, Chengdu Wenjiang District People's Hospital, Chengdu 611130, China; 6 Department of Mathematics, Army Medical University, Chongqing 400038, China.; 7 Department of Radiology, Affiliated Hospital of North Sichuan Medical College, Nanchong 637000, China; 8 Medical Imaging Key Laboratory of Sichuan Province, Nanchong 637007, China.

**Keywords:** Ischemia-reperfusion injury, HEY1, P5CS, ATP5B, Microneedles

## Abstract

The pathogenesis of myocardial ischemia-reperfusion (MI/R) injury is intricately linked to mitochondrial dysfunction occurring during both the ischemic and reperfusion phases. Through single-cell transcriptome analysis, we identified a subpopulation of HEY1-high expressing cardiomyocytes (HEY1^+^ CMs) characterized by superior mitochondrial homeostasis. To leverage this, we isolated P5CS-type or ATP5B-type functional mitochondria from a ΔΨ_m_-high subpopulation, which was obtained via membrane potential sorting following dual overexpression in HEY1^+^ CMs, and subsequently encapsulated them within HEY1^+^ CM-derived exosomes to achieve stable, targeted delivery. We designed a responsive microneedle patch based on local copper/iron ion dynamics to enable the stage-specific release of these mitochondria within the ischemic or reperfusion microenvironments. In a Bama minipig MI/R model, this system significantly ameliorated cardiac function, reduced infarct size, and attenuated cardiomyocyte death. Mechanistically, the therapeutic strategy enhanced mitochondrial structural integrity and energy metabolic function. This study establishes a responsive, stage-specific mitochondrial delivery platform, offering a promising strategy for the precision treatment of ischemic heart disease.

## Introduction

Myocardial ischemia-reperfusion (MI/R) injury is the pivotal mechanism of secondary myocardial damage following reperfusion therapy for acute myocardial infarction, and its severity is closely linked to long-term prognosis^1^. Mitochondria are involved throughout the entire MI/R process, exhibiting a biphasic functional imbalance: during the ischemic phase, oxidative phosphorylation is inhibited, leading to a global downregulation of energy metabolism; upon reperfusion, the sudden reintroduction of oxygen triggers a rapid restoration of the electron transport chain, inducing a burst of reactive oxygen species (ROS)^2^. This process further triggers the collapse of mitochondrial membrane potential (ΔΨ_m_), intracellular calcium overload, and the opening of the mitochondrial permeability transition pore (mPTP), ultimately driving programmed cell death and exacerbating structural and functional myocardial damage^3^. While current strategies targeting antioxidants, autophagy modulation, or mPTP inhibition have shown protective effects in animal models, their clinical translation remains limited. A major bottleneck is the lack of a temporally resolved delivery system capable of stage-specific release in response to the transition from ischemia to reperfusion while ensuring localized enrichment within the myocardium. Accumulating evidence indicates that cardiomyocytes are not a monolithic population but rather exhibit significant heterogeneity in metabolic activity, mitochondrial density, and stress response capacity^4^. Within MI/R models, distinct subpopulations display divergent metabolic profiles and susceptibility to injury^5^. Spatial single-cell studies have revealed "survivor-like" cell populations with unique transcriptional and metabolic signatures within the infarct and border zones, suggesting the intrinsic existence of a subset of cardiomyocytes^6^, ^7^.

Mitochondria exhibit functional heterogeneity, differentiating into distinct subtypes based on local ATP demand and stress microenvironments: P5CS-associated mitochondria are predisposed to maintaining redox homeostasis via metabolic pathways such as the glutamate cycle under oxidative stress, whereas ATP synthase (ATP5B)-associated mitochondria are specialized for efficient ATP synthesis to sustain energy supply^8^. However, mere differentiation of subtypes is insufficient to guarantee therapeutic efficacy; it necessitates the implementation of quality control based on mitochondrial membrane potential (ΔΨ_m_) to exclude damaged organelles and ensure the enrichment of high-activity populations^9^. This provides a refined model for stage-specific MI/R intervention: prioritizing the supplementation of high-ΔΨ_m_ P5CS mitochondria during the ischemic phase to enhance tolerance, and high-ΔΨ_m_ ATP5B mitochondria during reperfusion to facilitate energy recovery. In contrast to free mitochondria, which suffer from limited stability^10^, extracellular vesicles (EVs) serve as natural nanocarriers with better stability and delivery adaptability, capable of transporting mitochondrial components and facilitating intercellular functional transfer. Notably, EVs derived from metabolically active cardiomyocytes frequently demonstrate enhanced therapeutic potential^11^. Beyond payload quality, achieving temporal control synchronized with the MI/R progression is equally critical. Local copper and iron ion levels exhibit dynamic fluctuations during MI/R: the ischemic phase is characterized by elevated Cu^2+^ concentrations, whereas the reperfusion phase features a rapid surge in Fe^2+^ accompanied by amplified oxidative stress^12^. Constructing programmable release switches based on these dynamic metal ion signals provides a natural trigger mechanism for stage-specific delivery. Previous studies have developed various metal ion-responsive peptides for stimuli-responsive release—such as GGH for Cu^2+^-triggered release and HGHGHGHG for Fe^2+^-triggered release—thereby initiating the therapeutic release process at distinct pathological stages^13^, ^14^. Integrating metal ion-responsive mechanisms with functional EV delivery holds promise for achieving temporal regulation of mitochondrial homeostasis, tailoring the therapeutic process to the dynamic pathological demands of MI/R.

Regarding spatial delivery, microneedle patches (MNs) are gaining increasing attention in the field of cardiac targeted therapy due to their high penetrability, tissue retention, and biocompatibility^15^. This system allows for the direct release of therapeutic factors onto the epicardium by traversing the pericardium in a minimally invasive manner, establishing a local micro-reservoir to prolong retention and release duration, thereby enhancing local concentration and minimizing systemic risks. Multilayer microneedle structures constructed from materials such as hyaluronic acid (HA), polycaprolactone-block-polyacrylic acid (PCL-b-PAA), and polydopamine (PDA) offer both substantial capacity for functionalization and the flexibility to incorporate environmentally responsive mechanisms for sequential release^16^. Furthermore, the incorporation of a hydrogel backing barrier minimizes off-target diffusion, thereby improving delivery precision and safety.

**Scheme 1:**
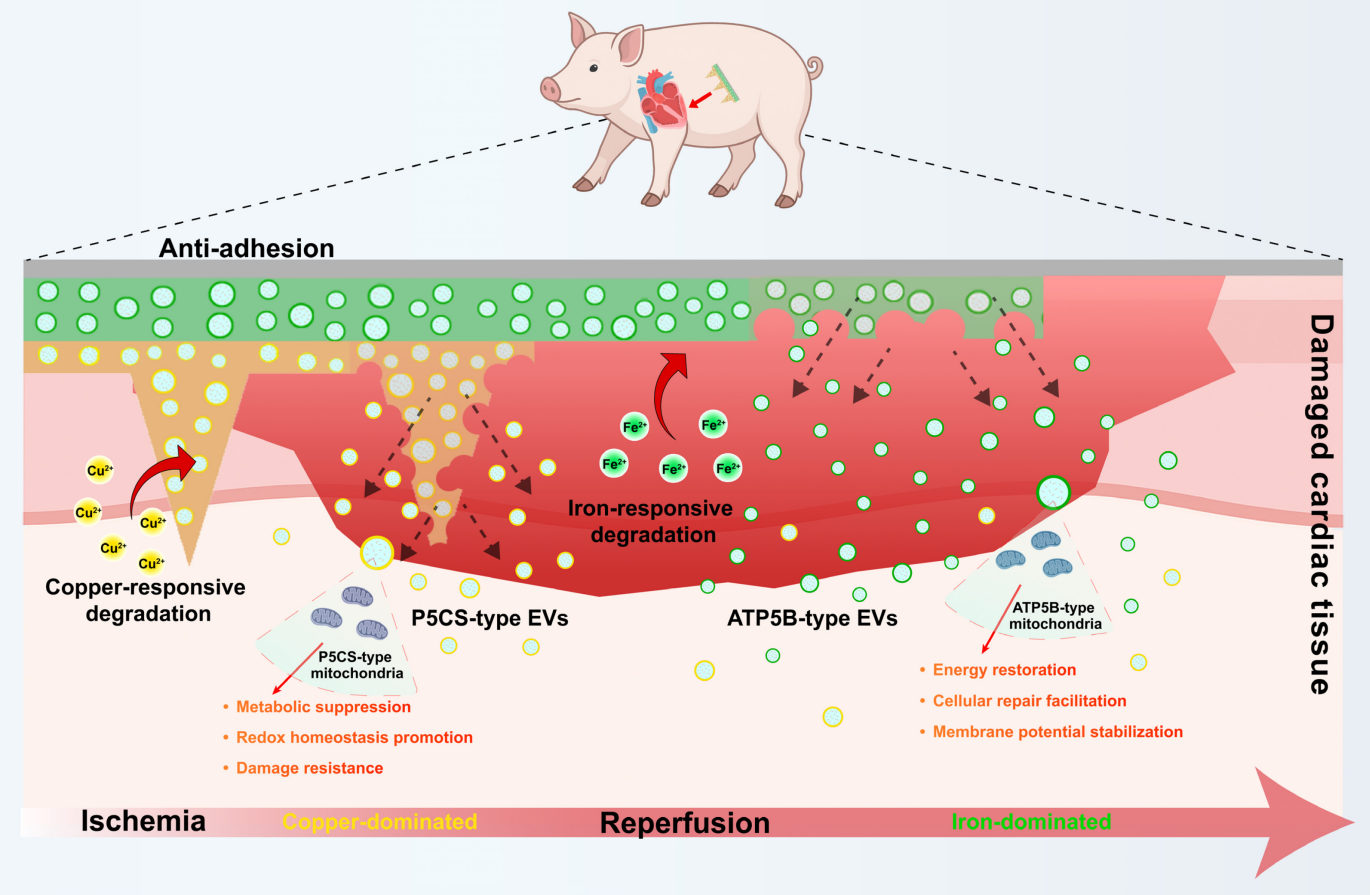
** Schematic illustration of the metal ion-responsive microneedle patch.** This platform is designed for stage-specific exosome delivery and myocardial protection during ischemia/reperfusion. An anti-adhesion layer on the patch surface minimizes postoperative tissue adhesion. Within the injured myocardial microenvironment, the predominance of copper ions during early ischemia triggers the degradation of the PPC layer, prioritizing the release of P5CS-EVs to support redox homeostasis and injury resistance. Subsequently, the predominance of iron ions during the reperfusion phase triggers the degradation of the HPF layer, facilitating the release of ATP5B-EVs to promote energy metabolism recovery, cellular repair, and membrane potential stabilization. By aligning local, sequential delivery with the pathological chronology, this system achieves comprehensive protection against myocardial ischemia-reperfusion injury.

In this study, we identified an injury-resistant cardiomyocyte subpopulation characterized by high HEY1 expression, which exhibits robust mitochondrial maintenance capacity during MI/R. By employing iPSC-directed differentiation to generate HEY1^+^ CMs, we established a donor selection and quality control workflow centered on ΔΨ_m_, thereby producing highly active engineered EVs **selectively** enriched with either P5CS or ATP5B. Leveraging dynamic metal ion signals, we designed a multilayer microneedle platform synchronized with the MI/R progression: P5CS-type EVs were loaded into a Cu^2+^-responsive module to address anti-stress requirements during the ischemic phase, while ATP5B-type EVs were loaded into an Fe^2+^-responsive module to facilitate energy reconstitution during reperfusion. In a Bama minipig MI/R model, this system significantly improved mitochondrial function, attenuated necrosis, and enhanced cardiac performance, demonstrating **good** translational potential. (As illustrated in **Scheme 1**.)

## Materials and Methods

### Assessment of Mitochondrial Function

Mitochondrial Calcium Overload. Cells were incubated with 5 μM Fluo-3 AM (Beyotime) in serum-free medium at 37 °C for 30 min, followed by washing with PBS. Fluorescence intensity was measured using a microplate reader (excitation: 488 nm; emission: 525 nm). Mitochondrial Membrane Potential. Cells were stained with JC-1 working solution (Beyotime) at 37 °C for 20 min, washed with buffer, and analyzed by microplate reader. Red fluorescence (aggregates) was measured at 525/590 nm, and green fluorescence (monomers) at 490/530 nm. The red/green ratio was used to assess MMP. Mitochondrial Permeability Transition Pore (mPTP) Opening. Cells were incubated with 1 μM Calcein-AM and 1 mM CoCl₂ (Thermo Fisher) for 15 min at 37 °C. After washing, calcein fluorescence was detected using a microplate reader (excitation: 488 nm; emission: 515 nm). Decreased signal indicated mPTP opening. Mitochondrial ROS. Cells were incubated with 5 μM MitoSOX™ Red (Thermo Fisher) for 15 min at 37 °C in the dark, washed with PBS, and analyzed immediately by flow cytometry using PE channel. Mitochondrial Morphology. Cells were stained with MitoTracker™ Red CMXRos (200 nM, Thermo Fisher) for 30 min at 37 °C, fixed with 4% paraformaldehyde, and imaged using a confocal microscope. Mitochondrial length was quantified using ImageJ software from ≥ 50 cells per group.

### Fabrication of Microneedle Patches (MN and MN(MixEvs))

To fabricate the microneedle patches, either the PPC pre-gel solution or the extracellular vesicle-loaded formulation (PPC_Ev(MT^P5CS^)) was prepared in a flowable state by dispersion in aqueous medium. A 20 μL aliquot of the PPC mixture was carefully dispensed per mold into each microneedle cavity of a PDMS mold using a precision micropipette. The mold featured a microneedle array geometry of 10 × 10 with individual needle cones measuring approximately 300 μm in height and 150 μm in base diameter. Immediately after loading, the mold was centrifuged at 3000 rpm for 5 minutes at 4°C to ensure complete filling of the needle tip regions. Without allowing the PPC layer to solidify, a second layer comprising the HPF precursor solution (with or without Ev(MT^ATP5B^)) was promptly applied on top. Approximately 60 μL of HPF solution per mold was added to ensure full occupation of the middle and basal regions of the needles. A second centrifugation was then carried out at 2000 rpm for 3 minutes to facilitate interpenetration and interface integration between the PPC and HPF layers. The mold was subsequently transferred to a 4 °C environment or placed in an ice bath for 4 hours to allow partial crosslinking and stabilization of the bilayer structure. Separately, a PCA prepolymer solution was prepared by mixing PEGDA, AA, and C-CNCs at a mass ratio of 4:2:1, with 1 wt% Irgacure 2959 as the photoinitiator. While the HPF layer remained hydrated, the PCA solution was gently spread across the back surface of the mold to form a uniform coating layer. A sterile glass slide was carefully placed over the mold and lightly pressed to ensure a smooth and even contact surface. Photocrosslinking was then initiated using ultraviolet light (365 nm, 10 mW/cm²) for 10 minutes to solidify the PCA barrier layer. After crosslinking, the entire three-layer structure was allowed to stabilize at 4 °C for an additional hour to ensure complete network formation throughout all layers. The microneedle array was then carefully demolded by peeling away the PDMS template, yielding an integrated microneedle patch, either MN or MN(MixEvs), depending on the Evs content. Finally, the assembled microneedle patches were freeze-dried at -40 °C for 12 hours to facilitate long-term storage or further downstream applications.

### Statistical Analysis

Data are presented as mean ± standard deviation (SD). Sample sizes (n) for independent experiments are indicated in figure legends or as individual data points. Comparisons between two groups were performed using unpaired two-tailed Student's t-tests. Multiple group comparisons were analyzed using one-way analysis of variance (ANOVA) followed by Tukey's post hoc test. For two-factor designs, two-way ANOVA with Sidak's or Tukey's multiple comparisons test was employed. A P-value of < 0.05 was considered statistically significant. All statistical analyses were performed using GraphPad Prism 9.0 software.

## Results and Discussion

### Identification of the HEY1^+^ Cardiomyocyte Subpopulation and Mechanistic Evidence of Its Mitochondrial Homeostatic Superiority

**Figure 1:**
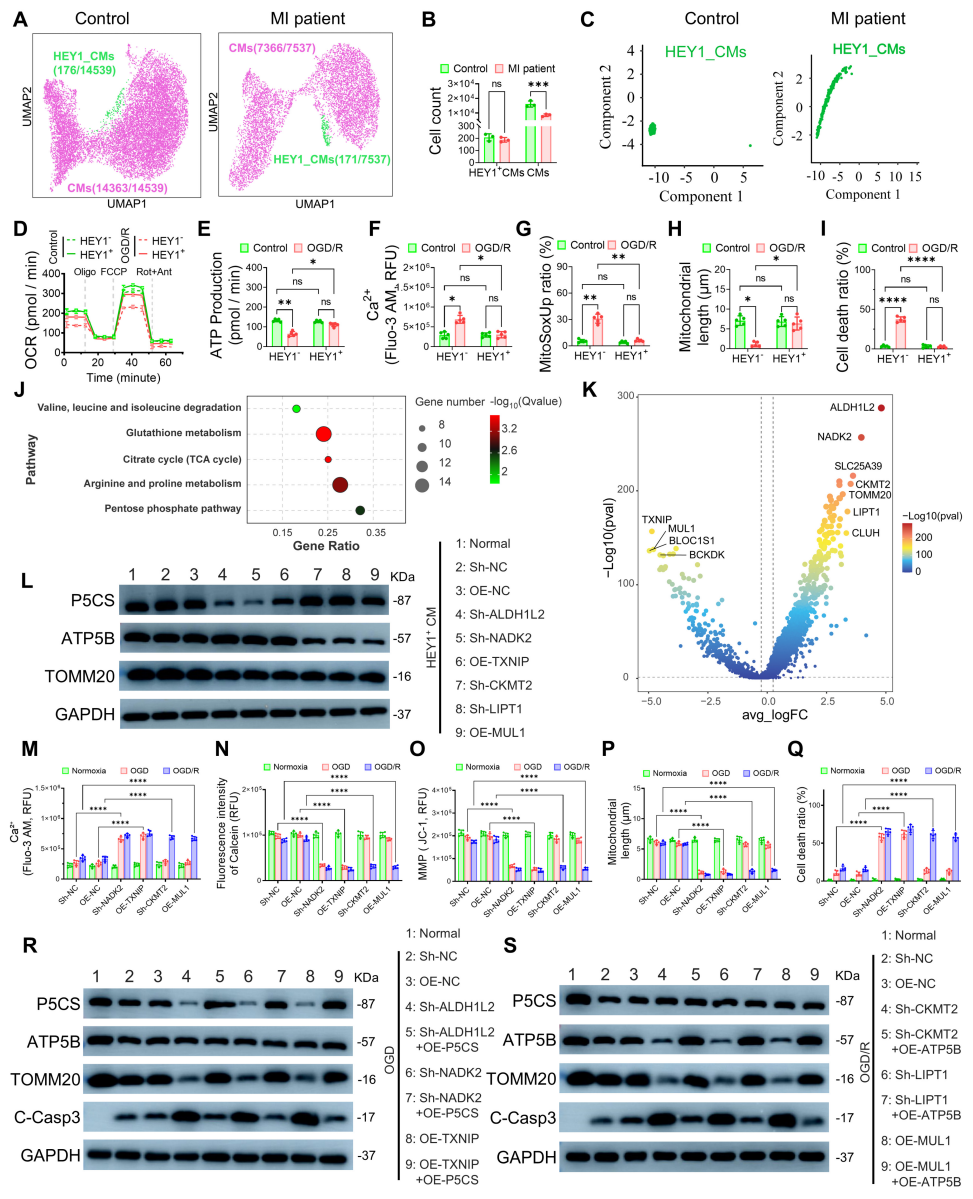
** Identification, mitochondrial functional characterization, and metabolic regulatory mechanism analysis of the HEY1^+^ cardiomyocyte subpopulation. (A)** UMAP visualization of scRNA-seq data showing the distribution of HEY1-high expressing cardiomyocytes (HEY1^+^ CMs) in heart tissues from healthy controls and MI patients. **(B)** Quantification of total cardiomyocytes and HEY1^+^ CMs in control and MI samples. **(C)** Pseudotime trajectory analysis of *HEY1^+^* CMs under control and MI conditions, revealing their developmental progression. **(D–I)** To assess mitochondrial function, HEY1-high (HEY1^+^ CMs) and *HEY1*-low (*HEY1*⁻ CMs) cardiomyocytes derived from iPSC differentiation were exposed to OGD/R treatment (1% FBS, 0.5% O₂, 1 h hypoxia followed by 12 h recovery). Evaluations included: **(D)** mitochondrial OCR, **(E)** ATP production, **(F)** intracellular Ca^2+^ levels (Fluo-3 AM fluorescence intensity), **(G)** mitochondrial ROS generation (MitoSOX), **(H)** quantitative analysis of mitochondrial length, and **(I)** cell mortality rates. **(J)** KEGG pathway enrichment analysis revealing that DEGs in the HEY1^+^ subpopulation are significantly clustered in pathways related to antioxidant stress (glutathione metabolism, pentose phosphate pathway) and mitochondrial energy provision (TCA cycle, arginine/proline metabolism). **(K)** Volcano plot of DEGs further screening for candidate upstream regulators potentially modulating the P5CS-associated ischemic adaptation axis and the ATP5B-associated reperfusion recovery axis, respectively. **(L)** Western blot analysis assessing the independence of the two metabolic regulatory modules. Protein expression levels of P5CS, ATP5B, and TOMM20 were detected in *HEY1^+^* CMs following knockdown (Sh-) or overexpression (OE-) of potential key regulators (ALDH1L2, NADK2, TXNIP, CKMT2, LIPT1, MUL1). **(M–Q)** Comparison of cellular functional changes under normoxia, OGD, and OGD/R conditions following the aforementioned knockdown or overexpression treatments. Indicators include: **(M)** intracellular Ca^2+^ levels, **(N)** extent of mPTP opening, **(O)** MMP, **(P)** mitochondrial length, and **(Q)** cell mortality rates. **(R–S)** Western blot evaluation of the rescue effects in key protein restoration experiments. **(R)** Cells with P5CS module-associated perturbations (Sh-ALDH1L2, Sh-NADK2, OE-TXNIP) were rescued by P5CS overexpression under OGD conditions. **(S)** Cells with ATP5B module-associated perturbations (Sh-CKMT2, Sh-LIPT1, OE-MUL1) were rescued by ATP5B overexpression under OGD/R conditions. Expression of P5CS, ATP5B, TOMM20, and the apoptosis marker C-Casp3 was detected to verify the impact of restoring specific mitochondrial subtypes on mitochondrial integrity and cell survival.** Statistics**: Data are presented as mean ± SD; the independent sample size (*n*) is indicated by data points/labels in the figures. One-way ANOVA with Tukey’s multiple-comparison correction was used for single-factor multi-group comparisons. Two-way ANOVA with Sidak’s or Tukey’s multiple-comparison correction was used for two-factor designs. *P < 0.05, **P < 0.01, ***P < 0.005, ****P < 0.001; *ns*, not significant.

To investigate cellular heterogeneity following myocardial infarction (MI), we performed single-cell RNA sequencing (scRNA-seq) analysis on myocardial tissues from MI patients and non-MI controls (**Figure S1A**). Dimensionality reduction and clustering analysis revealed the presence of a stable cardiomyocyte subpopulation characterized by high HEY1 expression (HEY1^+^ CMs) in both groups (**Figure 1A**). Notably, while the total cardiomyocyte population significantly declined post-MI, the absolute count of HEY1^+^ CMs remained largely unaffected (**Figure 1B**), with HEY1 gene expression maintained at high levels even under pathological conditions (**Figure S1B**). Pseudotime analysis further demonstrated that HEY1^+^ CMs are positioned at an earlier stage of the developmental trajectory compared to terminally differentiated conventional cardiomyocytes (**Figures 1C, S1C**). Given that HEY1, a canonical downstream transcription factor of the Notch signaling pathway, is established to play a critical role in maintaining cell stemness, regulating mitochondrial metabolism, and counteracting oxidative stress^17^, this developmental feature, combined with their retention advantage within the injury microenvironment, suggests that HEY1^+^ CMs represent a distinct functional subpopulation possessing both primitive homeostatic reserves and intrinsic ischemic resilience. To validate the functional characteristics of this subpopulation, we directed the differentiation of induced pluripotent stem cells (iPSCs) into HEY1 high-expression (HEY1⁺CM) and low-expression (HEY1⁻CM) cardiomyocytes (**Figure S1D**) and established an oxygen-glucose deprivation/reoxygenation (OGD/R) model to simulate ischemia-reperfusion injury. Oxygen consumption rate (OCR) assays revealed that HEY1^+^ CMs exhibited more robust overall respiratory kinetics (**Figure 1D**), maintaining stable ATP production (**Figure 1E**), basal respiration (**Figure S1E**), maximal respiratory capacity (**Figure S1F**), and lower proton leakage (**Figure S1G**) under OGD/R stress. In contrast, HEY1⁻ CMs displayed extensive mitochondrial dysfunction, including intracellular calcium overload (**Figure 1F**), excessive ROS production (**Figures 1G, S2A**), loss of mitochondrial membrane potential (**Figure S1H**), and increased mPTP opening (**Figure S1I**). Confocal imaging and flow cytometry further confirmed that HEY1^+^ CMs maintained an intact mitochondrial network morphology (**Figures 1H, S2B**) and exhibited a significantly lower apoptosis rate compared to the control group (**Figures 1I, S2C**). Collectively, these phenotypic analyses further substantiate that the cardiomyocyte subpopulation characterized by HEY1^+^ CMs inherently possesses superior resilience to metabolic stress and mitochondrial homeostatic reserve. Previous studies have indicated that HEY1^+^ CMs exhibit mitochondrial functional heterogeneity, characterized by distinct enrichment of P5CS (associated with redox balance) and ATP5B (associated with energy supply)^8^. To elucidate the molecular basis of this tolerance advantage, we performed pathway analysis on the differentially expressed genes (DEGs) of HEY1^+^ CMs. Kyoto Encyclopedia of Genes and Genomes (KEGG) enrichment analysis revealed that DEGs were primarily clustered into two distinct metabolic modules: one related to antioxidant defense and redox maintenance (e.g., glutathione metabolism, pentose phosphate pathway), and the other related to mitochondrial energy provision (e.g., TCA cycle and amino acid metabolism) (**Figure 1J**). Building on this, we utilized a volcano plot of the DEGs to further screen for candidate key regulators potentially driving these metabolic characteristics, which were subjected to functional verification in subsequent experiments (**Figure 1K**). To validate the basal metabolic reserve of HEY1^+^ CMs, we assessed the expression of key proteins across diverse cellular contexts and under HEY1 knockdown conditions. Under basal conditions, HEY1^+^ CMs exhibited significantly elevated levels of key regulators, including P5CS, ATP5B, TOMM20, and NADK2; whereas specific silencing of HEY1 did not significantly alter the abundance of these proteins (**Figure S2D**). These results confirm that HEY1^+^ CMs possess a pre-existing high mitochondrial content and dual metabolic reserves, providing the metabolic foundation for their stress tolerance. Furthermore, this suggests that HEY1 likely serves as an identity marker for this subpopulation rather than acting as the sole determinant driving the mitochondrial phenotype. Subsequently, knockdown or overexpression of candidate regulators in HEY1^+^ CMs, followed by assessment of P5CS, ATP5B, and TOMM20 levels, revealed distinct targeting specificity: for instance, Sh-NADK2 primarily inhibited P5CS, whereas Sh-CKMT2 preferentially downregulated ATP5B, with limited cross-interference between the two axes (**Figure 1L**). This indicates that the P5CS-associated redox maintenance pathway and the ATP5B-associated energy supply pathway can be independently regulated. At the functional level, we established targeted gene modulation systems (including sh-ALDH1L2, sh-NADK2, and OE-TXNIP; as well as sh-CKMT2, sh-LIPT1, and OE-MUL1) in *HEY1^+^* iPSC-CMs and assessed their effects under identical OGD/R conditions, with sampling performed at normoxia, the end of OGD, and the end of OGD/R. Regardless of whether the P5CS-associated redox axis was attenuated or the ATP5B-driven energy metabolism axis was inhibited, cells exhibited consistent injury readouts across both OGD and OGD/R phases, including elevated Ca^2+^ load (**Figure 1M**), enhanced mPTP opening (**Figure 1N**), decreased membrane potential (**Figure 1O**), shortened mitochondrial length (**Figure 1P**), and increased cell mortality (**Figure 1Q**), further supported by mitochondrial network fragmentation observed via confocal imaging (**Figure S2E**). To corroborate the molecular mechanism, we performed gene rescue experiments in *HEY1^+^* cardiomyocytes. During OGD, P5CS downregulation induced by NADK2 knockdown or TXNIP overexpression resulted in mitochondrial damage, which was significantly reversed by P5CS restoration (**Figure 1R**). During OGD/R, disruption of upstream signals triggered apoptosis due to the loss of ATP5B-mediated energy protection; notably, ATP5B restoration effectively reduced cleaved Caspase-3 levels and restored mitochondrial integrity (**Figure 1S**). These results suggest that the buffering capacity of HEY1^+^ CMs against OGD and OGD/R stress relies on the integrity of mitochondrial homeostasis maintained by these key regulators.

In summary, these findings reveal the cellular basis for the survival advantage of *HEY1^+^* CMs under OGD/R challenge, which is attributed to the possession of two functionally specialized mitochondrial subpopulations: specifically, P5CS-enriched mitochondria preferentially buffer oxidative stress shock during OGD by regulating NADP⁺/NADPH redox balance; whereas ATP5B-enriched mitochondria orchestrate energy metabolism reconstitution during OGD/R via efficient ATP synthesis. This stage-dependent defense strategy endows *HEY1^+^* CMs with unique resilience to navigate complex pathological environments.

### Engineering, Characterization, and Uptake Optimization of Subtype-Specific Mitochondria-Enriched Exosomes/EVs Guided by ΔΨ_m_-Based Sorting

**Figure 2:**
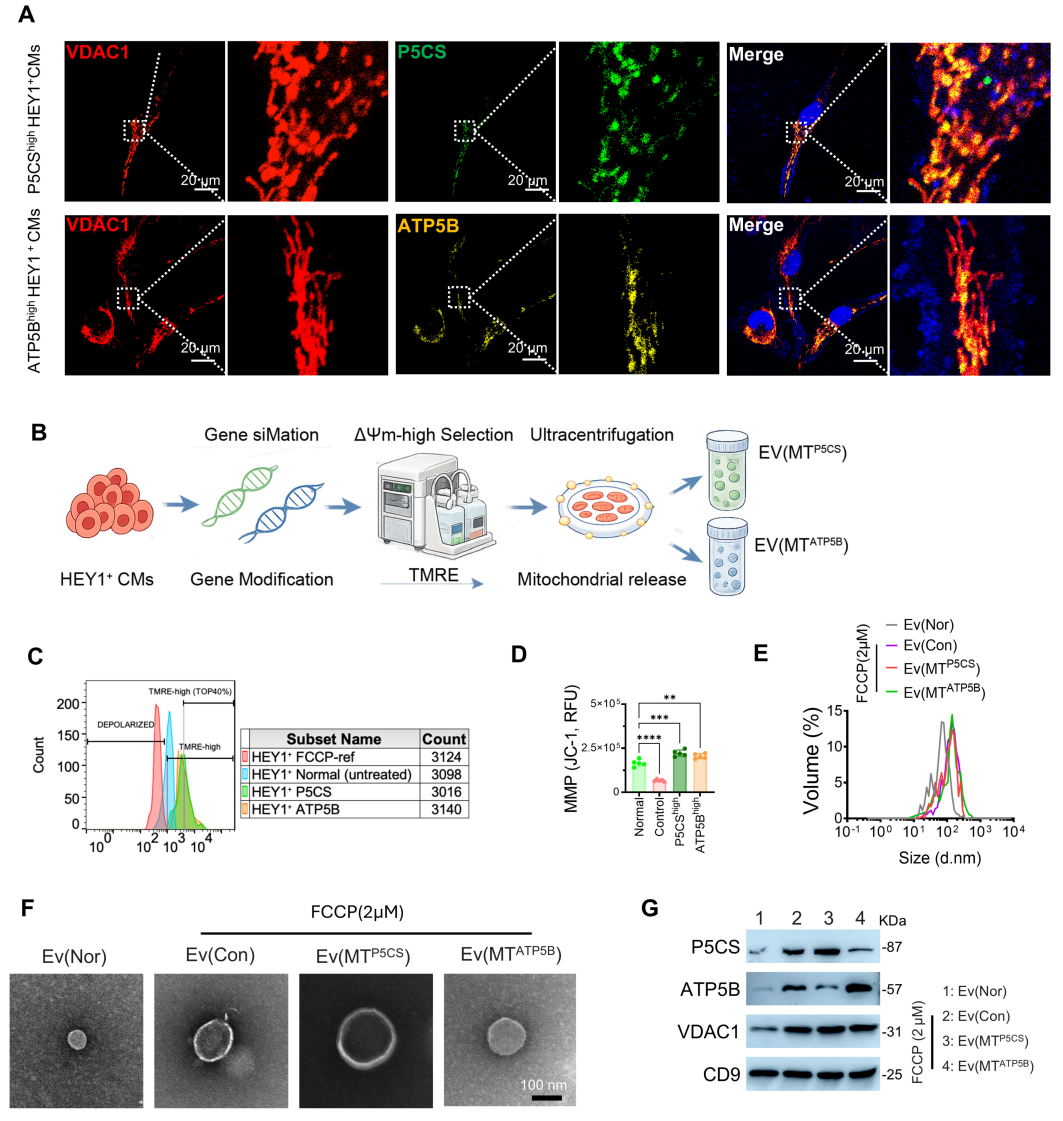
** Standardized preparation and characterization of P5CS/ATP5B-subtype mitochondrial EVs via a ΔΨ_m_-based donor cell engineering strategy. (A)** Confocal imaging of HEY1^+^ iPSC-CMs overexpressing P5CS or ATP5B, immunostained for VDAC1 to visualize the mitochondrial network and intracellular distribution of target proteins. **(B–G)** Source and characterization of mitoEVs. EVs were isolated from HEY1^+^ CMs under normal culture [Ev(Nor)], FCCP-treated HEY1^+^ CMs [Ev(Con)], and ΔΨ_m_-high enriched P5CS-high [Ev(MT^P5CS^)] and ATP5B-high [Ev(MT^ATP5B^)] donor cells.**(B)** Schematic workflow of donor cell processing, ΔΨ_m_-based sorting, and mitoEV isolation. Following genetic modification in HEY1^+^ CMs, cells were loaded with TMRE and subjected to flow cytometric sorting to enrich for ΔΨ_m_-high donor cells, followed by ultracentrifugation to isolate Ev(MT^P5CS^) and Ev(MT^ATP5B^).**(C)** Flow cytometric results of TMRE staining used to identify and enrich ΔΨ_m_-high donor cells within P5CS-high and ATP5B-high populations.**(D)** JC-1 fluorescence assay for assessing the mitochondrial membrane potential (MMP) of donor cells.**(E)** Nanoparticle tracking analysis (NTA) determining the particle size distribution of Ev(Nor), Ev(Con), Ev(MT^P5CS^), and Ev(MT^ATP5B^).**(F)** Transmission electron microscopy (TEM) imaging showing the morphological characteristics of EVs.**(G)** Western blot detection of mitochondrial-related proteins (P5CS, ATP5B, VDAC1) and the exosome marker CD9 in EV samples. **Statistics**: Data are presented as mean ± SD; the independent sample size (*n*) is indicated by data points/labels in the figures. One-way ANOVA with Tukey’s multiple-comparison correction was used for single-factor multi-group comparisons. Two-way ANOVA with Sidak’s or Tukey’s multiple-comparison correction was used for two-factor designs. *P < 0.05, **P < 0.01, ***P < 0.005, ****P < 0.001; *ns*, not significant.

To investigate mitochondrial functional differentiation within HEY1^+^ CMs, we engineered HEY1^+^ CMs with high expression of P5CS (P5CS-high HEY1^+^ CMs) or ATP5B (ATP5B-high HEY1^+^ CMs) and confirmed the high colocalization of target proteins with the mitochondrial outer membrane marker VDAC1, indicating their specific mitochondrial enrichment. This lays the structural foundation for the subsequent preparation of subtype-specific EVs (**Figure 2A**). Given that mitochondrial membrane potential (ΔΨ_m_) reflects respiratory chain function and membrane integrity, we established an integrated preparation workflow combining genetic modification with ΔΨ_m_-based sorting (**Figure 2B**). During donor cell sorting using tetramethylrhodamine ethyl ester (TMRE), we defined the ΔΨ_m_-high population as the Top 40% of TMRE fluorescence intensity, specifically enriching the ΔΨ_m_-high subpopulation from both P5CS-high and ATP5B-high groups (**Figure 2C**). JC-1 staining further confirmed that the sorted ΔΨ_m_-high donor cells maintained highly polarized mitochondrial membrane potential and functional integrity, demonstrating that this strategy effectively enriches a healthy cell population possessing high respiratory chain activity (**Figure 2D**).

Subsequently, we isolated EVs from HEY1^+^ CMs under distinct experimental conditions, including: the normal culture group (Ev(Nor)), the FCCP-stimulated group (Ev(Con)), the P5CS-high group (Ev(MT^P5CS^)), and the ATP5B-high group (Ev(MT^ATP5B^)). FCCP, acting as a mitochondrial uncoupler, significantly induced the release of mitochondrial components into EVs, resulting in increased EV particle size (**Figures 2E, 2F**). Western blot analysis further confirmed that Ev(MT^P5CS^) and Ev(MT^ATP5B^) were enriched with their respective target mitochondrial proteins (P5CS or ATP5B) as well as general EV markers (CD9, VDAC1), demonstrating the successful isolation of functionally specific mitochondrial EV subtypes (**Figure 2G**). Single-particle nanoflow cytometry revealed a distinct segregation of P5CS and ATP5B signals within the MitoTracker-positive EV subpopulation, which predominantly exhibited a single-positive distribution with negligible double-positive or mixed events, indicating high consistency in subtype-specific cargo loading (**Figure S3A**). To determine the optimal endocytic conditions for EVs in recipient cells, we further evaluated the impact of osmotic pressure on the uptake efficiency of iPSC-CMs (**Figure S3B**). Rhodamine tracing experiments revealed that EV uptake by iPSC-CMs exhibited significant osmolarity dependence, peaking at physiological levels (~300 mOsm), whereas deviation from this threshold towards either hypotonic or hypertonic environments significantly inhibited endocytosis (**Figure S3C**). Furthermore, CCK-8 viability assays demonstrated that EV treatment enhanced cardiomyocyte survival following OGD/R injury in a dose-dependent manner, reaching a plateau of ≥80% within the protein concentration range of 5–25 µg/mL (**Figure S3D**). Based on these findings, we established the optimal incubation conditions and effective therapeutic concentrations for subsequent *in vitro* experiments.

In summary, we established a standardized preparation protocol integrating genetic modification with ΔΨ_m_-based sorting, successfully yielding two distinct types of high metabolic activity and functionally specific mitochondrial EVs: Ev(MT^P5CS^) and Ev(MT^ATP5B^). These engineered EVs exhibited superior physicochemical stability, suggesting their potential as robust mitochondrial carriers to mediate myocardial protection via paracrine mechanisms^8^, ^18^. Collectively, based on these physicochemical characterizations and biological validations, we propose an intervention strategy centered on subcellular component engineering, providing a reliable material foundation for the precision treatment of ischemia-reperfusion injury.

### Myocardial Protective Effects of Functionally Heterogeneous Mitochondrial EVs at Distinct Stages of I/R

**Figure 3:**
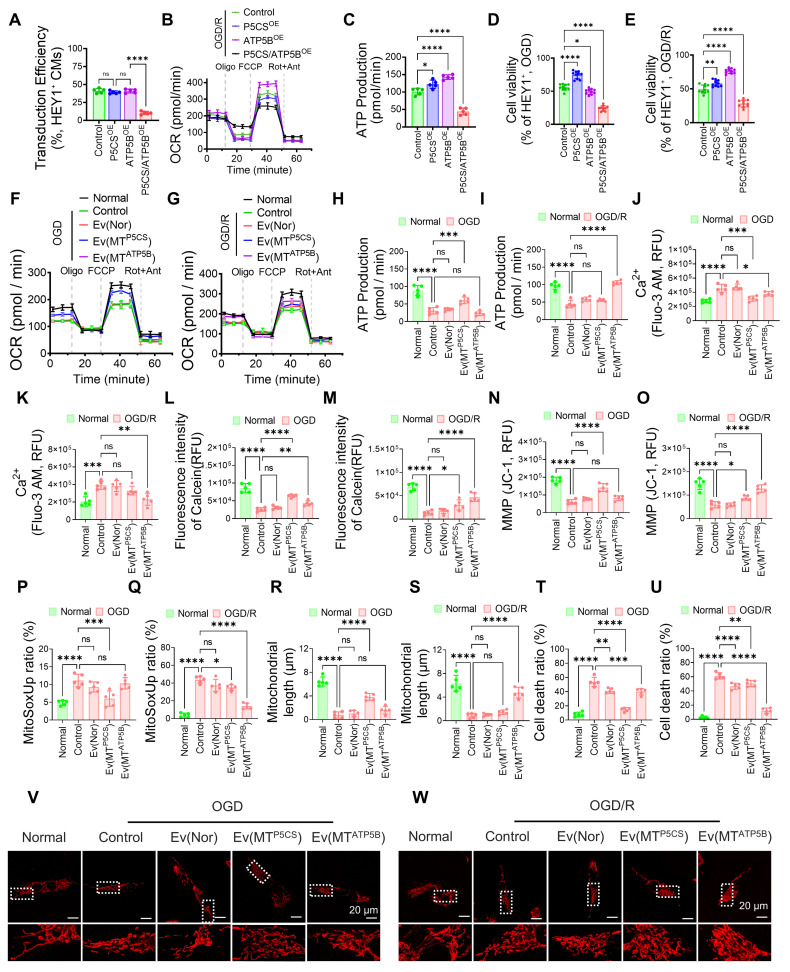
** Differential protective effects and mechanistic verification of functionally heterogeneous mitochondrial EVs during OGD and OGD/R phases. (A)** Quantification of lentiviral transduction efficiency in HEY1^+^ iPSC-CMs across four established groups: Control, P5CS^OE^, ATP5B^OE^, and P5CS/ATP5B^OE^. **(B)** Representative OCR curves from Seahorse XF Mitochondrial Stress Tests performed after OGD/R stress, recording responses to sequential injection of oligomycin (Oligo), FCCP, and rotenone/antimycin A (Rot/Ant) to assess mitochondrial respiration and coupling states. **(C)** Intracellular ATP levels calculated from the Seahorse data in **(B)**. **(D–E)** Assessment of cell viability in different donor cell groups under OGD **(D)** and OGD/R **(E)** conditions, verifying stress tolerance differences between single-axis enhancement and simultaneous dual overexpression during hypoxia and reperfusion phases. **(F–W)** Evaluation of mitochondrial function and cell viability in iPSC-CMs exposed to OGD or OGD/R conditions and treated with Ev(Nor), Ev(MT^P5CS^), or Ev(MT^ATP5B^), respectively. Parameters include: **(F–G)** mitochondrial respiration (OCR) measured after sequential addition of Oligo, FCCP, and Rot/Ant; **(H–I)** intracellular ATP levels; **(J–K)** cytosolic Ca^2+^ concentrations determined using Fluo-3 AM; **(L–M)** mPTP opening status indicated by Calcein fluorescence intensity; **(N–O)** MMP detected via JC-1 fluorescence; **(P–Q)** mitochondrial superoxide generation assessed by MitoSOX staining; **(R–S)** quantitative analysis of mitochondrial length; **(T–U)** cell death detection via Annexin V/PI staining; and **(V–W)** confocal microscopic analysis of mitochondrial morphology. **Statistics**: Data are presented as mean ± SD; the independent sample size (*n*) is indicated by data points/labels in the figures. One-way ANOVA with Tukey’s multiple-comparison correction was used for single-factor multi-group comparisons. Two-way ANOVA with Sidak’s or Tukey’s multiple-comparison correction was used for two-factor designs. *P < 0.05, **P < 0.01, ***P < 0.005, ****P < 0.001; *ns*, not significant.

Following secondary lentiviral transduction in HEY1^+^ iPSC-CMs, we established Control, P5CS-overexpressing (P5CS^OE^), ATP5B-overexpressing (ATP5B^OE^), and concurrent dual-overexpressing (P5CS/ATP5B^OE^) cell populations. Flow cytometry indicated that the transduction efficiency of the dual-overexpression group was significantly lower than that of the single-overexpression groups (**Figure 3A**), suggesting the difficulty in stably establishing and maintaining a "double-high" state. Bioenergetic analysis revealed that dual overexpression failed to yield additive benefits: post-OGD/R ATP production was not superior to that of single-overexpression groups (**Figures 3B–C**), and was accompanied by limited maximal respiratory capacity (**Figure S4B**) and increased proton leakage (**Figures S4A, S4C**). Regarding cellular outcomes, the P5CS/ATP5B^OE^ group exhibited no superiority in cell viability compared to single-axis overexpression groups under either OGD or OGD/R conditions (**Figures 3D–E**). These results indicate that the simultaneous upregulation of both mitochondrial pathways does not confer additive advantages but rather leads to impaired respiratory coupling and increased energy dissipation, thereby limiting the enhancement of stress tolerance. Subsequently, we evaluated the stage-specific protective efficacy of different EV subtypes. During OGD, P5CS-enriched EVs [Ev(MT^P5CS^)] significantly improved mitochondrial respiratory function, enhancing ATP production, basal respiration, and maximal respiratory capacity while reducing proton leak, demonstrating superior efficacy compared to Ev(MT^ATP5B^) and dual-cargo EVs (**Figures 3F, 3H**; **Figures S4D–H**; **Figures S6A–C**). Furthermore, Ev(MT^P5CS^) mitigated hypoxia-induced homeostatic disruptions, characterized by the inhibition of Ca^2+^ overload, reduction of mPTP opening, maintenance of membrane potential, and attenuation of ROS levels (**Figures 3J, 3L, 3N, 3P**; **Figures S4I–L**; **Figure S5A**; **Figure S6G**). Moreover, Ev(MT^P5CS^) was more effective in preserving mitochondrial length and reducing cell death (**Figures 3R, 3T, 3V**; **Figures S4M–N**; **Figure S5C**; **Figure S6I**). In contrast, during OGD/R, ATP5B-enriched EVs [Ev(MT^ATP5B^)] exhibited superior reparative efficacy. It significantly restored mitochondrial respiratory capacity and ATP generation (**Figures 3G, 3I**; **Figures S4O–S**; **Figures S6D–F**), while simultaneously improving Ca^2+^ **recovery**, inhibiting mPTP opening, accelerating membrane potential recovery, and alleviating ROS burden (**Figures 3K, 3M, 3O, 3Q**; **Figures S4T–W**; **Figure S5B**; **Figure S6H**). Further phenotypic evidence confirmed that Ev(MT^ATP5B^) demonstrated optimal protective efficacy during OGD/R, maximally maintaining mitochondrial structure and blocking cell death (**Figures 3S, 3U, 3W**; **Figures S4X–Y**; **Figure S5D**; **Figure S6I**). Conversely, the intervention effects of Ev(MT^P5CS^) and dual-cargo EVs were less pronounced at this stage, validating that protective strategies must be precisely matched to the pathological characteristics of the specific injury stage. Isotope tracing results provided direct metabolic evidence supporting these stage-specific differences. During OGD, ¹³C₅-glutamine tracing revealed that the Ev(MT^P5CS^) group exhibited significantly increased glutamate cycle flux and NADPH/NADP ratio (Figure S5E), suggesting an adaptation to OGD stress via enhanced reductive metabolism. During OGD/R, ¹³C₆-glucose tracing demonstrated that the Ev(MT^ATP5B^) group showed increased carbon flux into the TCA cycle and an elevated ATP/ADP ratio (Figure S5F), confirming its superior capacity to drive the restoration of oxidative phosphorylation during OGD/R. Mechanistically, simultaneous dual overexpression failed to exhibit synergistic effects due to bioenergetic constraints: impaired respiratory coupling exacerbated proton leak-induced energy dissipation, thereby weakening basal cellular tolerance. In contrast, the precision of our strategy stems from the synergistic drive of "membrane potential sorting" and "targeted protein engineering": the sorting step eliminates damaged components, including those that may release pro-apoptotic factors or generate excessive ROS, ensuring that single-axis EVs can repair stage-specific metabolic defects in an optimal state. Specifically, during the ischemic phase where the electron transport chain is inhibited, simply enhancing the ATP5B axis is ineffective and may aggravate oxidative burden^19^; whereas the sorted P5CS subtype preferentially provides antioxidant buffering by maintaining NADP⁺/NADPH homeostasis^20^. Conversely, during the reperfusion phase when the restoration of oxidative phosphorylation dominates, the high-activity ATP5B subtype effectively drives energy resuscitation and physiological reconstruction^21^. In conclusion, intervention with single-advantage subtypes that match dynamic pathological demands offers a more robust protective effect than simple dual-cargo superposition.

### Construction, Characterization, and *In Vivo* Delivery Efficacy of the Metal Ion-Responsive Microneedle System

**Figure 4:**
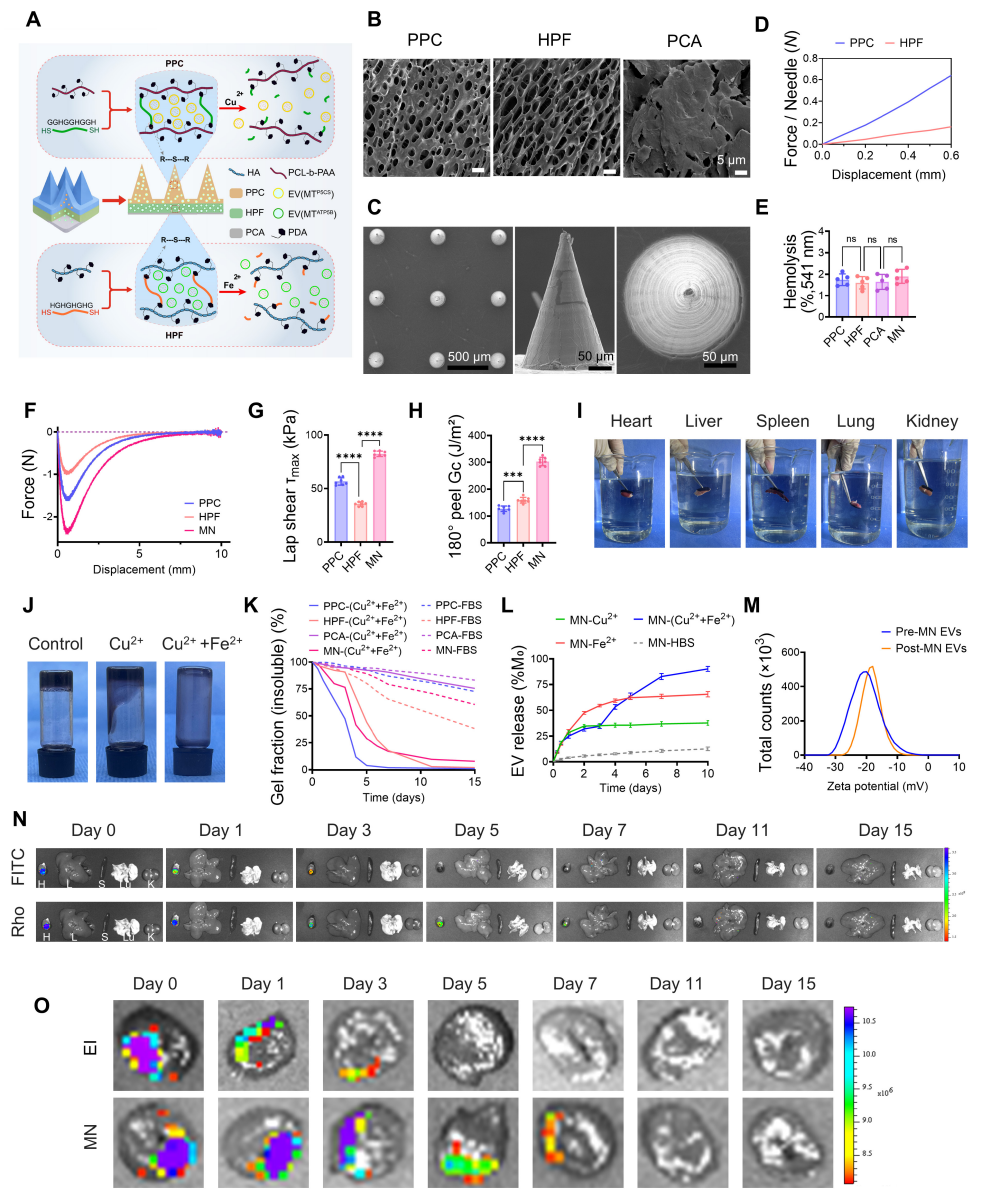
** Construction and performance evaluation of the microneedle (MN) system. (A)** Schematic of the MN patch loaded with two types of exosomes [Ev(MT^P5CS^) and Ev(MT^ATP5B^)]. The system comprises three functional components: a PPC tip (composed of PCL-b-PAA, PDA, and a copper-ion-responsive peptide), an HPF base matrix (composed of HA, PDA, and an iron-ion-responsive peptide), and a PCA anti-adhesion barrier layer (composed of poly(ethylene glycol) diacrylate (PEGDA), carboxylated cellulose nanocrystals, and acrylic acid). **(B)** SEM images showing microstructural differences among PPC, HPF, and PCA. The PPC structure is relatively compact with visible microporosity; HPF exhibits a highly porous and loose network structure; while PCA presents a dense block-like morphology. Scale bar: 5 μm. **(C)** SEM visualization of the MN arrays, highlighting the uniform conical geometry and orderly arrangement of individual microneedles. Scale bars: left, 500 μm; middle/right, 50 μm. **(D)** Single-needle mechanical testing indicating the superior puncture performance of the PPC tip, exhibiting a high load-displacement response that meets the strength requirements for penetrating the epicardium. **(E)** Hemolysis assay showing hemolysis rates below 5% for all material groups (ns), confirming good hemocompatibility. **(F–H)** Quantitative evaluation of adhesion performance via **(F)** force-displacement curves, **(G)** lap shear strength, and **(H)** 180° peeling tests. Results show that the MN patch possesses significantly better adhesion stability on wet myocardial surfaces compared to single materials. **(I)** Ex vivo organ lifting experiments visually demonstrating that the MN patch, after water immersion, remains firmly attached to heart, liver, spleen, lung, and kidney tissues without obvious detachment. **(J–L)** Ion-specific degradation and temporal cascade release kinetics. Validated by **(J)** macroscopic morphological evolution, **(K)** gel degradation rate, and **(L)** exosome cumulative release curves, the system demonstrates hierarchical response characteristics to microenvironmental ions: initial addition of Cu^2+^ **(t = 0)** rapidly triggers PPC layer disintegration to induce early pulsatile release, while subsequent addition of Fe^2+^ at day 3 specifically initiates HPF layer degradation to maintain late-stage sustained release; under this sequential Cu^2+^/Fe^2+^ stimulation mode, a programmed delivery highly matched to the pathological evolution of MI/R is successfully achieved. **(M)** The Zeta potential distribution of exosomes before and after release is highly superimposable, indicating that the MN carrier and release process well preserved the physical integrity of the exosomes without causing significant damage. **(N)** Dual fluorescence tracing of the MN applied to the heart in a rat **MI/R** model: Real-time imaging confirms that under Cu^2+^ and Fe^2+^ stimulation, FITC-loaded PPC (early signal) and Rhodamine-loaded HPF (late signal) release different exosomes respectively, and the release behavior is ion-triggered. **(O)** Ex vivo fluorescence imaging of hearts delivering Rhodamine-labeled exosomes via MN versus epicardial injection (EI) in the rat MI/R model, comparatively evaluating retention time and distribution differences within myocardial tissue. **Statistics**: Data are presented as mean ± SD; the independent sample size (*n*) is indicated by data points/labels in the figures. One-way ANOVA with Tukey’s multiple-comparison correction was used for single-factor multi-group comparisons. Two-way ANOVA with Sidak’s or Tukey’s multiple-comparison correction was used for two-factor designs. *P < 0.05, **P < 0.01, ***P < 0.005, ****P < 0.001; *ns*, not significant.

Targeting the pathological temporal sequence of MI/R, in which Cu²⁺ is elevated during ischemia and Fe²⁺/Fe³⁺ is elevated during reperfusion^22^, ^23^, we constructed an ion-responsive intelligent microneedle system designed to achieve two-stage, precision EV delivery by sensing microenvironmental ion gradients (**Figure 4A**). The system features a multilayer composite structure: the PPC tip utilizes a dense microporous structure to load Ev(MT^P5CS^) for responding to early Cu^2+^ signals, while the HPF matrix accommodates Ev(MT^ATP5B^) within a loose porous network to match late-stage Fe^2+^ release; the PCA backing layer serves as a dense physical barrier (**Figure 4B**). Scanning electron microscopy (SEM) imaging confirmed that the microneedle arrays possess uniform conical geometry and orderly arrangement (**Figure 4C**). Furthermore, the PPC tips exhibited sufficient mechanical strength to penetrate the epicardium (**Figure 4D**), and histological sections verified their effective insertion into the myocardium (**Figure S7N**).To establish the interfacial compatibility of the microneedle (MN) system with the dynamic beating heart, we systematically evaluated its biocompatibility and tissue adhesion performance. The hemolysis rate of all components was below 5% (**Figure 4E**), indicating no obvious toxicity to cardiomyocytes (**Figure S7A**), renal tubular cells, or hepatocytes (**Figures S7B–C**). Meanwhile, the electrical conductivity of PPC and HPF hydrogels was comparable to that of native myocardium, minimizing potential interference with cardiac electrophysiological conduction (**Figure S7D**). Targeting the wet and dynamic beating characteristics of the cardiac surface, we verified the tissue adhesion performance of the MNs through multidimensional mechanical models. Force-displacement curves (**Figure 4F**), lap shear (**Figure 4G**), and 180° peeling (**Figure 4H**) tests consistently demonstrated that, compared to single components, the MN patch established robust connections at wet interfaces. Quantitative analysis confirmed significantly enhanced interfacial adhesion force (**Figures S7E–F**), shear resistance, and interfacial peeling toughness.

Even under cyclic adhesion testing simulating heart beating, the adhesion strength showed no significant decay, exhibiting excellent fatigue resistance (**Figure S7G**). *Ex vivo* organ lifting experiments visually confirmed that the MN patch could overcome gravity to stably suspend wet tissues, including the heart, liver, spleen, lung, and kidney, without detachment, demonstrating superior anchoring capability in wet environments (**Figure 4I**). *In vitro* degradation kinetics revealed the ion-responsive mechanism of the system. Macroscopic morphological observations (**Figure 4J**) and quantitative gel fraction analysis (**Figure 4K**) consistently confirmed that Cu^2+^ and Fe^2+^ specifically triggered the structural disintegration of the PPC and HPF hydrogel layers, respectively. This matrix degradation kinetics was precisely translated into a "two-stage" programmed release profile: Cu^2+^-induced dissociation of the PPC layer drove an early pulsatile release, followed by Fe^2+^-initiated degradation of the HPF layer to maintain a late-stage sustained release, closely matching the pathological temporal requirements of MI/R (**Figure 4L**; **Figure S7H**). Throughout the release process, the physical integrity of the exosomes was well-preserved by the microneedle carrier. Zeta potential (**Figure 4M**) and particle size distribution (**Figure S7I**) before and after release were highly superimposable, with no observed aggregation or disruption. Furthermore, the material system maintained a neutral pH (**Figure S7J**) and volumetric dynamic balance (**Figure S7K**) throughout the degradation process. Combined with high encapsulation efficiency and drug loading capacity (**Figure S7L**), these results **collectively support** the superior physicochemical stability and delivery reliability of the microneedle system. Finally, we validated the *in vivo* delivery efficacy in both rat and Bama minipig models. In the rat MI/R model, dual fluorescence tracing demonstrated that following microneedle implantation, early release of FITC-PPC and late release of Rhodamine-HPF were achieved in response to pathological ion gradient changes (**Figure 4N**). Compared to traditional epicardial injection, microneedle delivery exhibited superior spatial confinement advantages, not only significantly prolonging the retention time of exosomes in the infarcted area (**Figure 4O**), but also effectively blocking escape to non-target organs such as the lungs via the physical shielding of the PCA backing layer (**Figure S7O**). Molecular analysis in the Bama minipig model further confirmed that P5CS protein was significantly enriched in the early postoperative period, whereas ATP5B protein showed sustained accumulation in the late phase (**Figure S7M**).

Compared to traditional epicardial injection, the system's superior spatial confinement and retention capabilities effectively overcome washout effects and reduce off-target risks^24^. Its design precisely matches metabolic demands: releasing Ev(MT^P5CS^) during the ischemic phase to maintain membrane potential and NADPH levels for survival support, followed by the sequential release of Ev(MT^ATP5B^) during the reperfusion phase to optimize electron transport and restore ATP synthesis^8^. This stage-dependent precision intervention strategy holds clear clinical potential, promising to enhance ischemic tolerance and promote energy reconstitution during the PCI perioperative period, thereby improving therapeutic prognosis.

### Myocardial Protection and Electrophysiological Remodeling via the Ion-Responsive Microneedle Delivery System in a C57BL/6 Mouse MI/R Model

**Figure 5:**
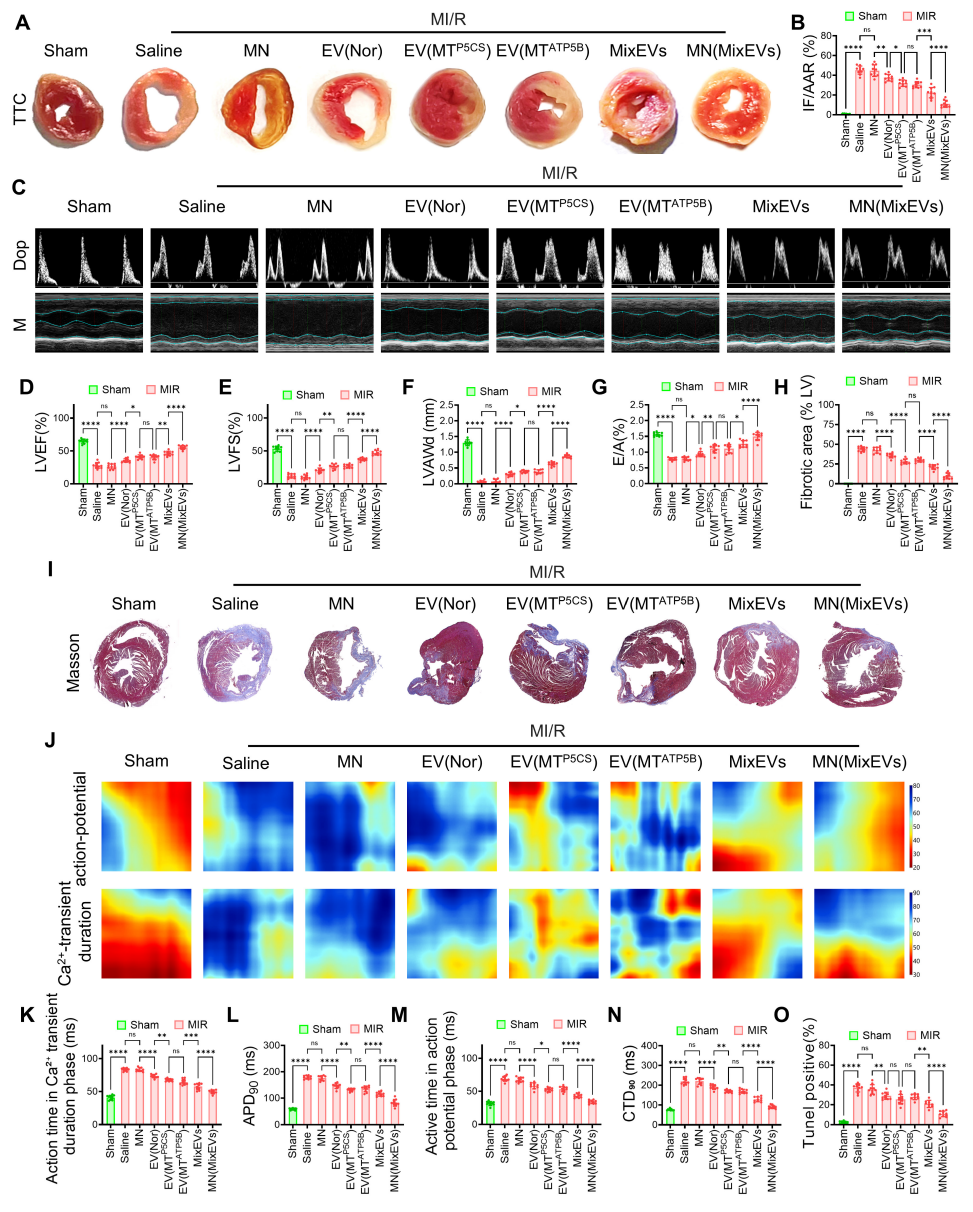
** MN(MixEvs) significantly alleviate myocardial injury, improve cardiac function, and restore electrophysiological homeostasis in MI/R mice. (A)** Representative TTC staining images of cardiac transverse sections, visually displaying the distribution difference between infarcted areas (white) and viable myocardium (red). **(B)** Quantitative analysis showing a significant reduction in the infarct size ratio (IF/AAR) in the MN(MixEvs) group, confirming effective inhibition of acute myocardial necrosis. **(C)** Representative Doppler and M-mode echocardiographic images showing enhanced wall motion amplitude and improved blood flow filling in the MN(MixEvs**)** group. **(D–G)** Quantitative statistics confirming significant improvements in key cardiac function parameters in the MN(MixEvs**)** group: **(D)** Left Ventricular Ejection Fraction (LVEF); **(E)** Left Ventricular Fractional Shortening (LVFS); **(F)** Left Ventricular Anterior Wall thickness at diastole (LVAWd); and **(G)** E/A ratio (diastolic function indicator), indicating comprehensive recovery of systolic and diastolic functions. **(H)** Quantitative results of fibrotic area showing significantly reduced scar formation in the MN(MixEvs) group. **(I)** Masson's trichrome staining tissue sections further confirming reduced collagen deposition (blue) and preservation of healthy myocardium (red) in the treatment group. **(J)** Representative pseudo-color heatmaps of action potentials and calcium transients from optical mapping, visually displaying the restoration of electrical conduction uniformity. **(K–N)** Quantitative statistics of electrophysiological parameters: **(K)** calcium transient rise time; **(L)** action potential duration (APD90); **(M)** activation time; and **(N)** calcium transient duration (**CTD90**). Results suggest concurrent improvements in calcium handling efficiency and repolarization reserve. **(O)** Quantitative results of TUNEL-positive rate confirming that the MN(MixEvs) group significantly inhibited cardiomyocyte apoptosis, constituting the cellular basis for the aforementioned functional improvements. **Statistics**: Data are presented as mean ± SD; the independent sample size (*n*) is indicated by data points/labels in the figures. One-way ANOVA with Tukey’s multiple-comparison correction was used for single-factor multi-group comparisons. Two-way ANOVA with Sidak’s or Tukey’s multiple-comparison correction was used for two-factor designs. *P < 0.05, **P < 0.01, ***P < 0.005, ****P < 0.001; *ns*, not significant.

To evaluate the *in vivo* therapeutic efficacy of the ion-responsive microneedle system, we implanted microneedle patches loaded with mixed exosomes onto the epicardial surface of C57BL/6 mice subjected to MI/R. During the acute injury phase, MN(MixEvs) exhibited significant tissue protective effects. Biochemical analysis revealed that MN(MixEvs) treatment significantly reduced serum levels of CK-MB (**Figure S8A**) and cTnI (**Figure S8B**), indicating alleviated acute myocardial injury. Histopathological evaluation further confirmed a significant reduction in acute-phase infarct size (IF/AAR) as determined by TTC staining (**Figures 5A–B**; **Figure S8C**), demonstrating that this strategy significantly limited the expansion of the myocardial infarction area during the early ischemic period. During the functional recovery phase, MN (MixEvs) significantly improved cardiac function and attenuated pathological remodeling. Echocardiography demonstrated a significant recovery of left ventricular systolic function in the MN(MixEvs) group, evidenced by elevated LVEF and LVFS (**Figures 5C–E**; **Figure S8D**), alongside the effective preservation of LVAWd (**Figure 5F**)). Notably, the increased E/A ratio suggested a concurrent restoration of diastolic function (**Figure 5G**), indicating that the system achieved a comprehensive recovery of both cardiac contractility and compliance. Furthermore, histomorphological assessment revealed that the MN(MixEvs) group significantly reduced the extent of fibrosis (**Figures 5H–I**), demonstrating the system's effectiveness in inhibiting long-term fibrotic scar formation. Electrophysiological assessment revealed that MN(MixEvs) restored cardiac electrical homeostasis and optimized Ca^2+^ cycling. Optical mapping analysis demonstrated that MN(MixEvs) restored the uniformity of electrical conduction (**Figure 5J**). Quantitative analysis indicated that this strategy significantly shortened both the calcium transient rise time and activation time (**Figures 5K, 5M**), and reversed the MI/R-induced prolongation of APD90 and CTD90 (**Figures 5L, 5N**). TUNEL staining and immunofluorescence further confirmed that MN(MixEvs) significantly reduced cardiomyocyte apoptosis in the infarct border zone (**Figures 5H–I**), providing the cellular basis for the observed functional improvements. Systemic safety assessment revealed that the major organs (heart, liver, spleen, lung, kidney) in the treatment group maintained histological integrity without apparent toxic injury (**Figure S8F**), confirming the favorable *in vivo* biocompatibility of the ion-responsive microneedle system.

Collectively, these mouse experiments demonstrated that MN(MixEvs) achieved concurrent improvements in cardiac structure, contractile function, and electrophysiological homeostasis by mitigating oxidative stress during early ischemia and promoting metabolic energy recovery during reperfusion. However, given the substantial differences between rodents and humans regarding heart size, heart rate, and metabolic rate, we further validated the therapeutic efficacy and translational feasibility in a Bama minipig model, which shares closer anatomical and physiological characteristics with humans^25^.

### Myocardial Protection by Functional Mitochondrial Exosome-Encapsulated Microneedles in a Bama Minipig MI/R Model

**Figure 6:**
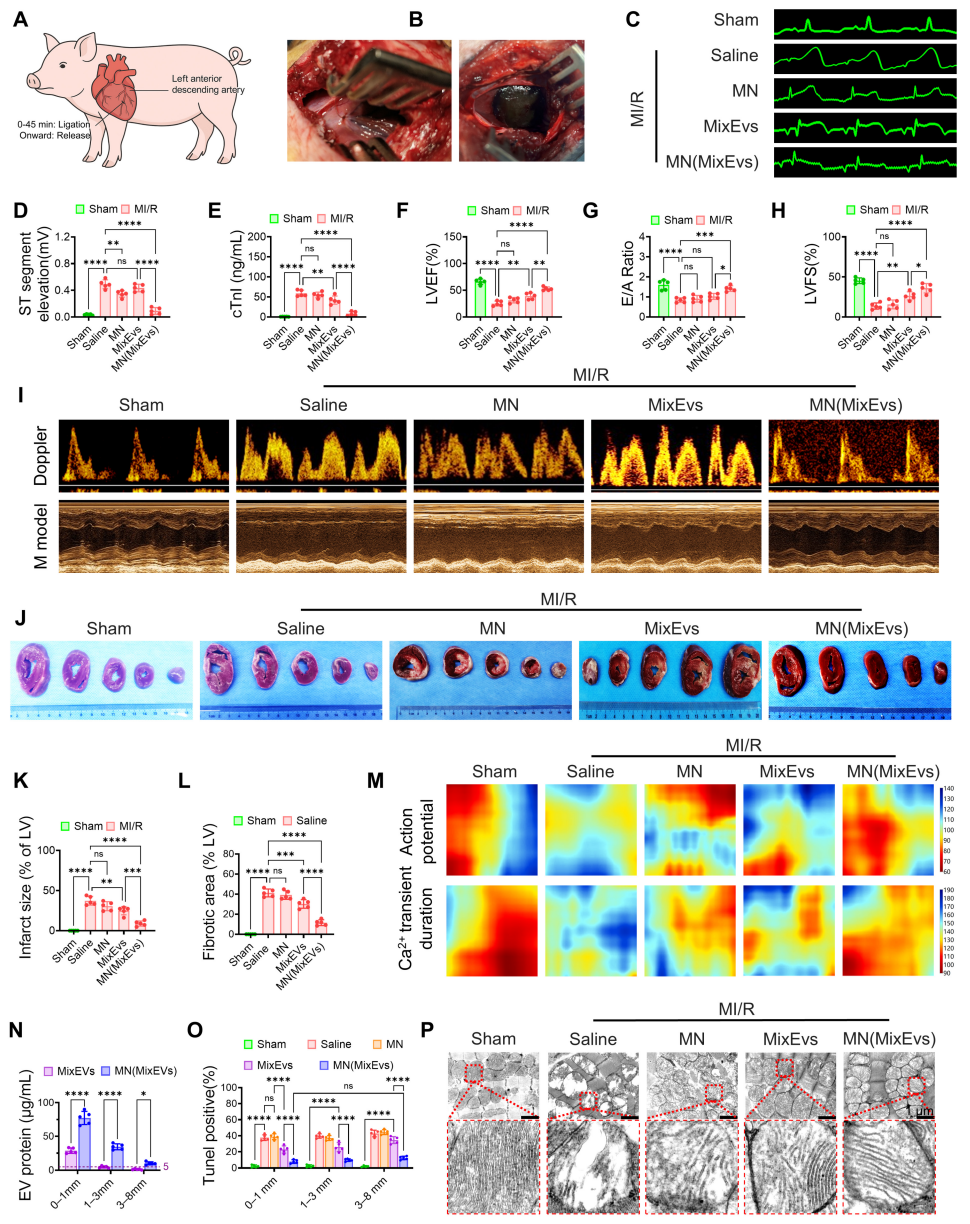
**MN(MixEvs) enhances cardiac function and tissue repair in a Bama minipig model of MI/R. (A)** Schematic illustration of the experimental design for establishing the MI/R injury model in Bama minipigs. **(B)** Intraoperative photographs depicting the localization and fixation of MN(MixEVs) patches on the LV surface. The MN(MixEVs) patch is loaded with a combination of Ev(MT^ATP5B^) and Ev(MT^P5CS^) for localized myocardial delivery. Control groups include: free vesicle injection (a 2:1 ratio mixture of Ev(MT^ATP5B^) and Ev(MT^P5CS^)) and blank MN patches without EVs. **(C)** ECG recordings for each group on post-operative day 1. **(D)** The MN(MixEvs) group exhibited a significant reduction in S-T segment elevation, suggesting restoration of cardiac electrophysiological function. **(E)** Serum cTnI levels at 24 h post-operation were significantly decreased in the MN(MixEvs) group, indicating alleviated acute myocardial injury. **(F–I)** Echocardiographic assessment of cardiac function 4 weeks post-operation via Doppler and M-mode. The MN(MixEvs) group showed significant improvements in key functional parameters: **(F)** LVEF; **(G)** E/A ratio (diastolic function indicator); and **(H)** LVFS. **(I)** Representative echocardiographic images confirming superior recovery of systolic and diastolic functions in the MN(MixEvs) group compared to controls. **(J–L)** Histopathological evaluation of cardiac tissue 4 weeks post-operation. **(J)** Representative TTC-stained cardiac transverse sections showing the distribution of infarct (white) and viable myocardium (red). **(K)** Quantitative analysis demonstrating a significant reduction in infarct size in the MN(MixEvs) group. **(L)** Quantitative results of the fibrotic area, demonstrating significantly reduced scar formation in the MN(MixEvs) group. **(M)** Representative pseudo-color heatmaps of optical mapping for action potentials and calcium transients, showing more uniform electrical conduction and calcium wave propagation in the MN(MixEvs) group, suggesting the restoration of electrophysiological homeostasis. **(N)** Stratified quantification of EVs in the Bama minipig heart 24 h post-MI/R. **(O)** Quantitative analysis of TUNEL-positive rates in stratified tissues (0–1 mm, 1–3 mm, and 3–8 mm). The MN(MixEvs) group exhibited significantly reduced apoptosis at all depths, particularly in the deep myocardium (3–8 mm), demonstrating superior transmural tissue protective effects. **(P)** Transmission electron microscopy (TEM) images showing mitochondrial ultrastructure in myocardial tissue. Compared to the severe fragmentation and cristae disorganization in the **MI/R** group, mitochondrial morphology in the MN(MixEvs) group was well-recovered, with a more regular and intact structural arrangement. Scale bar: 1 μm. Data are presented as mean ± SD; n values are indicated as data points or in figure legends. **Statistics**: Data are presented as mean ± SD; the independent sample size (*n*) is indicated by data points/labels in the figures. One-way ANOVA with Tukey’s multiple-comparison correction was used for single-factor multi-group comparisons. Two-way ANOVA with Sidak’s or Tukey’s multiple-comparison correction was used for two-factor designs. *P < 0.05, **P < 0.01, ***P < 0.005, ****P < 0.001; *ns*, not significant.

To validate the stage-specific protective efficacy of functional mitochondrial exosomes in a clinically relevant large animal model, we established an MI/R model in Bama minipigs and securely implanted the MN(MixEvs) into the myocardium during the early ischemic phase (**Figures 6A–B**). During the acute ischemic injury phase, MN(MixEvs) significantly attenuated myocardial damage. P5CS, a key enzyme in the arginine metabolic pathway, promotes glutamine metabolism, maintains mitochondrial homeostasis, buffers energy fluctuations, and enhances antioxidant defenses^20^. Following MN(MixEvs) treatment, electrocardiography (ECG) showed significant mitigation of S-T segment elevation (**Figures 6C–D**), along with a marked reduction in serum cTnI and CK-MB levels (**Figure 6E**; **Figure S9A**), suggesting that the treatment effectively alleviated ischemia-induced acute cardiomyocyte injury by modulating redox states and metabolic pathways. During the reperfusion recovery phase, Ev(MT^ATP5B^) orchestrated functional repair. As the β subunit of mitochondrial ATP synthase and a core component of the oxidative phosphorylation machinery, ATP5B is essential for restoring cellular ATP levels following reperfusion^21^. Four weeks post-operation, echocardiography demonstrated a significant recovery in LVEF and LVFS in the MN(MixEvs) group, accompanied by a marked reduction in LVIDd**/s** (**Figures 6F–I**; **Figure S9B**; **Figure S10A**). Histopathological evaluation further confirmed that both the infarct size and fibrotic scar area were significantly reduced in the MN(MixEvs) group (**Figures 6J–L**; **Figure S9H**), indicating the effective mitigation of pathological ventricular remodeling. Electrophysiological assessment revealed that MN(MixEvs) restored action potential conduction and optimized calcium handling capabilities, evidenced by the significant shortening of APD90 and CTD90 (**Figure 6M**; **Figures S9C–F**). These findings suggest a reduced risk of arrhythmia, a protective effect likely attributable to the enhanced membrane potential stability resulting from the restoration of mitochondrial energy metabolism^26^. Layer-by-layer quantitative analysis confirmed the deep-tissue delivery advantages of the microneedle system. The MN(MixEvs) group maintained a high concentration distribution of exosome proteins from the superficial (0–1 mm) to deep myocardial layers (**Figure 6N**).

Notably, the local retention concentration in deep regions was stably maintained at >5 µg/mL, remaining above the effective cytoprotective threshold determined *in vitro* (**Figure S3D**). Correspondingly, stratified TUNEL staining confirmed that, in contrast to the marked attenuation of anti-apoptotic efficacy observed in the deep layers of the MixEvs injection group, the MN(MixEvs) group consistently maintained extremely low apoptosis rates throughout the transmural myocardium, particularly in the deep layers (**Figure 6O**; **Figure S9I**). This strongly validates that the microneedle delivery strategy overcomes tissue diffusion limitations, achieving effective transmural protection in the thick-walled myocardium of large animals. Transmission electron microscopy (TEM) observations confirmed that mitochondrial ultrastructure was markedly improved in the MN(MixEvs) group, with substantially reduced fragmentation and more orderly cristae organization compared with the MI/R group, suggesting protection of mitochondrial integrity at the subcellular level (**Figure 6P**; **Figure S9G**). Finally, systemic safety assessment revealed that serum biochemical markers assessed at 24 hours post-operation (UA, γ-GT, UREA, AST) and over the full 28-day cycle (ALT, BUN, CREA, TBIL) all remained within normal physiological ranges (**Figures S10B–I**). Furthermore, H&E staining of major organs (heart, liver, spleen, lung, kidney) showed no pathological damage or inflammatory infiltration (**Figure S10J**), confirming the favorable biocompatibility of the platform.

In summary, this study provides the first evidence in a large animal model that exosome-delivered Ev(MT^P5CS^) and Ev(MT^ATP5B^) exert stage-specific, complementary cardioprotective effects during the ischemic and reperfusion phases of MI/R. P5CS-enriched mitochondria alleviate oxidative stress and metabolic disorders during ischemia, whereas ATP5B-enriched mitochondria promote energy recovery and electrophysiological stability during reperfusion. This stage-specific mitochondrial intervention strategy offers a novel mechanistic basis and holds significant translational potential for MI/R therapy.

### MN(MixEvs) confers systemic protection by maintaining mitochondrial homeostasis and modulating key stress signaling pathways

**Figure 7:**
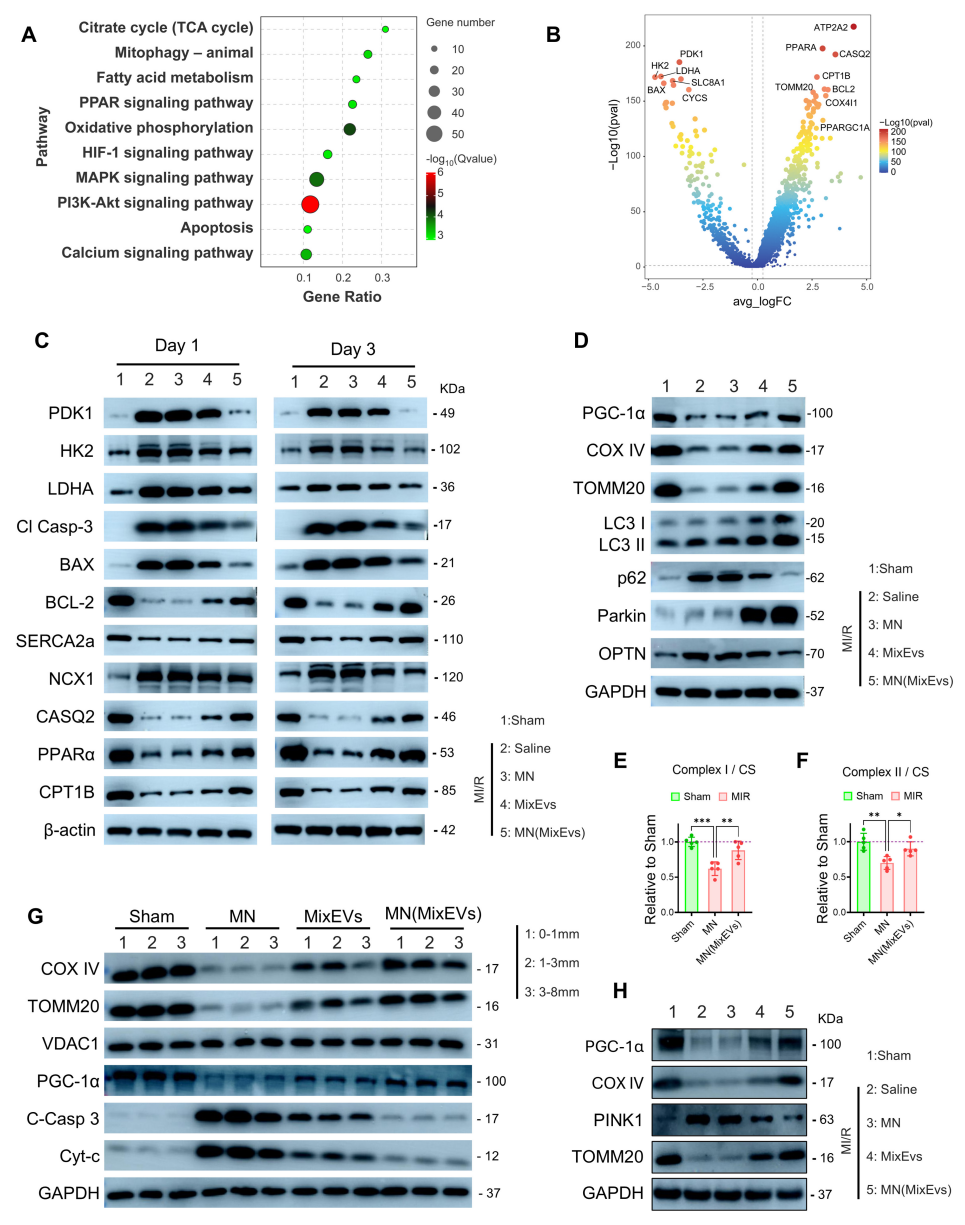
** Multidimensional repair mechanisms of MN(MixEvs) based on mitochondrial bioenergetics, mitophagic flux, and intracellular metabolic networks. (A)** KEGG pathway enrichment bubble plot showing that MN(MixEvs) treatment significantly modulated key pathways, including oxidative phosphorylation, fatty acid metabolism, HIF-1 signaling, calcium signaling, and apoptosis. **(B)** Volcano plot of differentially expressed genes (DEGs) revealing MN(MixEvs)-driven metabolic reprogramming: significant upregulation of mitochondrial oxidative phosphorylation and fatty acid metabolism gene clusters, with concurrent suppression of hypoxic stress (HIF-1) and pro-apoptotic signaling. **(C)** Acute phase (Day 1) metabolic stress and cell death signaling. Western blot analysis showed that the Saline group exhibited elevated stress-induced glycolysis (increased HK2, PDK1, and LDHA) alongside apoptotic activation (increased Cleaved Caspase-3 and BAX; decreased BCL-2). Following MN(MixEvs) treatment, these markers largely returned toward Sham levels, suggesting that microneedle therapy alleviates the ischemia-induced metabolic burden and suppresses cell death. Recovery phase (Day 3) calcium homeostasis and fatty acid metabolism reconstruction. The MN(MixEvs) group significantly reversed the MI/R-induced downregulation of calcium-handling proteins (SERCA2a, NCX1, CASQ2) and key FAO factors (PPARα, CPT1B), indicating the concurrent restoration of calcium homeostasis and metabolic function. **(D)** Western blot analysis of the Bama minipig infarct border zone at 24 h post-operation. The MN(MixEvs) group facilitated the degradation of autophagic receptors (p62, OPTN) and the recruitment of Parkin to enhance mitophagic flux, while maintaining the expression of PGC-1α and TOMM20 to preserve mitochondrial integrity. **(E–F)** Specific activities of key mitochondrial respiratory chain complexes. Analysis of myocardial homogenates from the AAR border zone, normalized to citrate synthase (CS) activity, showed that MN(MixEvs) significantly restored the specific activities of Complex I and Complex II (expressed relative to Sham = 1.0), confirming a substantial restoration of mitochondrial bioenergetic function. **(G)** Validation of therapeutic efficacy in deep tissues at 24 h post-operation. Stratified Western blot analysis (0–1 mm, 1–3 mm, 3–8 mm) showed that MN(MixEvs) significantly inhibited cytochrome c leakage and Caspase-3 activation even within the 3–8 mm deep myocardial layers, confirming that the microneedle system overcomes the diffusion limitations of epicardial injection (MixEvs) to achieve transmural ventricular wall protection. **(H)** Maintenance of mitochondrial homeostasis at 28 days post-operation. Protein analysis showed restored expression of PGC-1α, TOMM20, and COX IV, with PINK1 maintained, suggesting long-term biogenesis and homeostatic remodeling. **Statistics**: Data are presented as mean ± SD; the independent sample size (*n*) is indicated by data points/labels in the figures. One-way ANOVA with Tukey’s multiple-comparison correction was used for single-factor multi-group comparisons. Two-way ANOVA with Sidak’s or Tukey’s multiple-comparison correction was used for two-factor designs. *P < 0.05, **P < 0.01, ***P < 0.005, ****P < 0.001; *ns*, not significant.

To elucidate the systemic protective mechanisms of MN(MixEvs), we initially performed transcriptomic sequencing on the infarct border zone. KEGG and GSEA analyses revealed that MN(MixEvs) significantly upregulated energy metabolism pathways, including oxidative phosphorylation, the TCA cycle, and fatty acid metabolism, while concurrently activating mitophagy and PPAR signaling, suggesting that this strategy facilitates the clearance of impaired mitochondria and the restoration of lipid metabolic homeostasis. In contrast, stress-related pathways, such as HIF-1, calcium signaling, and apoptosis, were globally suppressed (**Figure 7A**; **Figure S11A**). Notably, during the reperfusion phase, the stabilization of mitochondrial membrane potential and the attenuation of ROS levels play a pivotal role in inhibiting cardiomyocyte apoptosis^27^. Further volcano plot analysis elucidated a fundamental phenotypic transition from pathological glycolysis to physiological oxidative metabolism. Specifically, MN(MixEvs) significantly upregulated hub gene clusters associated with mitochondrial respiration, biogenesis, and fatty acid oxidation (FAO), while potently suppressing glycolytic flux, hypoxic stress responses, and pro-apoptotic signaling (**Figure 7B**). This distinct transcriptomic signature confirms that the strategy successfully reconfigured the energy metabolism network, thereby reversing the ischemia-induced state of metabolic suppression. To validate these transcriptomic predictions, we utilized time-course Western blot analysis to examine protein-level metabolic remodeling. During the acute phase (Day 1), MN(MixEvs) significantly suppressed markers of stress-induced glycolysis (PDK1, HK2, and LDHA) and pro-apoptotic signaling (Cleaved Caspase-3 and BAX), while concurrently upregulating the anti-apoptotic protein BCL-2. In the subsequent recovery phase (Day 3), the treatment markedly promoted the upregulation of calcium-handling proteins (SERCA2a, NCX1, and CASQ2) and FAO enzymes (PPARα and CPT1B) (**Figure 7C**). This dynamic transition from acute stress alleviation to the reconstruction of metabolic homeostasis validates the systemic repair mechanisms revealed by the transcriptomic data at the protein level. To elucidate the underlying mechanisms for the sustained maintenance of the metabolic network, we evaluated the dynamics of mitochondrial quality control. MN(MixEvs) facilitated the mitochondrial recruitment of Parkin, which was accompanied by the downregulation of autophagy receptors p62 and OPTN (**Figure 7D**). Lysosomal inhibition assays further confirmed that MixEvs significantly enhanced LC3-II and substrate turnover, confirming a robust enhancement of mitophagic flux (**Figure S11B**). In line with these findings, enzymatic activity assays at 24 h post-operation demonstrated that the activities of respiratory chain complexes I/II (**Figures 7E–F**), complex IV, and FAO (**Figures S11C–D**) were significantly preserved, confirming a substantial restoration of mitochondrial bioenergetic function. Furthermore, we comprehensively evaluated the tissue repair efficacy of MN(MixEvs) across two dimensions: spatial distribution and temporal persistence. Spatially, MN(MixEvs) successfully circumvented physical diffusion barriers. Stratified Western blot analysis revealed that even within the deep myocardial layers (3–8 mm), MN(MixEvs) treatment significantly preserved the expression of mitochondrial structural proteins, such as COX IV and TOMM20, while simultaneously attenuating cytochrome *c* leakage and Caspase-3 activation (**Figure 7G**). Regarding temporal persistence, the protective effects remained sustained over the long term; at 28 days post-operation, the expression levels of PGC-1α, COX IV, TOMM20, and PINK1 exhibited concurrent recovery. This suggests that stable, long-term homeostasis of mitochondrial biogenesis and quality control mechanisms had been established (**Figure 7H**). Collectively, these findings demonstrate that MN(MixEvs) significantly enhances systemic myocardial tolerance to MI/R stress by overcoming spatial physical barriers and establishing enduring therapeutic mechanisms^27^.

In summary, MN(MixEvs) establishes a multidimensional protective mechanism by preserving mitochondrial structural integrity, restoring energy metabolic networks, enhancing autophagic flux efficiency, and reprogramming key signaling pathways. Given that EVs intrinsically harbor a complex repertoire of bioactive cargo, the observed panoramic transcriptomic landscape suggests that the protective efficacy of MN(MixEvs) extends beyond mere physical mitochondrial transplantation. Rather, it likely results from the synergy between exogenous mitochondrial bioenergetics and the intrinsic bioactivity of the exosomal carriers, which collectively endow cardiomyocytes with systemic resilience against MI/R injury.

## Conclusion

Investigations into mitochondrial heterogeneity initially relied on physical separation but have recently evolved to include diverse techniques for characterizing subpopulations at molecular and functional levels. Common strategies currently include fluorescence-activated organelle sorting (**FAMS**/MitoFACS) for high-throughput analysis, immunoaffinity purification (e.g., MitoTag, MITO-IP) targeting outer membrane proteins (e.g., TOMM22, OMP25), and mitochondrial membrane potential (ΔΨ_m_) assays to reflect respiratory coupling and metabolic activity^28^-^30^. While these methods provide effective tools for isolation and assessment, most research remains focused exclusively on mitochondrial-level separation, often overlooking the decisive role of the donor cell's metabolic state in determining mitochondrial quality.

To address this bottleneck, this study established a donor cell engineering strategy combining genetic modification with membrane potential-based sorting. First, we overexpressed P5CS or ATP5B in HEY1^+^ cardiomyocytes to construct metabolic phenotypes specifically adapted to redox maintenance during ischemia or energy reconstruction during reperfusion, respectively. Second, we implemented a flow cytometry sorting protocol based on ΔΨ_m_ to enrich high-quality donor cells with superior respiratory coupling capacity. Given that dual-gene co-overexpression paradoxically impaired respiratory coupling and increased proton leakage, we established a sequential production route focusing on single-function subtypes rather than simultaneous dual-pathway enhancement.

Based on the dynamic fluctuations of copper and iron ions within the damaged myocardial microenvironment, we designed a metal-ion-responsive, stage-specific release microneedle patch, MN(MixEVs), translating subpopulation insights into an *in vivo* therapy that matches the pathological timeline of MI/R. Unlike conventional cardiac microneedles relying on passive diffusion or single-stimuli responses (e.g., pH or ROS), this system leverages endogenous metal ion fluctuations as a trigger mechanism to achieve the autonomous relay release of anti-stress and pro-energetic cargoes^31^, ^32^. This effectively overcomes the challenges of premature or delayed drug release relative to pathological demands.

At the translational level, studies in a Bama minipig model, which closely mirrors human anatomy and physiology, confirmed that this strategy not only improves cardiac function and reduces infarct size but also significantly enhances electrophysiological stability by restoring mitochondrial and calcium homeostasis. This suggests significant potential in preventing lethal arrhythmias and improving long-term clinical outcomes. Despite this potential, several challenges remain: first, current implantation requires surgery, necessitating the development of minimally invasive delivery strategies (e.g., catheter-based intervention); second, long-term follow-up in large animal models is required to confirm chronic improvements in ventricular remodeling and safety margins; third, given individual variations in ion metabolism, companion diagnostic tools should be developed for precision patient screening; finally, the standardized scale-up, quality control, and long-term immunogenicity of iPSC-derived exosomes require further evaluation.

In summary, this study integrates cellular functional characteristics, bio-responsive materials, and mitochondria-targeted interventions to establish a temporally resolved and targeted cardioprotective system. This strategy provides a new paradigm for the precision treatment of myocardial infarction and demonstrates robust translational potential for combined intervention across different stages of PCI.

## Supplementary Material

Supplementary methods and figures.
