# Supplementary material for: Ion-Responsive Microneedles Delivering Subtype-Specific Mitochondrial Extracellular Vesicles from HEY1⁺ Cardiomyocytes for Cardiac Repair in Bama Minipigs with Myocardial Ischemia–Reperfusion Injury
Source: Theranostics. 2026 Jun 10;16(13):7367–87. doi: 10.7150/thno.123209 (PMC13294972; doi:10.7150/thno.123209)
Supplement: Supplementary file 1 — Supplementary methods and figures. [file thnov16p7367s1.pdf]

Support information

Ion-Responsive Microneedles Delivering Subtype-Specific Mitochondrial Extracellular Vesicles from HEY1$^+$ Cardiomyocytes for Cardiac Repair in Bama Minipigs with Myocardial Ischemia–Reperfusion Injury

Peng Qu[1,2,3,8]†, Jiao Shi[1,2,3,8]†, Xue Li[1,2,3,8]†, Yao Gu[1,2,3,8]†, Jun Liu[4,8], Hongyan Zhang[5], MingZhi Zhou[4,8], Cui Ma[6], Xinghui Li[7,8], Wenjie Tian[4], Qi Liang[1,2,3,8#], Gang Li[4#], Panke Cheng[4,8#]

1. Department of Clinical Laboratory, Affiliated Hospital of North Sichuan Medical College, Nanchong, 637000, China.

2. School of Laboratory Medicine, North Sichuan Medical College, Nanchong, 637007, China.

3. Translational Medicine Research Center, North Sichuan Medical College, Nanchong, 637007, China.

4. Institute of Cardiovascular Diseases & Department of Cardiology, Sichuan Provincial People's Hospital, School of Medicine, University of Electronic Science and Technology of China, Chengdu, 610072, China

5. Department of Anesthesiology, Chengdu Wenjiang District People's Hospital, Chengdu 611130, China

6. Department of Mathematics, Army Medical University, Chongqing, 400038, China.

7. Department of Radiology, Affiliated Hospital of North Sichuan Medical College, Nanchong 637000, China

8. Medical Imaging Key Laboratory of Sichuan Province, Nanchong, 637007, China.

†These authors contributed equally.

* ***Correspondence to:*** **Panke Cheng** (E-mail: chengpk1002@163.com), **Gang Li** (E-mail: ligang8252@qq.com) and **Qi Liang** (Email: liangqineijiang@126.com).

Supplementary Materials and Methods

Single-cell Transcriptome Analysis

All bioinformatics analyses were performed using the CeleLens Cloud platform (https://www.celelenscloud.cn) provided by Singleron Biotechnologies. Publicly available human heart single-cell and single-nucleus RNA sequencing (sc/snRNA-seq) datasets were retrieved from the Human Cell Atlas Data Coordination Platform (ERP123138), the European Genome-phenome Archive (EGAS00001006374), and the cellxgene portal (https://cellxgene.cziscience.com/collections/8191c283-0816-424b-9b61-c3e1d6258a77). These datasets encompassed myocardial tissues from individuals with and without myocardial infarction (MI), facilitating comparative analysis between diseased and normal states.

Following standard quality control (filtering cells with fewer than 200 expressed genes or >10% mitochondrial transcripts), normalization, and dimensionality reduction, unsupervised clustering was performed using the Louvain algorithm, with visualization achieved via Uniform Manifold Approximation and Projection (UMAP). Cell type annotation was conducted based on canonical marker genes for cardiomyocytes, endothelial cells, fibroblasts, and immune cells. The Wilcoxon rank-sum test was utilized to evaluate marker gene expression across clusters, with results presented in dot plots and heatmaps. Infarction-associated changes in cellular composition were evaluated by analyzing the distribution of cell subpopulations between the MI and non-MI groups. Statistical differences in cell type abundance were assessed using the Chi-square test. To explore lineage relationships and cell state transitions, pseudotime analysis was performed using Monocle3. Cellular trajectories were reconstructed based on highly variable genes, with the starting state defined by known biomarkers, thereby delineating the differentiation pathways of cell lineages such as cardiomyocytes and fibroblasts.

To elucidate the metabolic functional specificity of the HEY1^{+} cardiomyocyte subpopulation, differential gene expression analysis was performed between the HEY1^{+} and HEY1^{-} subpopulations using single-cell datasets. Kyoto Encyclopedia of Genes and Genomes (KEGG) pathway enrichment analysis was conducted on significantly differentially expressed genes (DEGs) to prioritize key metabolic modules, including antioxidant stress (e.g., glutathione metabolism and the pentose phosphate pathway) and mitochondrial bioenergetics (e.g., the tricarboxylic acid cycle, and

arginine and proline metabolism). Concurrently, volcano plots were generated to visualize genome-wide expression differences. Based on expression abundance and statistical significance, potential upstream regulators governing the P5CS-associated ischemic adaptation axis and the ATP5B-associated reperfusion recovery axis were identified, providing candidate targets for subsequent mechanistic validation.

Differentiation of hiPSCs into Cardiomyocytes (CMs) and HEY1-Overexpressing CMs

Human induced pluripotent stem cells (hiPSCs) were cultured on Matrigel-coated plates in mTeSR™1 medium and maintained under standard conditions (37 °C, 5% CO_2). Monolayer differentiation was initiated when cells reached approximately 90% confluence. Upon initiation, cells were treated with RPMI 1640 medium supplemented with B-27 without insulin and 6 μM CHIR99021 for 24 h, followed by 5 μM IWP-2 for 48 h to modulate Wnt signaling. Subsequently, the medium was replaced with RPMI 1640 supplemented with B-27 containing insulin, with changes performed every 2–3 d. Spontaneously beating cardiomyocytes were typically observed between days 8 and 10. On day 14, metabolic selection was performed using glucose-free medium supplemented with lactate for 4 d to enrich for cardiomyocytes. The resulting hiPSC-CMs exhibited spontaneous contractility and expressed cardiac-specific markers (e.g., cTnI). For HEY1 overexpression, cardiomyocytes were transduced with a lentiviral vector harboring the HEY1 gene (pLV-EF1a-HEY1-IRES-GFP, MOI=10) on day 10, in the presence of 8 μg/mL polybrene to enhance transduction efficiency. GFP expression was observed 48 h post-transduction. Cells transduced with an empty vector served as controls. Both $HEY1^+$ CMs and control CMs were utilized for downstream experiments between days 15 and 20.

Construction of Metabolic Subtype Models in $HEY1^+$ CMs (P5CS-high, ATP5B-high, and Dual-positive)

To establish metabolic subtype-specific models, a second round of genetic modification was performed on the previously constructed $HEY1^+$ CMs (GFP-positive). $HEY1^+$ CMs were transduced with lentiviral vectors carrying either the P5CS gene with an mCherry tag (pLV-EF1α-P5CS-IRES-mCherry) or the ATP5B gene with an mTagBFP2 tag (pLV-EF1α-ATP5B-IRES-mTagBFP2) (**MOI=10**, 8 μg/mL polybrene). 48 h post-transduction, the expression of mCherry and

mTagBFP2 was confirmed via fluorescence microscopy. The population exhibiting double positivity for GFP and mCherry was designated as P5CS-overexpressing (P5CS^{OE}) HEY1$^+$ CMs, while the population double-positive for GFP and mTagBFP2 was designated as ATP5B-overexpressing (ATP5B^{OE}) HEY1$^+$ CMs. Concurrently, to construct P5CS/ATP5B double-positive cells, a single dual-expression lentiviral vector (pLV-EF1α-P5CS-P2A-ATP5B-P2A-mCherry) was utilized to co-overexpress P5CS and ATP5B under identical transduction conditions. After 48 h, P5CS/ATP5B dual-overexpressing HEY1$^+$ CMs (Dual^{OE}) were selected based on GFP and mCherry double positivity. A group transduced with an empty mCherry vector served as a control to rule out vector-associated effects.

Independent Sorting of P5CS and ATP5B Subtype-Specific HEY1+ CMs Based on Mitochondrial Membrane Potential

To individually isolate P5CS or ATP5B single-subtype cell populations with high mitochondrial membrane potential ($\Delta\Psi_m$-high), lentivirally transduced P5CS-mCherry$^+$ or ATP5B-mTagBFP2$^+$ HEY1$^+$ iPSC-CMs were prepared as single-cell suspensions by gentle digestion with low-concentration collagenase type II (0.5 mg/mL; Gibco) at 37 °C. Cells were then incubated with 50 nM tetramethylrhodamine ethyl ester (TMRE; Thermo Fisher Scientific) in culture medium at 37 °C for 30 min in the dark. After washing and resuspension in PBS, independent sterile sorting procedures were performed using a BD FACSAria Fusion cell sorter. The gating strategy first excluded debris based on FSC/SSC and identified single cells using FSC-A/FSC-H. Subsequently, the mCherry$^+$ population was gated for the P5CS group, and the mTagBFP2$^+$ population was gated for the ATP5B group. Within these target populations, the top 40% of cells exhibiting the highest TMRE fluorescence intensity were defined as the high-potential subgroups. Finally, the sorted mCherry$^+$/$\Delta\Psi_m$-high cells (designated as P5CS-high HEY1$^+$ CMs) and mTagBFP2$^+$/$\Delta\Psi_m$-high cells (designated as ATP5B-high HEY1$^+$ CMs) were collected into medium containing 20% FBS for subsequent expansion. The empty vector control group was processed in parallel under identical conditions.

Immunofluorescence Assay

Cells were seeded onto glass coverslips and cultured to an appropriate density. Subsequently, cells were fixed with 4% paraformaldehyde (PFA) for 15 min at room temperature. After washing with PBS, cells were permeabilized with 0.2% Triton X-100 for 15 min and blocked with 5% bovine serum albumin (BSA) for 30 min at room temperature to prevent non-specific binding. Following the removal of the blocking solution, samples were incubated overnight at 4 °C with the following primary antibodies: anti-P5CS (Abcam), anti-ATP5B (Proteintech), and the mitochondrial outer membrane marker anti-VDAC1 (Proteintech). On the following day, cells were washed three times with PBS and incubated with Alexa Fluor-conjugated secondary antibodies for 1 h at room temperature in the dark. Nuclei were counterstained with Hoechst. After mounting, images were acquired using a confocal laser scanning microscope.

Construction of a Sequential Gene Modification and Metabolic Rescue Model in HEY1$^+$ CMs

To dissect the HEY1-driven downstream metabolic regulatory network within terminally differentiated cardiomyocytes, a "Perturbation-Rescue" Sequential Transduction Strategy was employed to establish a functional validation model. Initially, on day 10 of differentiation, iPSC-CMs were transduced with a lentiviral vector harboring the HEY1 gene (MOI=10, with 8 μg/mL polybrene). The HEY1$^+$ cardiomyocyte phenotype was confirmed 48 h later based on GFP expression. Subsequently, on day 12, the first round of gene perturbation was initiated by introducing shRNA lentiviruses targeting ALDH1L2, NADK2, CKMT2, and LIPT1 (vector: pLKO.1-TRC) or overexpression lentiviruses for TXNIP and MUL1 (vector: pLV-EF1α-IRES-Puro) at an MOI=20. Scrambled shRNA (Scramble) and Empty Vector controls were included as negative controls. To verify the causal rescue effects of P5CS and ATP5B, a second round of rescue transduction was performed 24 h after the first-round transduction (day 13). This involved the introduction of pLV-EF1α-P5CS-IRES-mCherry or pLV-EF1α-ATP5B-IRES-mTagBFP2 viruses (MOI=20), alongside corresponding fluorescent empty vector controls. Following transduction, cells underwent glucose-free/lactate metabolic purification from days 14 to 18 to further enrich for cardiomyocytes. On day 16 (96 h post-perturbation and 72 h post-rescue), Western blot analysis confirmed knockdown efficiency (>70%) and the significant restoration of rescue proteins, verifying that all groups achieved the expected expression levels. Upon completion of purification, cells were equilibrated in standard glucose-containing medium for ≥12–24 h. Oxygen-glucose

deprivation/reoxygenation (OGD/R) modeling was subsequently performed on days 19–20. Mitochondrial morphological integrity and the recovery of respiratory function were evaluated using confocal microscopy and the Seahorse XF Analyzer system, thereby characterizing the reparative efficacy of specific metabolic axes within a high-purity cardiomyocyte background.

Isolation and Characterization of Extracellular Vesicles (EVs)

Cardiomyocyte-derived EVs were harvested after culturing cells in medium supplemented with exosome-depleted fetal bovine serum (Gibco) for 48 h. Four groups of EV samples were prepared: Ev(Nor) from untreated HEY1$^+$ CMs; Ev(Con) from HEY1$^+$ CMs treated with FCCP (2 μM, 4 h); and Ev(MT^{P5CS}) and Ev(MT^{ATP5B}) derived from FCCP-treated P5CS-high HEY1$^+$ CMs and ATP5B-high HEY1$^+$ CMs, respectively. Conditioned medium was collected and subjected to sequential centrifugation (300 × g for 10 min; 2000 × g for 20 min) to remove cells and debris. The supernatant was filtered through a 0.22 μm membrane, followed by ultracentrifugation at 100,000 × g for 70 min at 4 °C. The resulting pellets were washed with PBS, ultracentrifuged again under the same conditions, finally resuspended in PBS, and stored at −80 °C. Particle size and concentration were determined via nanoparticle tracking analysis (NTA). For morphological assessment using transmission electron microscopy (TEM), samples were loaded onto copper grids, negatively stained with 2% uranyl acetate, air-dried, and imaged. Protein concentration was quantified using the BCA assay (Thermo Fisher). For Western blot analysis, equal amounts of protein were separated by SDS-PAGE and transferred to PVDF membranes. Membranes were probed with primary antibodies (Proteintech), including anti-P5CS (to assess mitochondrial P5CS enrichment), anti-ATP5B (to detect the ATP synthase subunit), anti-VDAC1 (as a total mitochondrial loading control), and anti-CD9 (as an EV surface marker and internal control). Detection was visualized using HRP-conjugated secondary antibodies and ECL substrates.

Dynamic Light Scattering Characterization of EV Size Distribution and Zeta Potential

To comprehensively evaluate the physicochemical properties of EVs before and after microneedle (MN) loading, two groups of samples were established for comparative analysis. Initially, separately prepared Ev(MT^{P5CS}) and Ev(MT^{ATP5B}) were mixed at a defined ratio to construct the initial total EV pool (Pre-MN EVs). To minimize interference from high-abundance proteins and free salt ions

on scattering signals, all samples underwent a rigorous sequential purification procedure prior to measurement. Samples were sequentially centrifuged at 2,000 × g for 10 min and 10,000 × g for 20 min to remove cell debris and large aggregates, followed by ultracentrifugation at 100,000 × g for 70 min to pellet EVs, which were then resuspended in 0.22 µm-filtered and degassed PBS (pH 7.4). For stability assessment following MN release (Day-5 Post-MN EVs), samples were recovered from the supernatant on day 5 of the release kinetics experiment. To eliminate potential interference from metal ions in the release medium, the collection solution was pre-treated with EDTA (final concentration 2 mM) for chelation, followed by purification using the same ultracentrifugation and resuspension steps described above. Prior to measurement, EV mass concentration was adjusted to 50–100 µg/mL based on Micro-BCA quantification, with dilution in PBS (1:2–1:5) applied when necessary to ensure scattering intensity remained within the optimal linear response range of the instrument. Particle size distribution and Zeta potential were measured using a Malvern Zetasizer Pro (ZS Xplorer software) at a constant temperature of 25 °C. Each independent sample was placed in a disposable low-volume cuvette and equilibrated for 2–3 min to eliminate thermal convection and microbubbles. For particle size measurement, non-invasive backscatter (NIBS, 173°) technology was employed, with attenuator settings automatically optimized by the instrument. Data were acquired from a minimum of 10 consecutive sub-runs, and data quality was monitored in real time by the software's built-in algorithms, which automatically rejected runs affected by dust interference. The intensity-weighted hydrodynamic diameter (Z-average) and polydispersity index (PDI) were reported, and particle size distributions were converted from intensity-based to volume-based representations (Volume %) according to Mie scattering theory. For Zeta potential measurement, samples were transferred to a dedicated electrophoretic cell, and data were acquired from a minimum of 10 sub-runs under standard electric field conditions. Electrophoretic mobility was converted to apparent Zeta potential (mV) based on the Smoluchowski approximation model, and potential distribution profiles were generated. Both Pre-MN and Post-MN groups included five independent biological replicates, and each biological sample was measured in five technical replicates, with averaged values used for subsequent analysis. All measurements were corrected by subtracting the background scattering and potential baseline of the PBS blank control and results are expressed as mean ± SD.

High-Resolution Single-Particle Flow Cytometry Analysis of Mitochondrial Cargo in Engineered Extracellular Vesicles

Purified EVs were serially diluted in 0.1-μm filtered PBS. To strictly exclude swarm effect interference, the correlation between event rate and dilution factor was confirmed by linear regression analysis (R2 > 0.99), and the sample flow rate was consistently maintained below 10,000 events/s. Diluted samples were incubated with 100 nM of the pan-mitochondrial probe MitoTracker Deep Red FM (Thermo Fisher Scientific) at 37 °C for 20 min in the dark. Without washing, samples were directly acquired using a CytoFLEX flow cytometer (Beckman Coulter) equipped with a high-sensitivity violet side scatter (V-SSC, 405 nm) module. Instrument resolution and the analysis gating region (100–1,000 nm) were calibrated using a submicron reference bead mixture (ApogeeMix, Sigma-Aldrich; particle size range 100–900 nm). Data analysis was performed using FlowJo v10.8 software with a hierarchical gating strategy. Briefly, the main vesicle population was first identified based on V-SSC and forward scatter (FSC) characteristics. Mitochondria-positive vesicles (mitoEVs) were then gated based on fluorescence intensity in the APC channel (Ex: 638 nm / Em: 660 nm). Within this parent population, EV subtypes were distinguished according to genetically encoded fluorescent reporters: mCherry-positive vesicles, corresponding to Ev(MT^{P5CS}), were quantified in the PE channel (Ex: 561 nm / Em: 610 nm), while mTagBFP2-positive vesicles, corresponding to Ev(MT^{ATP5B}), were quantified in the PB channel (Ex: 405 nm / Em: 450 nm). To confirm vesicle-specific signals and exclude reagent-derived background, a blank control containing buffer and probe only was included to rule out dye aggregation. In addition, a 0.1% Triton X-100 detergent lysis control was applied, and the disappearance of fluorescence signals following membrane disruption was used to verify the membranous nature of the detected particles.

Synthesis of PPC and PPC_Ev(MT^{P5CS})

To synthesize thiol-terminated polycaprolactone (HS-PCL), 10 mmol of ε-caprolactone (ε-CL, 99% purity) was dissolved in 50 mL of anhydrous toluene. Subsequently, 0.1 mmol of 2-mercaptoethanol was added as the initiator, together with 0.05 mmol of benzoyl peroxide (BPO) as the radical initiator. The reaction was carried out at 120 °C for 12 h under a nitrogen atmosphere. After completion, the reaction mixture was cooled to room temperature and poured into excess cold ethanol to induce precipitation. The resulting product was collected by centrifugation after repeated

ethanol washing to obtain HS-PCL. Next, 1 g of HS-PCL was dissolved in 20 mL of dry N,N-dimethylformamide (DMF), followed by the addition of 10 mmol of acrylic acid (AA, 99% purity) and 0.1 mmol of azobisisobutyronitrile (AIBN) as a thermal initiator. The reaction proceeded at 70 °C for 6 h under nitrogen protection. The mixture was then precipitated in a large volume of cold ether, washed, and vacuum-dried to yield the block copolymer PCL-b-PAA. Successful synthesis was confirmed by 1H NMR analysis using CDCl3 as the solvent. For dopamine grafting, PCL-b-PAA was dissolved in MES buffer (pH 5.5), and EDC and NHS were added at a molar ratio of 1:1 to activate the carboxyl groups on the PAA blocks. After 30 min of reaction at room temperature, dopamine hydrochloride dissolved in PBS (pH 7.2) was slowly added to the activated polymer solution. The reaction was allowed to proceed for 6 h under gentle stirring in the dark to form stable amide bonds. To prevent dopamine oxidation, 0.1 mM ascorbic acid was added during the reaction. Unreacted reagents and byproducts were removed by dialysis to obtain dopamine-functionalized PCL-b-PAA. The degree of catechol substitution (DS_PAA) was determined by UV–Vis/Arnow colorimetric assay and 1H NMR/XPS analysis. To form a dynamic crosslinked structure, 200 μM SH-GGHGGHGGH-SH peptide dissolved in PBS (pH 7.4) was mixed with a 12% (w/v) dopamine-modified PCL-b-PAA precursor solution and incubated for 6 h. During this process, thiol groups on the peptide underwent Michael addition with quinone groups generated from dopamine oxidation, resulting in covalent crosslinking and formation of the PPC hydrogel. Unbound peptides were removed by repeated PBS washing. For the preparation of exosome-loaded PPC (PPC_Ev(MT^{P5CS})), P5CS-mitochondria-enriched exosomes Ev(MT^{P5CS}) suspended in PBS were mixed with the SH-GGHGGHGGH-SH peptide and dopamine-modified PCL-b-PAA precursor solution. During in situ gelation, exosomes were embedded within the forming peptide–dopamine crosslinked network, and the final exosome concentration was adjusted to 2 mg/mL. The resulting hydrogel was obtained either by mild low-temperature drying or maintained in a hydrated state.

Synthesis of HPF and HPF_Ev(MT^{ATP5B})

Sodium hyaluronate (HA, Mw ~100 kDa) was dissolved in MES buffer (pH 5.5, 2 mg/mL) under constant stirring. To activate the carboxyl groups on the HA backbone, EDC and NHS were sequentially added at a molar ratio of 1.2:1. After 15 min of activation, N-(2-aminoethyl) maleimide (AEM) was added at a molar ratio of 1:1 relative to the HA carboxyl groups. The reaction was

allowed to proceed for 4 h at room temperature. During this step, the amino group of AEM reacted with the NHS-activated carboxyl groups on HA to form stable amide bonds, yielding maleimide-functionalized HA (HA-Mal). The resulting solution was dialyzed (MWCO 10 kDa) extensively against deionized water to remove unreacted reagents and byproducts, followed by lyophilization to obtain pale-yellow HA–Mal powder. To introduce catechol functionality, dopamine hydrochloride (dopamine·HCl) was dissolved in PBS (pH 7.4) at a final concentration of 2 mg/mL and slowly added dropwise into the HA-Mal solution (1 mg/mL). The reaction was conducted under light-protected conditions at room temperature for 6 h. The dopamine molecules underwent auto-oxidation of catechol groups to quinones while simultaneously reacting via Michael addition with the maleimide groups on HA-Mal, resulting in covalently grafted dopamine moieties. The final product, dopamine-modified HA (PDA–HA). The degree of catechol substitution DS_{HA} on HA was determined post-reaction using UV–Vis/Arnow assay and ^1H NMR/XPS. For hydrogel crosslinking, SH–HGHGHGHG–SH peptide (200 μM) was dissolved in PBS (pH 7.4) and added to the PDA-HA solution (3 mg/mL). The mixture was incubated at room temperature for 6 h, allowing the thiol groups at both peptide termini to undergo Michael addition with the quinone groups of PDA, forming covalent thioether crosslinks. After crosslinking, unbound peptide was removed by repeated PBS washes, yielding the dynamic and responsive hydrogel structure designated as HPF. To prepare HPF_Ev(MT^{ATP5B}), mitochondrial ATP5B-enriched extracellular vesicles Ev(MT^{ATP5B}) were resuspended in PBS and co-incubated with the SH–HGHGHGHG–SH peptide and PDA-HA precursor solution under mild stirring. During the in situ gelation process, vesicles were physically embedded and retained within the hydrogel matrix. The resulting composite hydrogel was either gently dried or used directly in hydrated form, constituting HPF_Ev(MT^{ATP5B}) for subsequent application as the base and body region of the microneedle patch.

Synthesis of the PCA Hydrogel Barrier Layer

Cellulose nanocrystals (CNCs) were dispersed in deionized water at a concentration of 1 wt% (total volume 200 mL) and sonicated for 30 min to ensure uniform dispersion. To initiate carboxylation, 2,2,6,6-tetramethylpiperidine-1-oxyl (TEMPO, 0.1 mmol/g CNC) and sodium bromide (NaBr, 1 mmol/g CNC) were added under constant stirring. Subsequently, sodium hypochlorite (NaClO, 5 mmol/g CNC) was added dropwise as the oxidizing agent. The pH of the reaction system was

maintained at 10.0 using 0.5 M NaOH, and the reaction proceeded at room temperature for 4 h. To terminate the reaction, ethanol was added, and the resulting mixture was centrifuged at 12,000 rpm for 10 min. The pellet was washed repeatedly with deionized water and then dialyzed against ultrapure water (MWCO 10 kDa) for 48 h to remove residual salts and reagents. The resulting carboxylated cellulose nanocrystal (C-CNC) suspension was collected and stored for subsequent use. To fabricate the hydrogel precursor solution, polyethylene glycol diacrylate (PEGDA, Mn ~700 Da), acrylic acid (AA), and C-CNC suspension were combined in a mass ratio of 4:2:1. PEGDA served as the primary crosslinkable polymer network, AA introduced hydrophilic and reactive carboxylic acid groups, and C-CNCs provided nanoscale rigidity and additional carboxyl functionalities to enhance barrier density. Irgacure 2959 (2 wt%) was added as a photoinitiator, and the mixture was stirred in the dark for 30 min to ensure complete dissolution and homogeneity. The prepolymer solution was gently cast into a pre-cleaned PDMS mold and exposed to UV light (365 nm, 10 mW/cm^2) for 15 min to induce photopolymerization and hydrogel formation. The resulting hydrogel film was demolded at room temperature and rinsed thoroughly with sterile PBS to remove unreacted monomers and residual photoinitiator. The purified hydrogel membrane was collected and designated as PCA.

Characterization of Microneedle Materials and Structures

The compressive strength of PPC and HPF hydrogels was assessed using a universal testing machine (Instron 3343, USA). Disc-shaped samples (6 mm diameter, 6 mm height) were tested at a rate of 1 mm/min at room temperature. The compressive modulus was calculated from the linear region of the stress–strain curve. Electrical conductivity of PPC and HPF was measured using a four-point probe system (Keithley 2400, Lucas Labs S-302-4). Hydrogels were cast into discs (10 mm × 2 mm), equilibrated in PBS, and measured under ambient conditions. Conductivity was calculated based on standard geometric correction factors. PPC, HPF, and PCA hydrogels were lyophilized, fractured, gold-sputtered, and imaged using a field-emission SEM (Hitachi SU8010, 5 kV). Cross-sectional morphology, pore distribution, and network structure were examined. Microneedle arrays were imaged to assess tip geometry, surface integrity, and layer interfaces. Freshly excised rat heart, liver, spleen, lung, and kidney tissues were washed with PBS and placed on moist filter paper. Microneedle patches were gently pressed onto each organ surface for 30 s. The tissue-patch

assemblies were then immersed in PBS for 5 min under mild shaking. Adhesion was evaluated by visual inspection and classified as retained or detached.

Wet Myocardial Adhesion, 180° Peel, and Cyclic Peel Tests

All adhesion and peeling experiments were conducted under a constant temperature of 37 °C with continuous PBS moisturizing. Freshly harvested tissues were transported in pre-cooled PBS (4 °C) and tested within 4 h. Myocardial tissues were trimmed into flat segments (30 × 20 × 2 mm^3 for peel tests; 5 × 5 × 2 mm^3 for probe tack tests) and immobilized on the testing platform. Experimental materials included HPF, PPC, and microneedle (MN) patches. All samples were tested in a blinded, randomized order to ensure experimental consistency and impartiality. Mechanical testing was performed using an Instron 5943 (Instron) equipped with a 50 N load cell at a sampling frequency of 100 Hz. Force–displacement curves were zero-corrected prior to analysis, setting the average force in the final 3–5 mm detachment interval as the baseline. Independent samples (n=7) were used per group, with data expressed as mean ± SD. Group comparisons utilized one-way ANOVA with Tukey's post-hoc correction. Samples were equilibrated in PBS at 37 °C for 10 min pre-test to minimize thermal gradients and dehydration effects on interfacial behavior.

Probe Tack Test: A flat-ended metal probe (5 mm diameter) was compressed against the myocardial surface with a normal stress of 10 kPa for 60 s, then withdrawn vertically at a speed of 100 $\mu m \cdot s^{-1}$ until complete detachment. Pre-defined exclusion criteria included: visible slip with displacement >0.2 mm prior to stable loading; final baseline drift > 0.02 N; or non-interfacial failure peaks caused by tissue tearing or probe contamination. Calculations were performed as follows: $\sigma_{max}(\mathrm{kPa}) = |F_{min}| / A$, $W_{ad}(\mathrm{J \cdot m^{-2}}) = \int |F(\delta)| d\delta / A$. Where A is the contact area (m^2; calculated from the 5 mm probe geometry), F_{min} is the minimum value (negative peak) of the force–displacement curve representing the maximum adhesion force (N), and δ is the probe withdrawal displacement.

180° Peel Test: After applying the sample to the myocardial surface (10 kPa normal stress for 60 s), a 180° peel test was conducted at a constant speed of 100 $\mu m \cdot s^{-1}$ with a peel width b of 5 mm. Analysis excluded the initial 2 mm (establishment phase) and the final 2 mm (failure phase), integrating only the steady-state interval. Exclusion criteria included: myocardial substrate tearing (non-interfacial failure), fixture slippage, undefined effective width due to peel path deviation, or steady-state signal drift > 0.02 N. The interfacial toughness was calculated using the formula:

$G_c(\text{J·m}^{-2}) = W_{peel} / (b·\Delta L)$, where $W_{peel} = \int|F(\delta)|d\delta$. Here, G_c denotes the peel energy per unit area, W_{peel} is the work of peeling in the steady-state region (J), b is the peel width (5 mm), ΔL is the peeling displacement in the steady-state region (m), and $F(\delta)$ is the peel force function.

Cyclic 180° Peel Test: Under bonding conditions identical to the single 180° peel test (10 kPa compression for 60 s; peel arm width 5 mm; 37 °C with continuous PBS moisturizing), samples underwent displacement-controlled reciprocating 180° peeling on the materials tester. The protocol consisted of 8 cycles with a single-cycle stroke of 5 mm and a sampling frequency of 100 Hz. To avoid transient reversal effects, only the middle 1–4 mm of the displacement stroke in each cycle was analyzed as the steady-state interval. The steady-state peel strength was defined as the average peel force within this interval to evaluate cyclic decay and durability. Tests were performed on independent myocardial segments; data interrupted by tissue fatigue fracture, fixture slippage, or peel arm breakage were excluded based on pre-set criteria.

Preparation of Completely Degraded Extracts

To simulate worst-case exposure conditions, sterile microneedle patches were minced and suspended in PBS (pH 7.4) at an extraction ratio of 0.20 g/mL, in strict accordance with ISO 10993-12 standards. To achieve complete depolymerization of the polyester and polysaccharide backbones, the system was supplemented with lipase (*C. rugosa*, 1 mg/mL) and hyaluronidase (1,000 U/mL) and incubated at 37 °C with agitation for 72–120 h until the degradation endpoint (residual dry weight ≤ 5%) was reached. The enzymatic hydrolysates were heat-inactivated (55 °C, 30 min), centrifuged, and clarified via 0.22 μm filtration. The pH of the filtrate was adjusted to 7.2–7.4 using HEPES, and osmolarity was corrected to 310 ± 10 mOsm/kg (hypertonicity caused by neutralization was corrected via dialysis using a 3.5 kDa cutoff to restore isotonicity).

To reconstruct the pathological ionic microenvironment, sterile metal salt stock solutions were added to the extracts under isotonic conditions to achieve final concentrations of 15 μM Cu^{2+} and 15 μM Fe^{2+}. Additionally, 0.1% (w/v) fatty acid-free BSA was added to stabilize the oxidation-prone ferrous ions and buffer ionic activity. Physicochemical properties were verified prior to use; extracts were stored at 4 °C and used within 48 h. For long-term exposure experiments (168 h), the extract was fully replaced every 48 h. A vehicle blank control was prepared using the same enzyme/BSA buffer system, excluding degradation products and exogenous metals.

In Vitro Degradation, Swelling Behavior, and pH Monitoring

To systematically evaluate the responsive degradation and physicochemical stability of the materials within a simulated pathological microenvironment, standardized polymer discs (PPC, HPF, PCA; diameter 8 mm, thickness 2 mm) and complete microneedle arrays (MN) were prepared strictly following the procedures described in the main text. Prior to testing, all samples were annealed and dried in a vacuum oven at 50°C for 48 h until the deviation between two consecutive weighings was less than 0.01 mg; this constant mass was recorded as the initial dry weight (m_0).

The experiment employed an individual container incubation method. Single samples were placed in 50 mL amber glass bottles containing 25 mL of incubation medium (0.9% NaCl supplemented with 1% v/v FBS) sterilized via 0.22 μm filtration, ensuring that the liquid-to-solid ratio strictly met sink conditions (medium/sample mass ratio > 100:1). The bottles were placed in a thermostatic shaker at 37°C with continuous agitation at 60 rpm to simulate body fluid flow and maintain medium homogeneity.

To accurately replicate the temporal ion profile of MI/R, a "cumulative non-replacement" protocol was established in pH 7.40 ± 0.02 equilibrium medium. The baseline group maintained a metal-free environment, while the MI/R simulation group was supplemented with 20 μM $CuCl_2$ at t = 0 h to simulate ischemia, followed by the addition of 100 μM $FeSO_4$ on Day 3 to simulate reperfusion. To preclude oxidative precipitation, all metal stock solutions were freshly prepared using N_2-saturated deoxygenated ultrapure water, stored in an ice bath protected from light, and preheated to 37°C 15 min prior to use.

At preset time points, five independent samples from each group were randomly selected for destructive analysis. Samples were retrieved, and surface free water was removed using quantitative lint-free filter paper (Whatman No. 1) via a standardized blotting procedure (50 g load, 10 s). The wet weight m_{sw} was immediately recorded using a high-precision analytical balance. To accurately distinguish between the swollen gel network and degraded soluble fragments, samples were subsequently transferred to fresh, isothermal blank medium for three vigorous washing steps (60 min each, 100 rpm) to thoroughly remove uncrosslinked or scissioned oligomers and debris. The washed samples were rapidly rinsed with ultrapure water to remove salts and vacuum-dried again

at 50°C to a constant weight m_{dry}. Based on these measurements, the gel fraction and swelling ratio Q_m were calculated using the following formulas:

$$Gel\ fraction(t) = \frac{m_{dry(t)}}{m_0} \times 100\%$$

$$Q_{m(t)} = \frac{m_{sw(t)}}{m_{dry(t)}} \times 100\ \%$$

Curves for residual mass, insoluble gel fraction, and swelling ratio over time were plotted and correlated with EV release percentage $M_0(t)$ on the timeline.

Concurrently, in situ monitoring was performed using a micro pH electrode (Mettler Toledo InLab Micro) to eliminate sampling interference. The electrode underwent three-point calibration daily using standard buffers (pH 4.01/7.00/9.21) at 37°C and was inserted directly into the incubation bottles 2 min before each sampling point to obtain stable readings. To account for non-specific pH fluctuations caused by CO_2 exchange, evaporation, and metal ion oxidation during long-term incubation, the pH of sample-free blank medium was measured synchronously at each time point as a background control. Final reported pH data were blank-corrected to specifically reflect the impact of acidic degradation products on the microenvironment.

Evaluation of Ion-Responsive Stability of PPC and HPF Hydrogels

To verify the responsive disintegration behavior of PPC and HPF hydrogels triggered by specific metal ions within the ischemia/reperfusion microenvironment, exosome-free blank hydrogel samples were prepared. Uncrosslinked PPC or HPF precursor solutions (500 μL) were dispensed into sterile glass vials (10 mL capacity) and incubated statically at 37 °C for 6 h to induce *in situ* gelation via Michael addition.

Subsequently, the gel surfaces were overlaid with 1 mL of PBS (pH 7.4) supplemented with specific metal ions to simulate different pathological conditions: (i) a solution containing only 100 μM $CuCl_2$ to simulate the copper-enriched environment of the ischemic phase; (ii) a mixture of 100 μM $CuCl_2$ and 100 μM $FeSO_4$ to simulate the iron burst environment of the reperfusion phase; or (iii) blank PBS as a negative control.

All systems were incubated statically at 37°C for 2 h, followed immediately by a vial inversion test. Vials were inverted for 10 s, and the macroscopic morphology of the hydrogels was recorded via

high-resolution photography. Any observed deformation, liquefaction, or flow along the vial walls was defined as an indicator of ion-triggered degradation or instability of the hydrogel network.

Ion-Responsive and Stage-Specific Release Characteristics of Hydrogels

To monitor the stage-specific release behavior of PPC and HPF hydrogels under specific metal ion stimulation, a dual-fluorescence labeling strategy was employed to track exosome dynamics. Exosomes derived from $HEY1^{+}$ CMs were incubated with fluorescein isothiocyanate (FITC) or Rhodamine B in the dark. Following the removal of free dye via ultracentrifugation and resuspension in PBS, FITC-labeled exosomes were encapsulated within the PPC precursor solution, while Rhodamine B-labeled exosomes were encapsulated within the HPF precursor solution. The final exosome concentration was adjusted to approximately 50 μg/mL. Subsequently, 100 μL of each cargo-loaded precursor solution was dispensed into 96-well plates and allowed to crosslink to form stable hydrogels. The plates were then incubated in a thermostatic shaker at 37°C with gentle agitation.

Release experiments were conducted in three groups: a non-stimulated control (PBS), a $CuCl_2$ stimulated group, and an $FeSO_4$-stimulated group. At predetermined time points, supernatants were collected, and fluorescence intensity was measured using a multimode microplate reader (Varioskan Flash, Thermo Scientific). The excitation/emission wavelengths were set to 488/520 nm for the FITC channel and 552/580 nm for the Rhodamine B channel. Fluorescence readings were converted into released exosome concentrations based on pre-established standard curves to generate cumulative release profiles. Additionally, fluorescence microscopy was employed for synchronous qualitative observation and imaging analysis of exosome release and diffusion accompanying hydrogel degradation.

Cumulative Release Kinetics of Microneedle Devices in Simulated Pathological Environments

Release kinetics were determined using a CD63 immunocapture–Micro-BCA assay to ensure quantitative consistency with loading efficiency (LE) measurements. First, the baseline for the total releasable amount M_0 for each batch was defined. M_0 was calculated as the theoretical loading amount of a standard 1 cm^2 patch M_{theo} , excluding the blank PCA layer) multiplied by the measured

LE of that batch ($M_0 = M_{theo}$ * LE). This value served as the unified normalization denominator for all subsequent release curves.

An independent destructive sampling design was employed to eliminate volume and concentration biases associated with repeated sampling. At each preset time point (0, 0.25, 0.5, 1, 2, 3, 4, 5, 7, and 10 d), independent microneedle devices were used (n = 5). Individual microneedle patches were placed in PBS buffer (pH 7.4) and incubated at 37°C with constant agitation.

Experimental groups were established to evaluate release behavior under different ionic environments: (i) MN–PBS (passive release control); (ii) MN–Cu^{2+} (20 μM $CuCl_2$ added at t=0); (iii) MN–Fe^{2+} (100 μM $FeSO_4$ added at t=0); and (iv) MN-(Cu^{2+}+Fe^{2+}) simulation group (20 μM $CuCl_2$ added at t=0, followed by 100 μM $FeSO_4$ on Day 3).

At each sampling point, the entire volume of the incubation medium was collected and immediately treated with EDTA (final concentration: 2 mM) at 4°C for a short duration to chelate residual metal ions and eliminate interference with subsequent biochemical assays. Subsequently, the supernatant was incubated with anti-CD63 immunomagnetic beads at 4°C with end-over-end rotation to specifically capture EVs. After thorough washing with buffer to remove soluble contaminant proteins and salts, the bead complexes were subjected to gentle lysis using a lysis buffer containing 0.1% Triton X-100. The lysate was collected, and EV protein concentration was determined using a Micro-BCA kit. Standard curves were prepared using a matrix identical to the samples (containing beads/lysis buffer) to ensure accurate background subtraction. The total amount of EV protein measured at each time point M_t' was converted to a cumulative release percentage using the formula: Cumulative： $Release(\%) = \frac{M_t'}{M_0} \times 100\%$ 。Data are presented as mean ± SD.

Determination of EV Loading and Encapsulation Efficiency

To achieve complete release and precise quantification of EVs embedded within the polymer matrix, an enhanced pretreatment protocol integrating mild detergent lysis with secondary extraction was established. Lyophilized PPC tips, HPF bases, and intact microneedle patches (MN) were precisely weighed W_{total} and subsequently immersed in 1 mL of PBS buffer containing 0.1% Triton X-100. This mild detergent system was designed to disrupt the lipid membrane structure of EVs and weaken

polymer–protein adsorption. Combined with high-intensity ultrasonic homogenization (100 W; 5 s on/5 s off for 5 min), this ensured complete disintegration of the matrix.

The lysate was subjected to high-speed centrifugation (12,000 × *g*, 4°C, 10 min), and the supernatant was collected (Extract A). The resulting pellet was resuspended in 0.5 mL of fresh lysis buffer for a second round of ultrasonic extraction and centrifugation (Extract B). Supernatants from both extractions were combined, and total protein concentration was determined using a Micro-BCA Protein Assay Kit (Beyotime, China). Absorbance was measured at 562 nm using a microplate reader (Varioskan Flash, Thermo Scientific). The total mass of entrapped EVs $m_{entrapped}$ was calculated after subtracting the background interference from the blank microneedle matrix.

To minimize matrix interference on the colorimetric reaction, standard curves were prepared using the supernatant from blank microneedles processed via the same protocol. Blank microneedles treated under identical conditions served as the background subtraction control. The initial feed amount m_{feed} was quantified based on the EV protein mass in the working solution prior to preparation, measured using the same Micro-BCA system. Encapsulation efficiency (EE) and loading efficiency (LE) were calculated using the following formulas:

$$EE(\%) = \frac{m_{entrapped}}{m_{feed}} \times 100\%$$

$$LE(wt\%) = \frac{m_{entrapped}}{W_{total}} \times 100\%$$

Verification of Mitochondrial Exosome Encapsulation and Subtyping via Fluorescence Tracking and Intracellular Immunolabeling

To verify the donor origin and specific mitochondrial subtype encapsulation of EVs, HEY1$^+$ iPSC-CMs overexpressing P5CS or ATP5B were incubated in exosome-depleted medium containing 100 nM MitoTracker Deep Red (Thermo Fisher Scientific) at 37 °C in the dark for 45 min prior to EV collection to pre-label donor mitochondria. After thorough washing with PBS three times to remove free dye, cells were cultured in fresh medium for 48 h. The supernatant was collected, and EV suspensions were prepared via differential ultracentrifugation.

Purified EVs (~20 µg protein) were first incubated with BV421-conjugated anti-CD63 antibody (BD Biosciences) at room temperature in the dark for 30 min to label vesicle membrane structures. Subsequently, samples were fixed with 4% paraformaldehyde (PFA) and permeabilized using a

buffer containing 0.1% saponin. Primary antibodies against P5CS (rabbit polyclonal) and ATP5B (mouse monoclonal) were added and incubated overnight at 4 °C, followed by specific intracellular staining using fluorescent secondary antibodies conjugated to PE and FITC, respectively.

After washing and resuspension in permeabilization buffer, samples were immediately analyzed using a high-sensitivity flow cytometer (Beckman CytoFLEX) for nanoscale particle analysis. The gating strategy first defined the EV population based on CD63-BV421 fluorescence or Side Scatter (SSC) to exclude background noise. Subsequently, within the MitoTracker Deep Red-positive (APC channel) EV subset, the enrichment of specific mitochondrial subtypes was identified based on the two-dimensional scatter distribution of P5CS-PE and ATP5B-FITC fluorescence intensities. To strictly exclude false-positive signals, a 0.1% Triton X-100 detergent lysis group and an isotype IgG control group were processed in parallel under identical labeling and gating conditions.

Analysis of Osmolarity-Dependent EV Uptake Efficiency

To systematically evaluate the regulatory effects of cell membrane tension and the osmotic environment on EV endocytosis efficiency, a gradient culture system spanning from hypotonic to hypertonic conditions was constructed. Basal serum-free medium was precisely titrated with sterile deionized water or D-mannitol to prepare five distinct osmolarity groups (240, 270, 300, 330, and 360 mOsm/kg), with final values validated using a freezing point osmometer (Osmomat 3000, Gonotec). hiPSC-CMs were pre-equilibrated in the aforementioned media for 30 min, followed by the addition of Rhodamine B-labeled EVs (final concentration: 20 μg/mL) and co-incubation at 37°C in the dark for 2 h. Upon completion of incubation, cells were immediately rinsed three times with ice-cold PBS to arrest cellular metabolism and remove non-specifically adsorbed surface particles. Subsequently, cells were fixed with 4% paraformaldehyde (PFA) and counterstained with DAPI. Z-stack imaging was performed using a laser scanning confocal microscope (LSM 880, Zeiss). The corrected total cell fluorescence (CTCF) was calculated using ImageJ software to quantify the total EV uptake per single cell after background noise subtraction.

Biocompatibility Evaluation

IPSC-CMs were maintained under standard culture conditions (37 °C, 5% CO_2) and seeded into 24-well plates at a density of 2×10^5 cells per well. Sterile hydrogel discs (8 mm in diameter, 1 mm in

thickness), pre-equilibrated in culture medium overnight, were placed into each well. The hydrogels evaluated included PPC, HPF, and PCA. Wells without hydrogels served as the blank control. Cells were co-cultured with the hydrogels for a total of 168 h. At predetermined time points (0, 24, 72, 120, and 168 h), Cell Counting Kit-8 (CCK-8) reagent was added to each well at 10% of the culture medium volume. After incubation for 2 h, cell viability was assessed by measuring absorbance at 450 nm.

In Vivo Fluorescence Imaging of Microneedle Behavior

Following the establishment of a rat myocardial infarction (MI) model via left anterior descending (LAD) artery ligation, Rhodamine B-loaded PPC/HPF bilayer microneedle patches (excluding the PCA layer) were applied to the infarcted region. Rats were sacrificed at predetermined time points (days 0, 1, 3, 5, 7, and 15), and major organs (heart, liver, spleen, lung, and kidney）were harvested. *Ex vivo* fluorescence imaging was performed using an IVIS imaging system (excitation/emission wavelengths: 540/580 nm) to visualize the biodistribution of Rhodamine. To evaluate the advantages of microneedle-mediated tissue retention compared to conventional administration, two groups were established: a microneedle delivery group (MN) and an epicardial injection group (EI). In the MN group, microneedle patches loaded with Rhodamine B-labeled exosomes were implanted. Conversely, in the EI group, an equivalent amount of Rhodamine B-labeled exosome suspension was administered via multi-point injection into the infarcted region using a microsyringe. Excised hearts were obtained at predetermined time points (days 0, 1, 3, 5, 7, 11, and 15). The residual fluorescence radiant efficiency within the myocardial tissue was quantified using the IVIS system to compare the local retention duration and spatial distribution of exosomes between the two delivery methods. In a separate experiment, microneedle patches comprising a FITC-labeled PPC layer and a Rhodamine-labeled HPF layer, coated with a PCA barrier layer, were utilized. Following application of the patch to the rat MI model, organs (heart, liver, spleen, lung, and kidney) were harvested on days 0, 1, 3, 5, 7, 9, and 15 for IVIS imaging (FITC: 488/520 nm; Rhodamine: 540/580 nm).

Establishment of Porcine Myocardial Ischemia-Reperfusion Model and Therapeutic Interventions

All animal experiments were approved by the Animal Ethics Committee of Sichuan Provincial People's Hospital. Bama minipigs (15–20 kg) were obtained from Chengdu Dosssy Experimental Animals Co., Ltd. (Chengdu, China). and fasted for 12 h prior to surgery. Anesthesia was induced via intramuscular injection of ketamine (10 mg/kg) and xylazine (2 mg/kg), followed by tracheal intubation and mechanical ventilation. General anesthesia was maintained intraoperatively by inhalation of 1.5–2% isoflurane. A left thoracotomy was performed via the fourth intercostal space to expose the heart. Upon opening the pericardium, the LAD was identified 1–1.5 cm below the left atrial appendage. An MI/R model was established by ligating the LAD using 4-0 silk sutures. Local myocardial cyanosis and ST-segment elevation on the electrocardiogram (ECG) served as indicators of successful ischemia. The ligature was released after 60 min of ischemia to initiate reperfusion. The animals were randomized into five groups: the Sham group underwent threading without ligation to simulate surgical stress; the Saline group received 200 μL of physiological saline applied dropwise to the infarcted zone post-MI/R; the MN group was implanted with blank microneedle patches; the MixEvs group received a direct dropwise application of the exosome mixture (Ev(MT^{P5CS}):Ev(MT^{ATP5B}) = 1:2); and the MN(MixEvs) group was implanted with microneedle patches loaded with the mixed exosomes. 24h-post-treatment, cardiac function was assessed via ECG, and peripheral blood was collected to measure cTnI and CK-MB levels to quantify the extent of myocardial injury.

Echocardiographic Functional Assessment

Four weeks post-treatment, transthoracic echocardiography was performed on minipigs using a veterinary ultrasound imaging system. Animals were placed in the right lateral decubitus position under light anesthesia maintained with 1–1.5% isoflurane inhalation. Standard parasternal long-axis and short-axis views were obtained. M-mode echocardiography was utilized to measure left ventricular end-diastolic diameter (LVEDD) and end-systolic diameter (LVESD), from which left ventricular ejection fraction (LVEF) and fractional shortening (LVFS) were calculated to assess systolic function.Additionally, Pulsed-Wave Doppler was employed to record mitral inflow patterns. Peak early diastolic filling velocity (E wave) and late diastolic atrial contraction velocity (A wave) were measured, and the E/A ratio was calculated to evaluate diastolic function. Heart rate (HR) was

derived synchronously from M-mode tracings. All echocardiographic images were digitally stored and analyzed offline using professional workstation software in a blinded manner.

Quantification of Myocardial Infarct Size and Fibrosis

Following functional assessment, animals were euthanized under deep anesthesia. The hearts were rapidly excised and rinsed with ice-cold saline to remove residual blood. Subsequently, the hearts were transversely sectioned from the apex to the base into five serial slices, each approximately 5 mm thick. The slices were incubated in a 1% 2,3,5-triphenyltetrazolium chloride (TTC) solution at 37°C in the dark for 20 min, followed by fixation in 4% paraformaldehyde to enhance contrast.TTC staining differentiates tissue viability based on dehydrogenase activity: viable myocardium stains brick red, whereas infarcted or fibrotic regions appear pale (unstained). Digital photographs of both sides of each slice were acquired. The infarct area and total left ventricular (LV) cross-sectional area were digitally measured using ImageJ software. The extent of myocardial fibrosis was defined as the percentage of the infarct area relative to the total LV area $Fibrosis(\%) = \frac{\text{Infarct Area}}{\text{Total LV Area}} \times 100\%$. Final results were expressed as the mean value of all slices for each heart.

Detection of cTnI and CK-MB

Serum cTnI levels were determined using a specific enzyme-linked immunosorbent assay (ELISA) kit (TNNI3 ELISA kit; ELK Biotechnology). All detection procedures were performed strictly in accordance with the manufacturer's instructions. Concurrently, CK-MB levels were assessed using a species-specific kit provided by Coibo. Following the successful establishment of the model in each experimental group, 0.2 mL of venous blood was collected for the quantification of these markers.

Ex vivo Optical Mapping and Electrophysiological Assessment

To facilitate high-spatiotemporal-resolution assessment of cardiac electrophysiology, Bama minipigs received systemic anticoagulation via intraperitoneal injection of heparin (3000 U/kg) 15 min prior to the induction of anesthesia. Subsequently, hearts were rapidly excised via thoracotomy

under deep isoflurane anesthesia and immediately mounted onto a Langendorff perfusion system. Retrograde perfusion was initiated using oxygenated, ice-cold (4°C) Ca^{2+}-free Krebs buffer. After a 10-min stabilization period, the electromechanical uncoupler Blebbistatin was added to the perfusate to inhibit motion artifacts caused by mechanical contraction. Subsequently, the co-solvent Pluronic F127, the Ca^{2+} indicator Rhod-2 AM, and the voltage-sensitive dye RH237 were loaded sequentially.

Following staining, the heart was placed in an optical imaging chamber equipped with a multi-channel recording system. Recording electrodes were positioned as follows: the anode at the left ventricular apex, the cathode at the right atrium, and the reference ground electrode at the bottom of the perfusion bath.

Optical and electrophysiological signals were acquired during sinus rhythm, fixed-rate pacing (6 Hz), and Ca^{2+} transient dynamics. The ventricular effective refractory period (VERP) was determined via a standard S1-S2 programmed stimulation protocol, consisting of a fixed S1 drive train followed by S2 extrastimuli with progressively shortened coupling intervals until loss of capture. Additionally, a 50 Hz burst pacing protocol with incremental current intensities (5–20 mA) was employed to induce and assess susceptibility to arrhythmias. Finally, action potential duration (APD), depolarization/repolarization kinetics, and intracellular Ca^{2+} cycling characteristics were comprehensively analyzed using synchronously recorded ECG and optical fluorescence signals.

In situ TUNEL Apoptosis Detection and Stratified Quantitative Evaluation

To assess cardiomyocyte viability in the infarct border zone and the transmural drug delivery efficacy post-treatment, experimental animals were euthanized under deep anesthesia 24 h after treatment, and hearts were rapidly harvested. For the mouse model, tissues from the infarct border zone were routinely collected. Conversely, for the Bama minipig model, to precisely evaluate the vertical penetration depth of microneedles, tissues from the infarct border zone were sampled via a refined stratification method based on the vertical distance from the epicardial surface: superficial (0–1 mm), middle (1–3 mm), and deep layers (3–8 mm). All tissue samples were embedded in OCT compound, snap-frozen in liquid nitrogen, and sectioned into 5 μm thick slices using a cryostat.

Apoptosis was detected using a commercial Terminal deoxynucleotidyl transferase dUTP Nick-End Labeling (TUNEL) kit (Roche), strictly following the manufacturer's instructions. After rewarming

to room temperature, sections were fixed with freshly prepared 4% paraformaldehyde (PFA) for 15 min. Following a PBS wash, samples were permeabilized by immersion in sodium citrate buffer containing 0.1% Triton X-100 on ice to enhance membrane permeability.

Subsequently, the TUNEL reaction mixture containing TdT enzyme and fluorescently labeled dUTP was added dropwise. Slides were incubated in a humidified chamber at 37°C in the dark for 60 min. After termination of the reaction, nuclei were counterstained using a mounting medium containing DAPI.

Multi-channel imaging was performed using a fluorescence microscope to identify green-fluorescent TUNEL-positive cells and blue-fluorescent nuclei. Quantitative analysis employed a stratified strategy. For mouse samples, five non-overlapping fields were randomly selected from the border zone of each section for counting. For Bama minipig samples, the superficial, middle, and deep layers were imaged and quantified independently to elucidate the spatial distribution of therapeutic efficacy. The apoptosis index was defined as the percentage of TUNEL-positive nuclei relative to the total number of DAPI-labeled nuclei.

In Vivo Biosafety and Systemic Toxicity Assessment

To comprehensively evaluate the biocompatibility of the microneedle therapy system, acute and long-term toxicological assessments were conducted in a Bama minipig model. 24h- post-treatment, peripheral blood was collected via the ear marginal vein. Acute liver and kidney function indicators (UA, Urea, γ-GT, and AST) were analyzed using an automatic biochemical analyzer. Additionally, longitudinal safety monitoring was performed over a 28-day period. Serial blood samples were obtained at predetermined time points to map the dynamic profiles of ALT, TBIL, BUN, and CREA. At the experimental endpoint, major organs—including the heart, liver, spleen, lung, and kidney—were harvested, fixed in 10% neutral buffered formalin, embedded in paraffin, and sectioned at a thickness of 5 μm. Sections were stained with hematoxylin and eosin (H&E) and examined under an optical microscope to evaluate tissue morphology and inflammatory infiltration, thereby determining potential systemic toxicity.

Transmission Electron Microscopy Observation of Cardiac Mitochondria

On day 3 post-treatment, minipigs were euthanized under deep anesthesia, and myocardial tissue samples from the infarct border zone were harvested. Tissue blocks (approximately 1 mm^3) were immediately excised and fixed in 2.5% glutaraldehyde at 4 °C overnight.Subsequently, the samples were post-fixed with 1% osmium tetroxide for 1 h, followed by graded ethanol dehydration, embedding in epoxy resin, ultrathin sectioning (thickness ~70 nm), and double staining with uranyl acetate and lead citrate.Finally, the samples were examined using a TEM. At least five fields were randomly selected from each sample to evaluate mitochondrial morphology and structure.

Western Blot Analysis

In accordance with the experimental design, myocardial tissues were harvested from the infarct border zone at predetermined time points post-treatment (days 1, 3, and 28), while cell pellets were collected for in vitro assays following their respective treatments. Total protein was extracted by homogenizing tissues or lysing cells in RIPA lysis buffer supplemented with protease and phosphatase inhibitors. Protein concentrations were determined using a BCA assay. Subsequently, protein samples were separated via SDS-PAGE and transferred onto PVDF membranes.Membranes were incubated with specific primary antibodies tailored to the experimental objectives. For the in vitro screening and characterization of the mitochondrial metabolic network, antibodies against P5CS, ATP5B, ALDH1L2, SLC25A39, NADK2, TXNIP, GCN5L1, CKMT2, LIPT1, CLUH, MUL1, BCKDK, CD9, VDAC1, and TOMM20 were utilized. To assess acute metabolic stress and apoptosis on day 1 in vivo, markers including HK2, PDK1, LDHA, Cleaved Caspase-3, BAX, BCL-2, and Cytochrome c were probed. For the evaluation of metabolic remodeling, calcium homeostasis, and autophagic flux on day 3, antibodies against PPARα, CPT1B, SERCA2a, NCX1, CASQ2, LC3, p62, Parkin, and OPTN were employed. Long-term mitochondrial biogenesis and homeostasis on day 28 were assessed using PGC-1α, COX IV, and PINK1. GAPDH and β-actin served as internal loading controls. Following primary antibody incubation, membranes were incubated with HRP-conjugated secondary antibodies, and protein bands were visualized using an enhanced chemiluminescence (ECL) substrate. All antibodies were purchased from Proteintech.

Differential Gene Expression Profiling and Bioinformatics Enrichment Analysis

To elucidate the underlying in vivo therapeutic mechanisms, total RNA was isolated from the infarct

border zone of Bama minipigs on day 3 post-treatment using TRIzol Reagent. Following quality control verification of RNA integrity numbers (RIN > 7.0), samples were submitted to Tsingke Biological Technology Co., Ltd. for library construction and high-throughput sequencing.

The resulting sequencing data were subjected to differential expression analysis. Volcano plots were generated to visualize the global shift in gene expression profiles between the MN(MixEvs) treatment group and the control group, aiming to evaluate the phenotypic transition from pathological glycolysis to physiological oxidative metabolism. Subsequently, KEGG pathway analysis and GSEA were jointly employed to systematically elucidate the regulatory effects of microneedle therapy on critical signaling pathways, including oxidative phosphorylation, fatty acid metabolism, HIF-1 signaling, and apoptosis.

Synthesis of Polydopamine Nanoparticles and Myocardial Ischemia/Reperfusion with In Situ Injection Model

Polydopamine nanoparticles (PDA NPs) were synthesized via oxidative self-polymerization. Briefly, dopamine hydrochloride (Sigma-Aldrich, H8502) was dissolved in 10 mM Tris-HCl buffer (pH 8.5) to a final concentration of 2 mg/mL and subjected to vigorous stirring at room temperature in the dark for 24 h. The resulting product was purified and washed via centrifugation at 15,000 rpm for 20 min, resuspended in sterile PBS, and physicochemically characterized using TEM (JEOL, JEM-2100F) and dynamic light scattering (DLS; Malvern Zetasizer Nano ZS).

For in vivo experiments, male C57BL/6 mice (8–10 weeks old, 22–25 g) were anesthetized with 2% isoflurane and intubated. Myocardial ischemia was induced by LAD coronary artery for 45 min, after which the ligature was released to initiate reperfusion, thereby establishing the MI/R model. Immediately upon reperfusion, a total volume of 15 μL of saline or PDA suspension was administered via in situ injection at three distinct points within the infarct border zone using a microsyringe equipped with a 30G needle. This procedure ensured uniform delivery of the therapeutic agent to the damaged region. Subsequently, the chest cavity was sutured in layers, and the animals were monitored until full recovery.

Evaluation of Differential Uptake of Exosome Cargo by Distinct Cardiac Cell Types

To investigate the cellular affinity profile of engineered exosomes within the cardiac

microenvironment, human iPSC-CMs, human cardiac fibroblasts (CFs), human umbilical vein endothelial cells (ECs), and human coronary artery smooth muscle cells (SMCs) were seeded into 12-well plates at a density of 1×10^5 cells/well.Upon complete cell attachment, Rhodamine B-labeled mixed exosomes (MixEVs) were added to each well at a standardized final concentration of 20 μg/mL and co-incubated at 37°C in the dark for 2 h. Following incubation, cells were rinsed three times with ice-cold PBS to remove non-specifically adsorbed surface particles, and single-cell suspensions were prepared via digestion with 0.25% trypsin.Samples were acquired using a flow cytometer (CytoFLEX, Beckman Coulter), with the acquisition strategy set to record all available events per sample for analysis within the single-cell gate. Data analysis was performed using FlowJo software. Exosome uptake levels were quantified via geometric mean fluorescence intensity (geoMFI). The threshold for defining the positive cell population was established based on untreated negative control samples (setting the false positive rate at < 1%) to calculate the percentage of exosome-positive cells.

Supplementary data

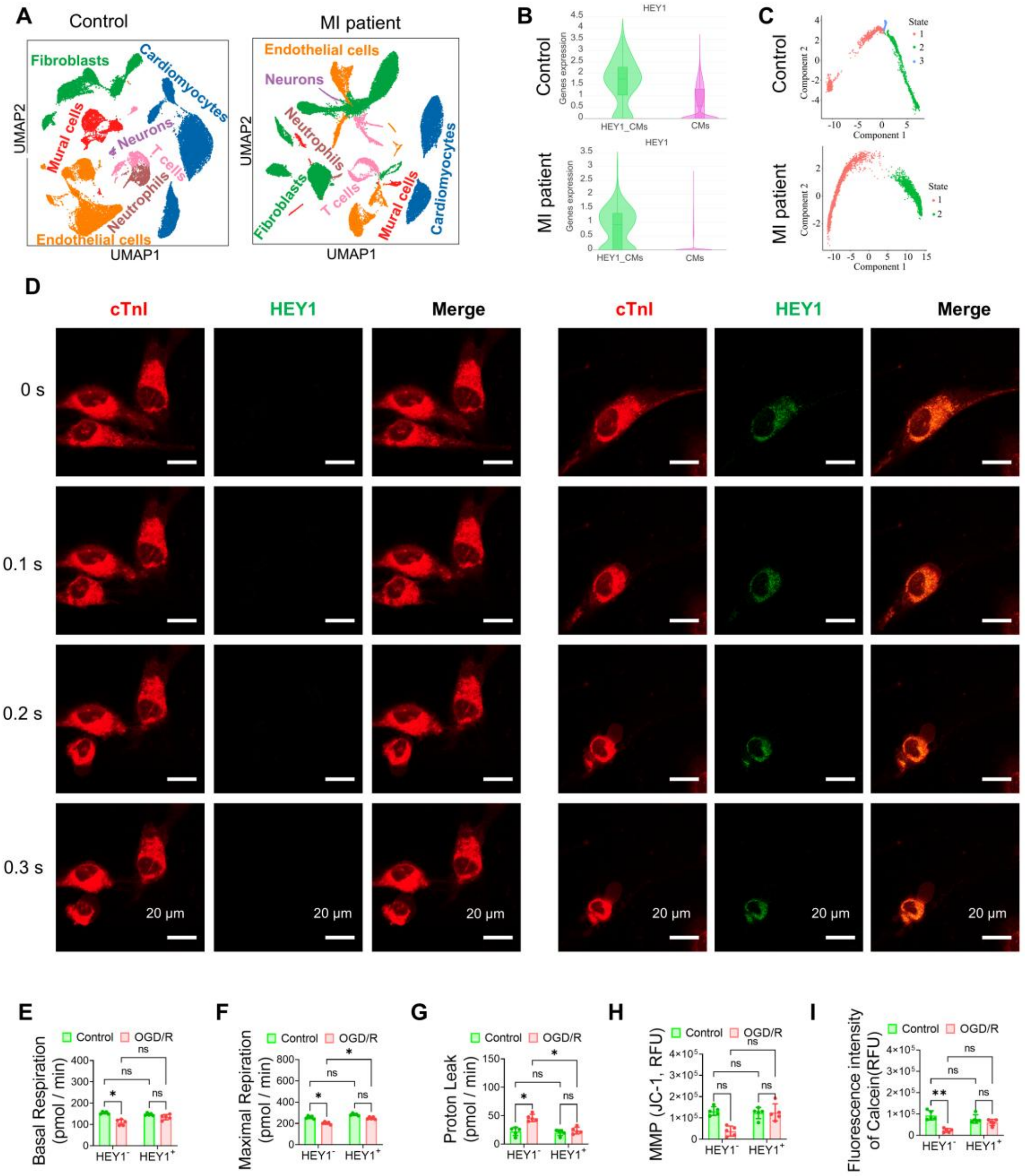

Figure S1: **(A)** UMAP plot of single-cell RNA sequencing (scRNA-seq) data illustrating annotated cell types in myocardial tissues from healthy donors and patients with MI. **(B)** Violin plots comparing HEY1 expression levels between HEY1$^+$ CMs and conventional cardiomyocytes (CMs) across both control and MI groups. **(C)** Pseudotime trajectory analysis depicting the developmental and differentiation dynamics of cardiomyocytes under physiological and pathological conditions. **(D)** Confocal microscopy images of iPSC-derived HEY1$^+$ CMs and HEY1$^-$CMs capturing synchronous beating behaviors at distinct differentiation time points. **(E–I)** Supplementary assessment of mitochondrial respiratory function and homeostasis in HEY1$^+$ CMs versus HEY1$^-$CMs under OGD/R conditions, including: **(E)** basal

respiration; **(F)** maximal respiration capacity; **(G)** proton leak; **(H)** mitochondrial membrane potential (JC-1); and **(I)** the extent of mitochondrial permeability transition pore (mPTP) opening. **Statistics**: Data are presented as mean ± SD; the independent sample size (*n*) is indicated by data points/labels in the figures. One-way ANOVA with Tukey's multiple-comparison correction was used for single-factor multi-group comparisons. Two-way ANOVA with Sidak's or Tukey's multiple-comparison correction was used for two-factor designs. **P* < 0.05, ***P* < 0.01, ****P* < 0.005, *****P* < 0.001; *ns*, not significant.

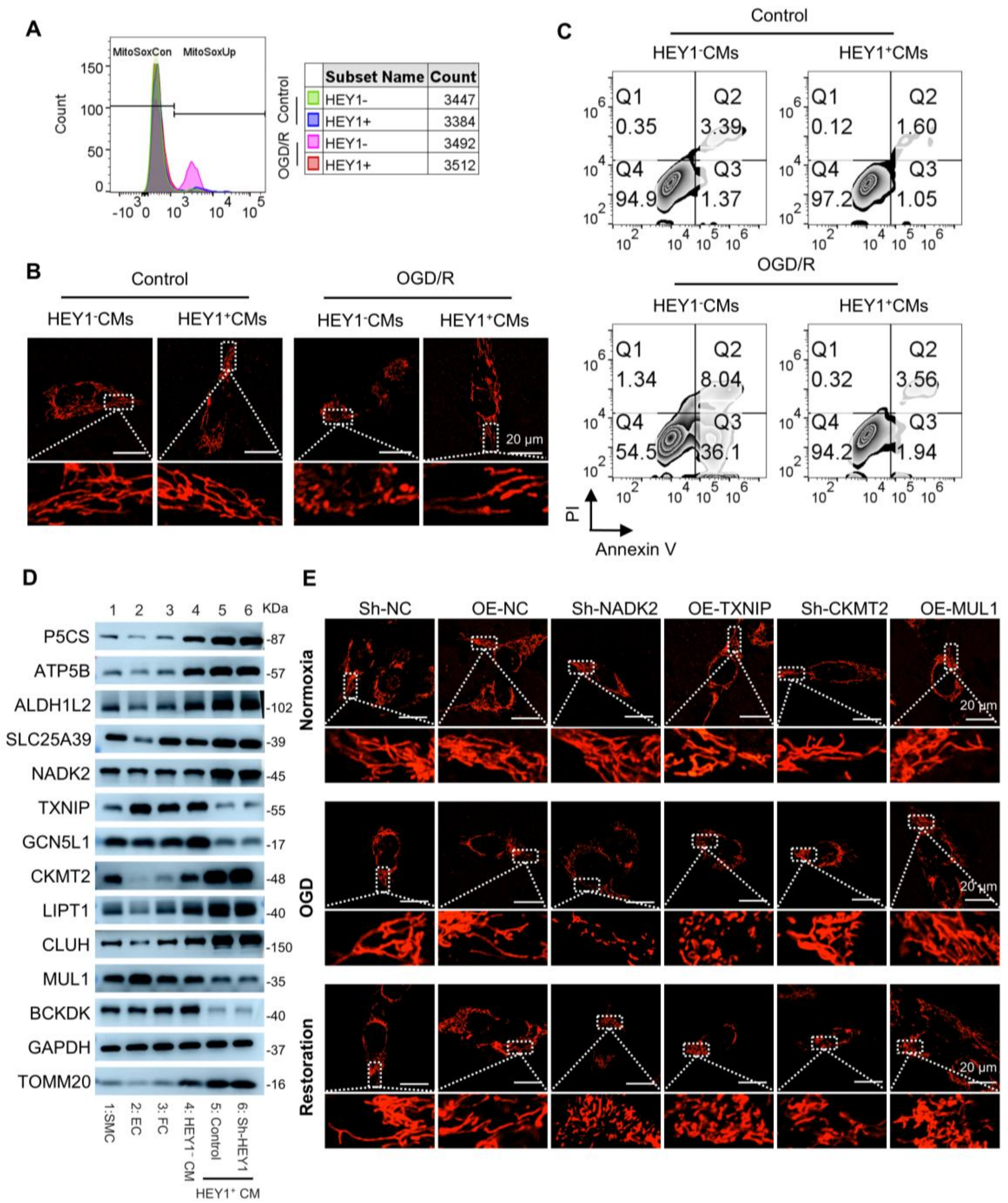

Figure S2: **(A)** Flow cytometric assessment of mitochondrial superoxide levels using MitoSOX

fluorescence to compare oxidative stress between HEY1$^+$ and HEY1$^-$ cardiomyocytes under control and OGD/R conditions. **(B)** Confocal microscopy images illustrating differences in mitochondrial morphology between HEY1$^+$ and HEY1$^-$ cardiomyocytes across different conditions. HEY1$^+$ cells exhibit a more intact mitochondrial network and elongated mitochondrial morphology. **(C)** Analysis of cell death via flow cytometry using Annexin V/PI double staining to quantify apoptotic rates in HEY1$^+$ versus HEY1$^-$ cardiomyocytes under control and OGD/R conditions. **(D)** Western blot analysis comparing the basal expression profiles of candidate proteins across different cell types and HEY1 contexts. Probed proteins include mitochondrial subtype markers (P5CS, ATP5B), the mitochondrial mass marker TOMM20, and candidate regulatory nodes (ALDH1L2, SLC25A39, NADK2, TXNIP, GCN5L1, CKMT2, LIPT1, CLUH, MUL1, and BCKDK). **(E)** Confocal microscopy images of HEY1$^+$ iPSC-CMs following knockdown or overexpression of candidate factors (Groups: sh-NC, OE-NC, sh-NADK2, OE-TXNIP, sh-CKMT2, and OE-MUL1). Images were acquired during normoxia, OGD, and reperfusion phases to demonstrate changes in mitochondrial morphology under different treatment conditions.

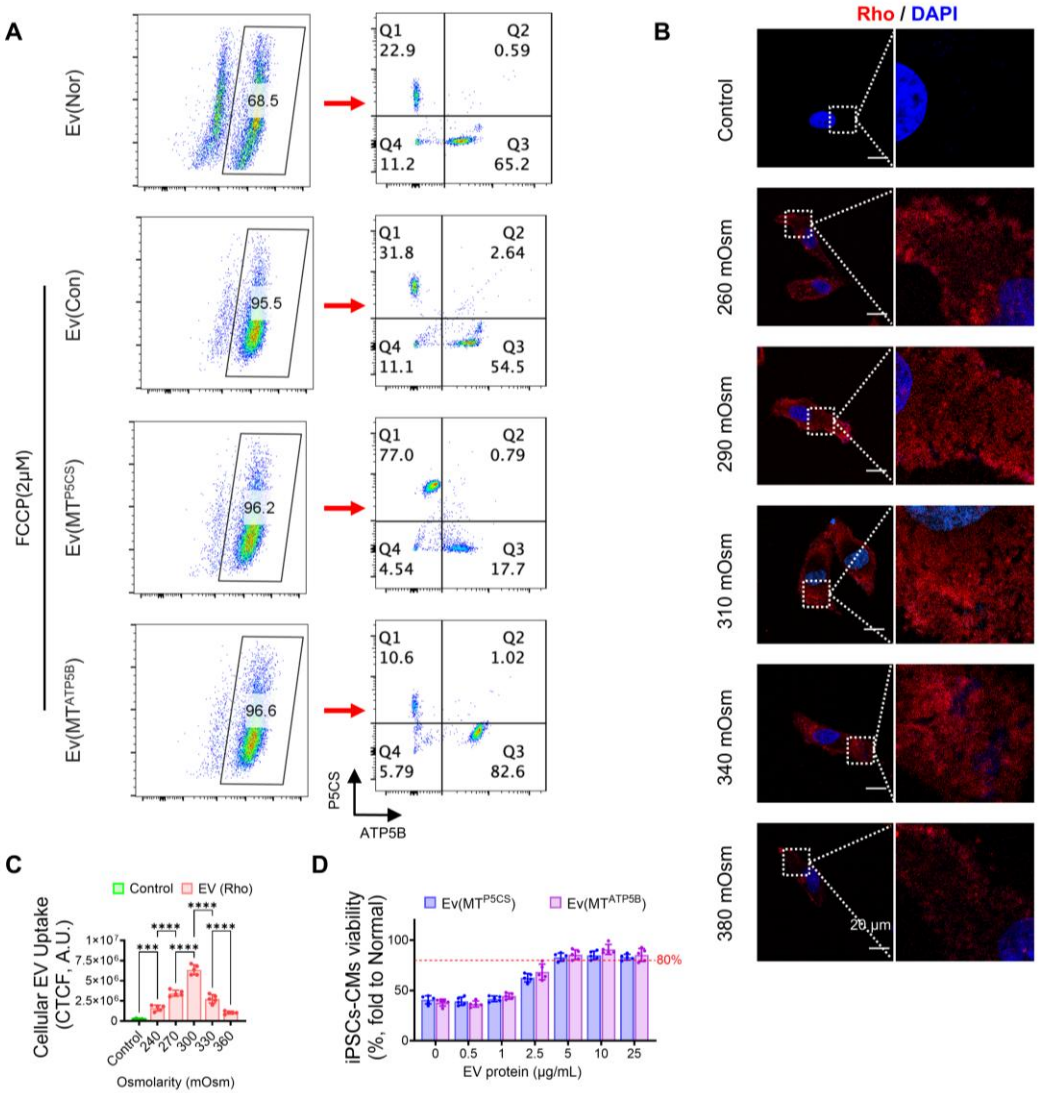

Figure S3: **(A)** Single-particle analysis and subtype validation of engineered EVs using high-sensitivity nano-flow cytometry. **(B)** Representative confocal microscopy images of iPSC-CMs co-incubated with Rhodamine-labeled EVs (red) under varying osmotic conditions. **(C)** Quantification of EV uptake. Uptake exhibited a bell-shaped distribution in response to osmolarity changes, peaking at 300 mOsm and declining under both hypotonic and hypertonic conditions. **(D)** Cell viability of iPSC-CMs assessed via CCK-8 assay following OGD/R treatment and administration of varying EV protein concentrations. Viability increased in a dose-dependent manner, reaching a plateau of ≥80% within the 5–25 μg/mL range. **Statistics**: Data are presented as mean ± SD; the independent sample size (*n*) is indicated by data points/labels in the figures. One-way ANOVA with Tukey's multiple-comparison correction was used for single-factor multi-group comparisons. Two-way ANOVA with Sidak's or Tukey's multiple-comparison correction was used for two-factor designs. $^{*}P < 0.05$, $^{**}P < 0.01$, $^{***}P < 0.005$, $^{****}P < 0.001$; *ns*, not significant.

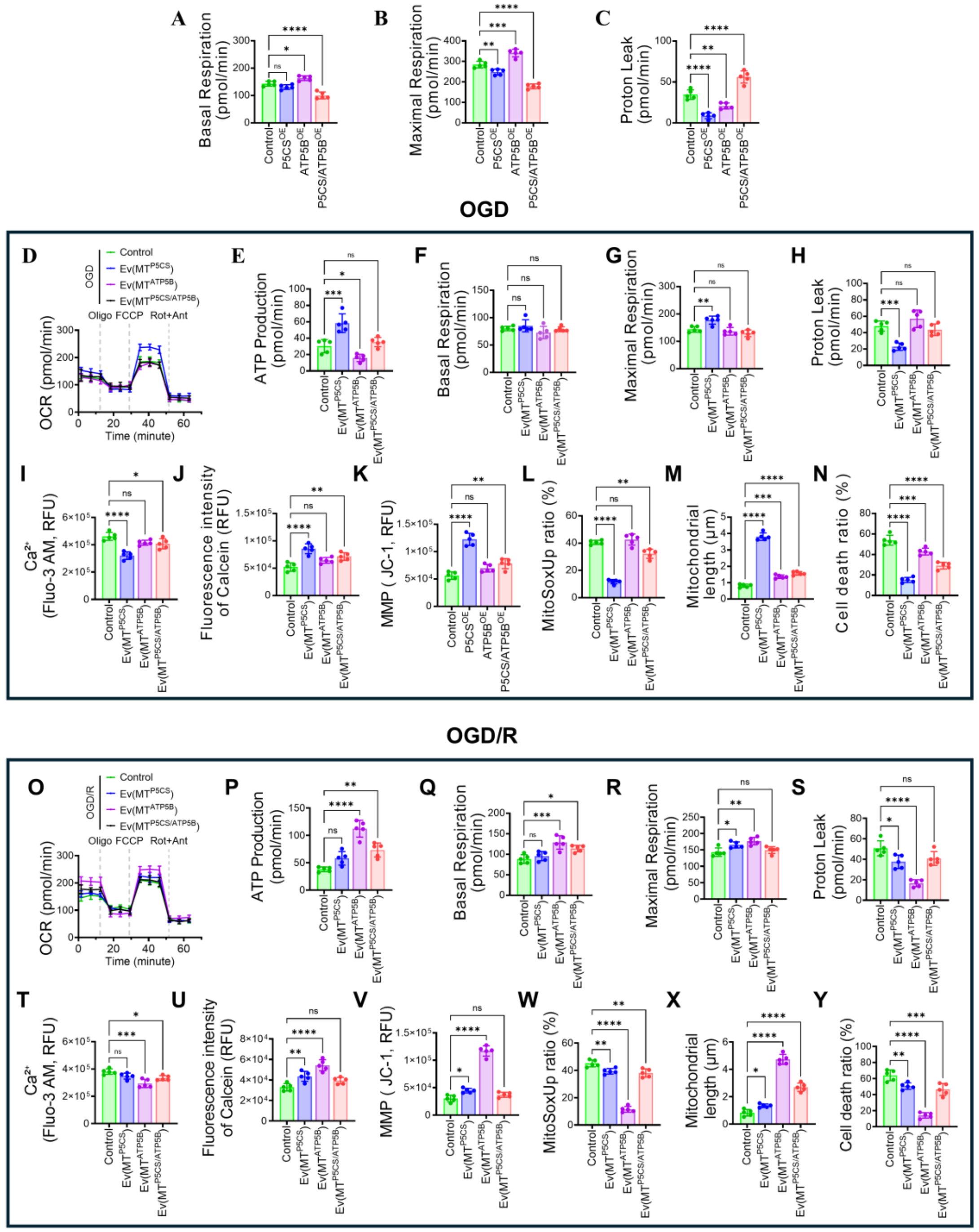

Figure S4: (A–C) Evaluation of mitochondrial respiratory parameters in HEY1^{+} cardiomyocytes under single-gene overexpression (P5CS^{OE} or ATP5B^{OE}) versus simultaneous dual overexpression (P5CS/ATP5B^{OE}) conditions using a Seahorse XF analyzer. Quantitative analyses include **(A)** basal respiration rate, **(B)** maximal respiration capacity, and **(C)** proton leak. **(D–Y)** Comparative therapeutic efficacy of conventional EVs [Ev(Nor)], single-subtype EVs [Ev(MT^{P5CS}) or Ev(MT^{ATP5B})], and dual-cargo EVs [Ev(MT$^{P5CS/ATP5B}$)] in an iPSC-CM model. **(D–N)** Detailed functional assessment under OGD conditions: **(D)** Real-time traces of the mitochondrial stress test (OCR); **(E)** ATP production rate; **(F)**

basal respiration; **(G)** maximal respiration capacity; **(H)** proton leak; **(I)** intracellular Ca^{2+} concentration (Fluo-3 AM); **(J)** extent of mPTP opening (Calcein fluorescence); **(K)** mitochondrial membrane potential (JC-1); **(L)** mitochondrial ROS levels (MitoSOX); **(M)** quantification of mitochondrial length; and **(N)** cell death rate evaluated via Annexin V/PI double staining. **(O–Y)** Detailed functional assessment under OGD/R conditions: **(O)** Real-time traces of the mitochondrial stress test (OCR); **(P)** ATP production rate; **(Q)** basal respiration; **(R)** maximal respiration capacity; **(S)** proton leak; **(T)** intracellular Ca^{2+} concentration; **(U)** extent of mPTP opening; **(V)** mitochondrial membrane potential; **(W)** mitochondrial ROS levels; **(X)** quantification of mitochondrial length; and **(Y)** cell death rate evaluated via Annexin V/PI double staining. **Statistics**: Data are presented as mean ± SD; the independent sample size (*n*) is indicated by data points/labels in the figures. One-way ANOVA with Tukey's multiple-comparison correction was used for single-factor multi-group comparisons. Two-way ANOVA with Sidak's or Tukey's multiple-comparison correction was used for two-factor designs. $^{*}P < 0.05$, $^{**}P < 0.01$, $^{***}P < 0.005$, $^{****}P < 0.001$; *ns*, not significant.

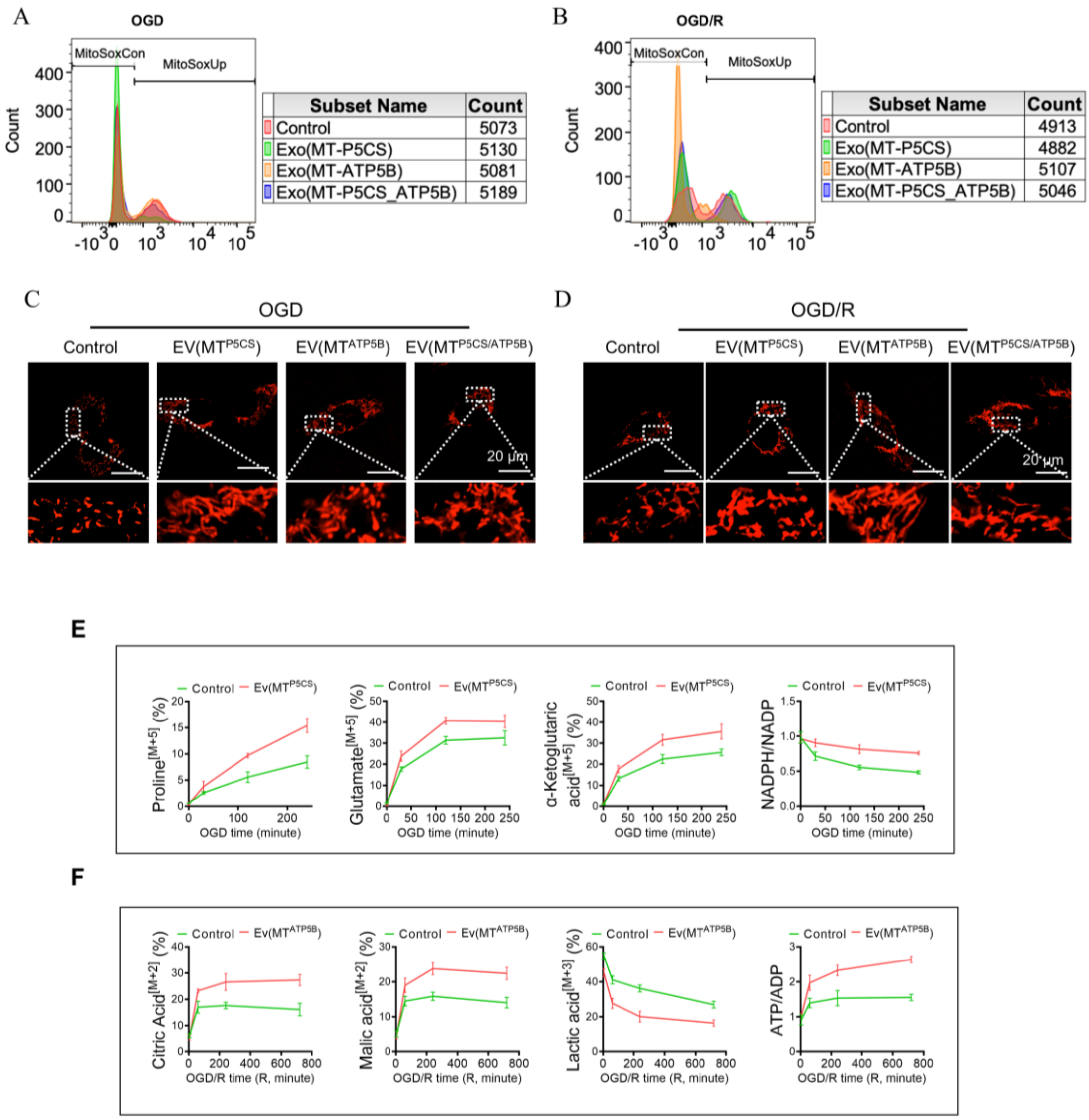

Figure S5: (A–B) Flow cytometric analysis of mitochondrial superoxide levels using MitoSOX staining. The oxidative stress status of iPSC-CMs was assessed under **(A)** oxygen-glucose deprivation (OGD) and **(B)** OGD/reperfusion (OGD/R) conditions following treatment with single-cargo [Ev(MT^{P5CS}) or Ev(MT^{ATP5B})] or dual-cargo [Ev(MT$^{P5CS/ATP5B}$)] EVs. The table to the right of the histograms presents cell count statistics for each subgroup. **(C–D)** Quantitative analysis of mitochondrial morphology. Data represent the mean mitochondrial length (µm) for each treatment group under **(C)** OGD and **(D)** OGD/R conditions, comparing the structural preservation capability of the dual-cargo strategy against the optimal single-cargo strategy. **(E–F)** Phasic metabolic remodeling of P5CS and ATP5B revealed by ^13C isotope tracing. **(E)** During the ischemic phase, ^13C_5-glutamine tracing demonstrated that the Ev(MT^{P5CS}) group significantly increased glutamine flux (Glutamate [M+5], α-Ketoglutarate [M+5], Proline [M+5]) and elevated the NADPH/NADP$^+$ ratio, suggesting that P5CS facilitates adaptation to ischemia by enhancing

the cellular reductive state. **(F)** During the reperfusion phase, ^13C$_6$-glucose tracing showed that the Ev(MT^{ATP5B}) group enhanced carbon flux into the tricarboxylic acid (TCA) cycle (Citrate [M+2], Malate [M+2]), reduced lactate [M+3] production, and increased the ATP/ADP ratio, indicating that ATP5B promotes oxidative phosphorylation and energy recovery. **Statistics**: Data are presented as mean ± SD; the independent sample size (*n*) is indicated by data points/labels in the figures. One-way ANOVA with Tukey's multiple-comparison correction was used for single-factor multi-group comparisons. Two-way ANOVA with Sidak's or Tukey's multiple-comparison correction was used for two-factor designs. $*P < 0.05$, $**P < 0.01$, $***P < 0.005$, $****P < 0.001$; *ns*, not significant.

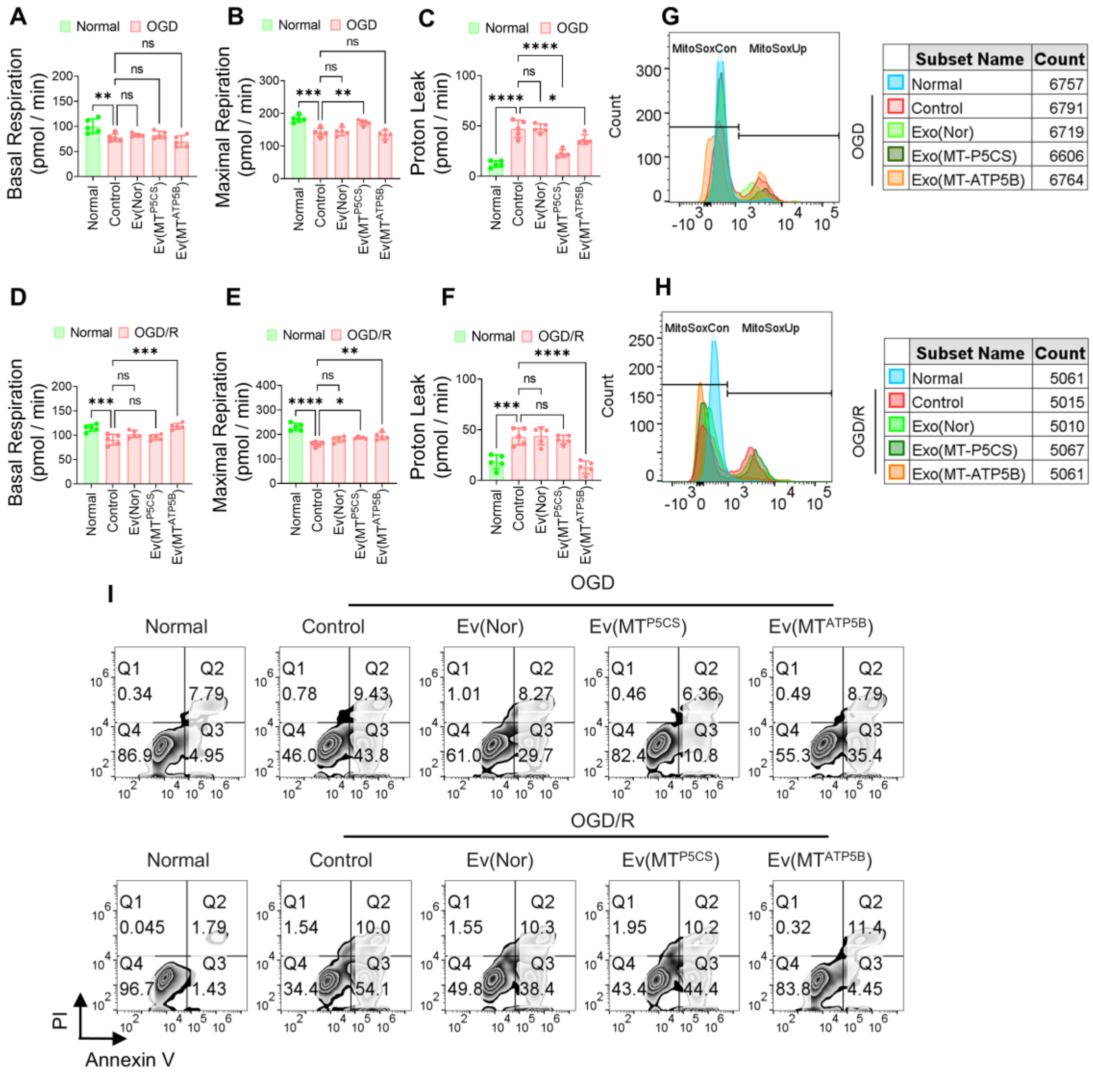

Figure S6: iPSC-CMs were subjected to OGD or OGD/R stress and treated with different exosome formulations (Ev(Nor), Ev(MT^{P5CS}), or Ev(MT^{ATP5B})). Mitochondrial function was evaluated via Oxygen Consumption Rate (OCR) analysis. **(A–C)** Quantification of OCR parameters under OGD conditions:

(A) basal respiration; **(B)** maximal respiration capacity; and **(C)** proton leak. **(D–F)** Quantification of OCR parameters under OGD/R conditions: **(D)** basal respiration; **(E)** maximal respiration capacity; and **(F)** proton leak. **(G–H)** Analysis of mitochondrial ROS levels using MitoSOX staining coupled with flow cytometry. Cell count statistics for each group are presented in the table to the right. **(I)** Assessment of cell death via Annexin V/PI double staining following the respective treatments under OGD and OGD/R stress conditions. **Statistics**: Data are presented as mean ± SD; the independent sample size (*n*) is indicated by data points/labels in the figures. One-way ANOVA with Tukey's multiple-comparison correction was used for single-factor multi-group comparisons. Two-way ANOVA with Sidak's or Tukey's multiple-comparison correction was used for two-factor designs. *$P < 0.05$, **$P < 0.01$, ***$P < 0.005$, ****$P < 0.001$; *ns*, not significant.

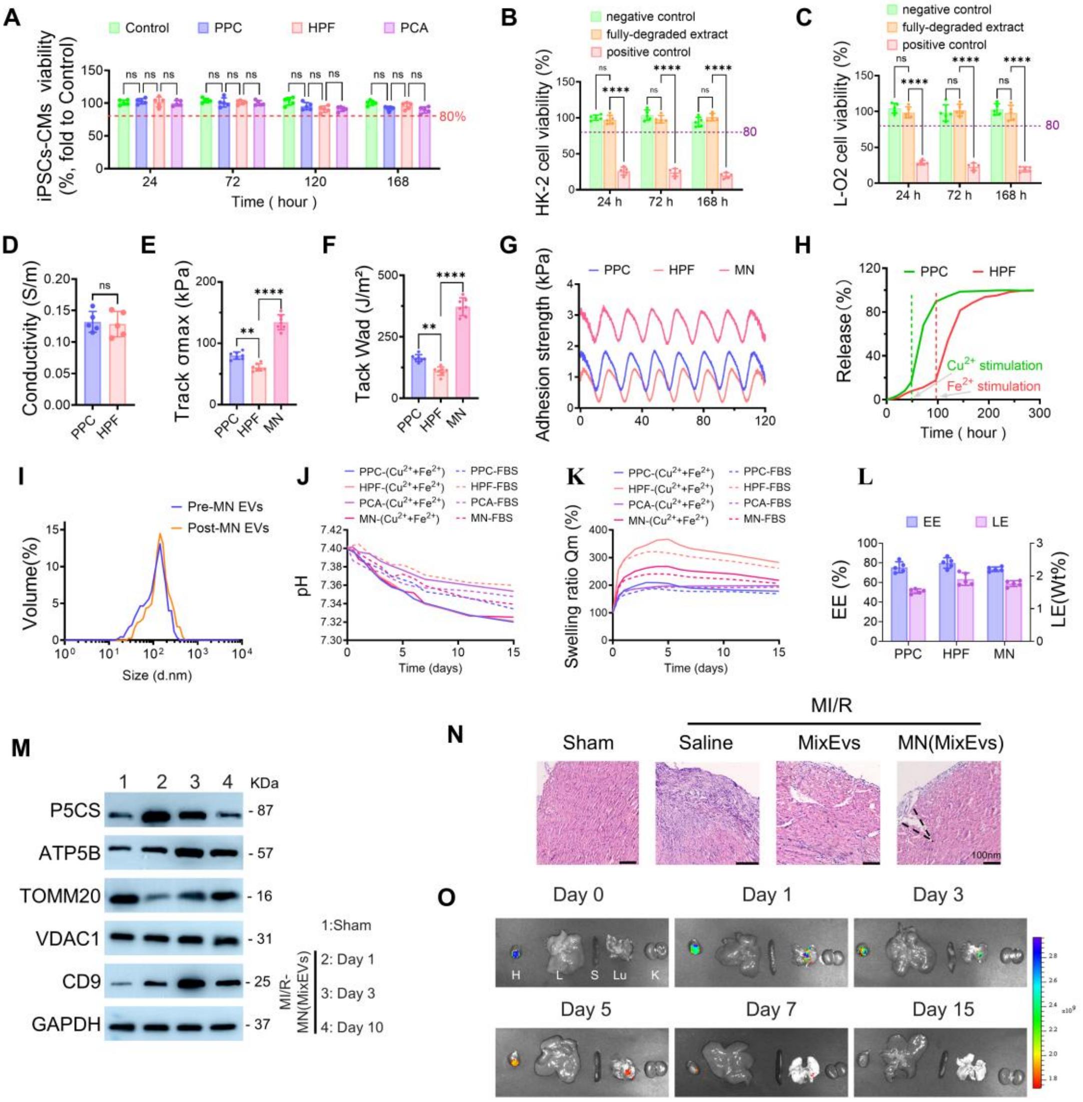

Figure S7： **(A)** Cardiomyocyte biocompatibility upon in situ direct contact. iPSC-CMs were co-cultured with hydrogel layers (PPC, HPF, and PCA) for 168 h. Kinetic CCK-8 assays demonstrated that cell viability remained consistently above 80% (red dashed line), confirming that the intrinsic material structure exhibits no cardiotoxicity. **(B–C)** Systemic safety assessment simulating "worst-case exposure." LO-2 **(B)** and HK-2 **(C)** cells were cultured for 168 h in fully degraded microneedle extracts containing pathological concentrations of metal ions. Survival rates remained significantly above the safety threshold, ruling out the risk of liver or kidney injury induced by degradation products or metal ions. **(D)** The conductivity of PPC/HPF hydrogels matches that of myocardial tissue. **(E–G)** Quantitative verification of the microneedle patch's superior wet tissue adhesion capability via **(E)** peak adhesion stress (Track σ_{max}), **(F)**work of adhesion (Tack W_{ad}), and **(G)**cyclic adhesion strength tests. **(H)** Release kinetics of exosomes from PPC or HPF layers under Cu^{2+} or Fe^{2+} stimulation, respectively, demonstrating that the system exhibits ion-triggered burst release behavior. **(I)** DLS analysis showing highly consistent particle size distributions of EVs before and after release, with no aggregation observed. **(J–K)** Monitoring of physicochemical stability. **(J)** The pH value remained within the neutral physiological range during degradation. **(K)** Swelling profiles demonstrated the volumetric stability of the material in a liquid environment. **(L)** LE and EE data confirm the high efficiency of the vesicle loading process. **(M)** Verification of molecular delivery in a large animal model in vivo. Western blot analysis was performed on Bama minipig myocardial tissues sampled at various time points to detect temporal changes in proteins including P5CS, ATP5B, TOMM20, VDAC1, and CD9. **(N)** Representative histological images of epicardial injection (EI) versus microneedle (MN) insertion into the epicardium and myocardial tissue. **(O)** In vivo fluorescence imaging of Rhodamine-labeled exosomes following the application of microneedles without the PCA barrier layer on rat hearts. Exosome signals were detected in both heart and lung tissues, indicating off-target distribution in the absence of the PCA barrier. **Statistics**: Data are presented as mean ± SD; the independent sample size (*n*) is indicated by data points/labels in the figures. One-way ANOVA with Tukey's multiple-comparison correction was used for single-factor multi-group comparisons. Two-way ANOVA with Sidak's or Tukey's multiple-comparison correction was used for two-factor designs. $*P < 0.05$, $**P < 0.01$, $***P < 0.005$, $****P < 0.001$; *ns*, not significant.

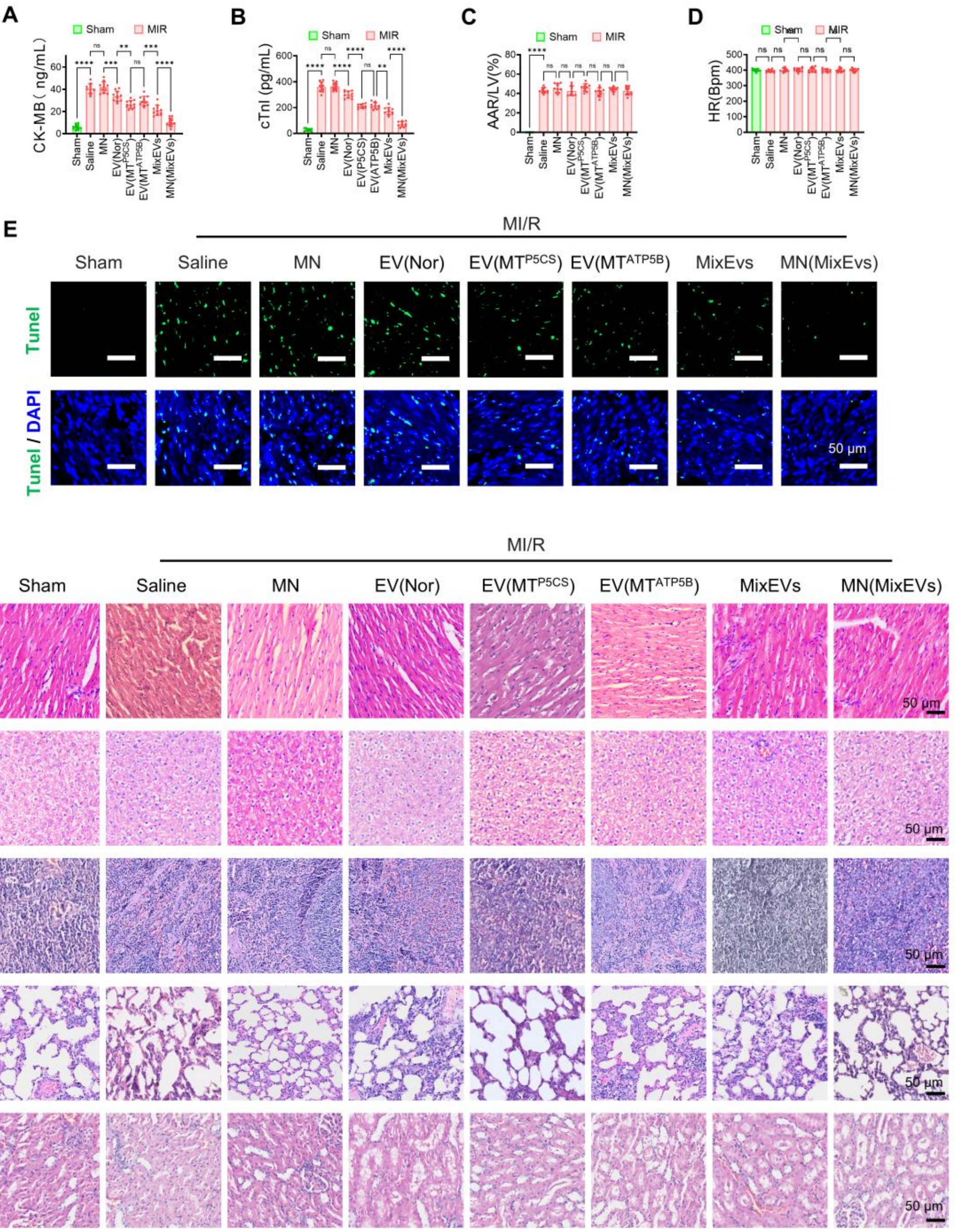

Figure S8: **(A–B)** Acute postoperative serum biochemical analysis: **(A)** CK-MB levels and **(B)** cTnI levels. The MN(MixEVs) group showed a significant reduction in these markers, indicating alleviated acute myocardial injury. **(C)** No significant difference was observed in the ratio of the Area at Risk to the Left Ventricle (AAR/LV) across groups, confirming the consistency of the surgical models. **(D)** Postoperative heart rate (HR) monitoring revealed no significant fluctuations across groups, indicating that MN(MixEVs) treatment exerted no adverse effects on heart rate. **(E)** TUNEL staining images demonstrated a marked reduction in apoptotic cells within the infarcted myocardium following MN(MixEVs) treatment (Green: TUNEL-positive; Blue: DAPI nuclear staining). Scale bar: 50 μm. **(F)**

H&E staining of major organs (heart, liver, spleen, lung, and kidney) revealed no structural damage or inflammatory cell infiltration, further verifying the excellent systemic biocompatibility of the MN(MixEVs) platform. Scale bar: 50 μm. **Statistics**: Data are presented as mean ± SD; the independent sample size (*n*) is indicated by data points/labels in the figures. One-way ANOVA with Tukey's multiple-comparison correction was used for single-factor multi-group comparisons. Two-way ANOVA with Sidak's or Tukey's multiple-comparison correction was used for two-factor designs. *$P < 0.05$, **$P < 0.01$, ***$P < 0.005$, ****$P < 0.001$; *ns*, not significant.

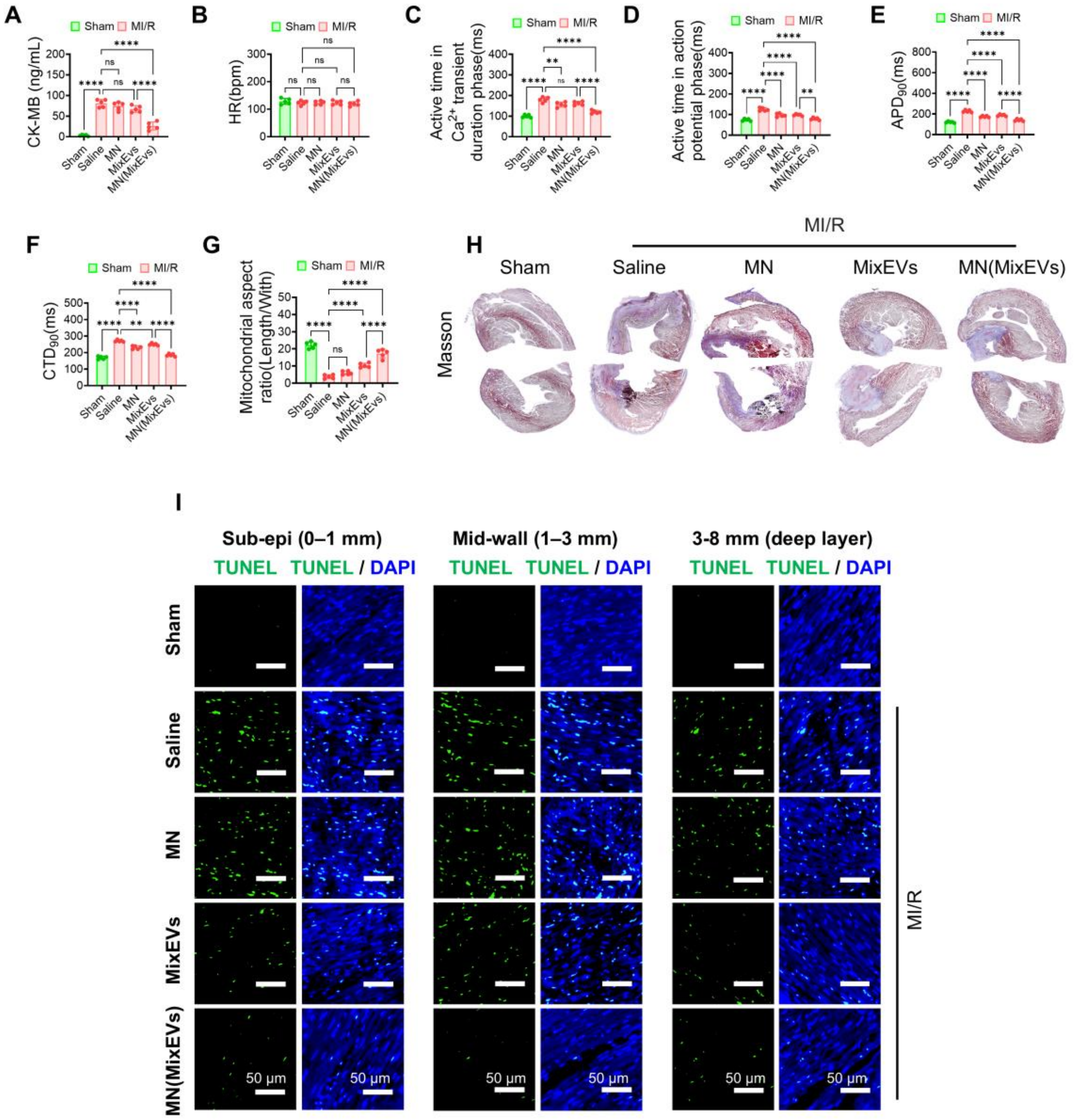

Figure S9: **(A)** Serum CK-MB levels measured 24h post-treatment. The MN(MixEvs) group exhibited a significant reduction, indicating alleviated myocardial injury. **(B)** Echocardiographic assessment revealed no significant differences in heart rate (HR) across groups, demonstrating that MN(MixEvs)

treatment exerted no adverse effects on heart rate. **(C–F)** Quantitative analysis of electrophysiological parameters: **(C)** activation time; **(D)** APD90; (E) APD_{90}; and (F) CTD_{90}. Significant functional improvement was observed in the MN(MixEvs) group. **(G)** Quantitative assessment of mitochondrial aspect ratio based on TEM images. The results confirmed that the MN(MixEvs) group effectively preserved mitochondrial fusion morphology and structural integrity. **(H)** Representative Masson's trichrome staining images of Bama minipig myocardium at 28 d post-MI/R. **(I)** Representative TUNEL staining images of stratified myocardial tissues (0–1 mm, 1–3 mm, and 3–8 mm) harvested from Bama minipigs 24 h post-MI/R. In contrast to the MixEvs group, which exhibited a trend of diminishing anti-apoptotic efficacy in the deep layer (3–8 mm), the MN(MixEvs) group maintained consistently low TUNEL-positive signals across all layers (from superficial to deep). This visually confirms that the microneedle delivery strategy overcame diffusion barriers, achieving effective transmural protection of the ventricular wall. **Statistics**: Data are presented as mean ± SD; the independent sample size (*n*) is indicated by data points/labels in the figures. One-way ANOVA with Tukey's multiple-comparison correction was used for single-factor multi-group comparisons. Two-way ANOVA with Sidak's or Tukey's multiple-comparison correction was used for two-factor designs. $^{*}P < 0.05$, $^{**}P < 0.01$, $^{***}P < 0.005$, $^{****}P < 0.001$; *ns*, not significant.

A

Group	Sham	Saline	MN	MixEVs	MN(MixEVs)
HR (bpm)	96.33 ± 4.62	101.33 ± 9.81	94.00 ± 7.21	96.67 ± 4.16	91.33 ± 4.04
LVIDd (mm)	30.72 ± 0.70	36.34 ± 1.00	35.91 ± 0.74	33.49 ± 1.17	31.69 ± 1.04
LVIDs (mm)	16.51 ± 0.67	31.90 ± 1.15	29.04 ± 2.81	24.14 ± 2.46	20.47 ± 2.59
LVEDV (mL)	41.35 ± 1.35	70.97 ± 3.07	60.48 ± 1.06	55.48 ± 0.96	47.92 ± 2.26
LVESV (mL)	14.04 ± 0.87	53.05 ± 3.93	40.40 ± 2.73	33.65 ± 2.86	22.67 ± 2.35
SV (mL)	26.97 ± 1.72	18.05 ± 2.76	19.80 ± 3.74	21.83 ± 3.82	25.25 ± 2.63
CO (L/min)	2.34 ± 0.50	1.81 ± 0.47	1.87 ± 0.19	2.11 ± 0.33	2.31 ± 0.31
EF (%)	66.65 ± 1.79	25.56 ± 4.74	32.42 ± 4.65	39.16 ± 5.40	52.76 ± 4.58
FS (%)	45.05 ± 2.28	12.55 ± 3.38	15.78 ± 6.48	27.34 ± 6.92	34.28 ± 7.42
E/A (ratio)	1.61 ± 0.23	0.85 ± 0.12	0.90 ± 0.13	1.10 ± 0.13	1.34 ± 0.10

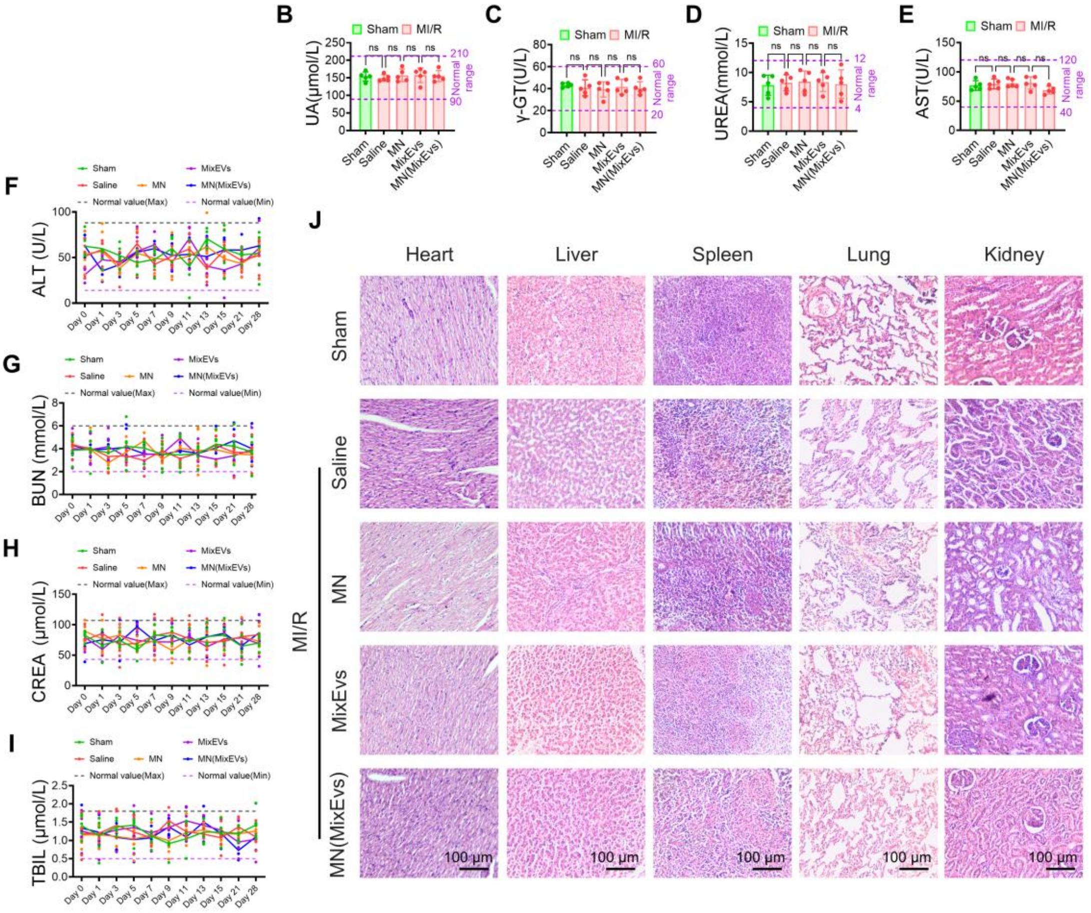

Figure S10: **(A)** Statistical summary of echocardiographic parameters at 28 d post-operation. The data demonstrate that the MN(MixEvs) group significantly outperformed the control group in key hemodynamic indices, including left ventricular internal diameter at end-diastole/end-systole (LVIDd/s) and ejection fraction (EF). **(B–E)** Acute postoperative serum biochemical analysis at 24h: **(B)** UA; **(C)** γ-GT; **(D)** UREA; and **(E)** AST. All values fell within the normal physiological range (indicated by dashed lines), suggesting no induction of acute metabolic toxicity. **(F–I)** Dynamic monitoring of liver and kidney function over the full 28-day postoperative period: **(F)** ALT; **(G)** BUN; **(H)** CREA; and **(I)**

TBIL) Time-concentration curves show that all indicators remained stably distributed within reference limits throughout the treatment course, ruling out drug-induced cumulative toxicity. **(J)** Histopathological assessment via H&E staining of major organs (heart, liver, spleen, lung, and kidney) at 28 d post-operation. Images reveal normal tissue morphology with no evidence of necrosis or inflammatory cell infiltration, confirming the excellent biocompatibility of the MN(MixEvs) platform. Scale bar: 100 μm. **Statistics**: Data are presented as mean ± SD; the independent sample size (*n*) is indicated by data points/labels in the figures. One-way ANOVA with Tukey's multiple-comparison correction was used for single-factor multi-group comparisons. Two-way ANOVA with Sidak's or Tukey's multiple-comparison correction was used for two-factor designs. *$P < 0.05$, **$P < 0.01$, ***$P < 0.005$, ****$P < 0.001$; *ns*, not significant.

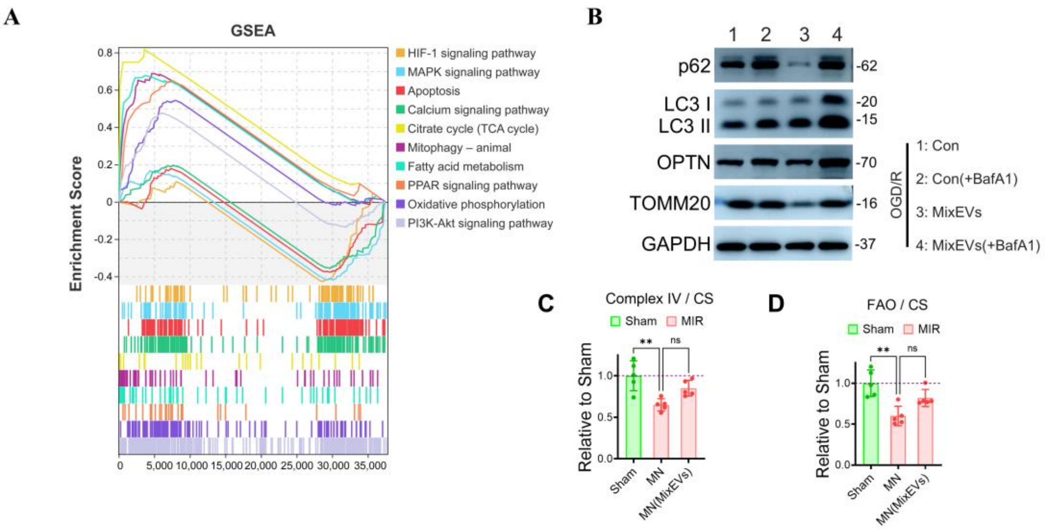

Figure S11: **(A)** GSEA further confirmed that the MN(MixEvs) treatment group showed significant enrichment in gene sets related to mitochondrial metabolism, while concurrently suppressing signals associated with hypoxia and apoptosis. This provides a molecular basis for subsequent mechanistic verification. **(B)** In the iPSC-CM OGD/R model, Bafilomycin A1 blockade experiments demonstrated that the MixEVs group exhibited more pronounced LC3-II accumulation and substrate turnover, thereby confirming a substantial enhancement of autophagic flux. **(C–D)** Quantitative assessment of downstream mitochondrial metabolic activity. Myocardial homogenates from the AAR border zone of Bama minipigs were collected 24h post-operation. Relative enzymatic activity levels of Complex IV **(C)** and fatty acid oxidation (FAO) **(D)** were analyzed following citrate synthase (CS) normalization to evaluate the

functional status of key metabolic modules downstream of the respiratory chain. Data are presented as mean ± SD. Sample sizes (*n*) for independent experiments are indicated by data points or labels within the figure. Statistical analysis methods are as previously described.
